# Long-term Effectiveness of Adjuvant Treatment With Catechol-O-Methyltransferase or Monoamine Oxidase B Inhibitors Compared With Dopamine Agonists Among Patients With Parkinson Disease Uncontrolled by Levodopa Therapy: The PD MED Randomized Clinical Trial

**DOI:** 10.1001/jamaneurol.2021.4736

**Published:** 2021-12-28

**Authors:** Richard Gray, Smitaa Patel, Natalie Ives, Caroline Rick, Rebecca Woolley, Sharon Muzerengi, Alastair Gray, Crispin Jenkinson, Emma McIntosh, Keith Wheatley, Adrian Williams, C. E. Clarke

**Affiliations:** 1 Nuffield Department of Population Health, University of Oxford, Oxford, United Kingdom; 2 Birmingham Clinical Trials Unit, Institute of Applied Health Research, University of Birmingham, Birmingham, United Kingdom; 3 Nottingham Clinical Trials Unit, University of Nottingham, Nottingham, United Kingdom; 4 Department of Neurology, University Hospital, Birmingham, United Kingdom; 5 Health Economics and Health Technology Assessment, University of Glasgow, Glasgow, United Kingdom; 6 Department of Neurology, Sandwell and West Birmingham Hospitals NHS Trust, Birmingham, United Kingdom; 7 Cancer Research UK Clinical Trials Unit, Institute of Cancer and Genomic Sciences. University of Birmingham, Birmingham, UK

## Abstract

**Importance:**

Many people with Parkinson disease (PD) develop motor complications that are uncontrolled by levodopa dose adjustment. Among these patients, it is uncertain which drug class is more effective as adjuvant therapy.

**Objective:**

To compare the long-term effects on patient-rated quality of life of adding a dopamine agonist vs a dopamine reuptake inhibitor (DRI), either a monoamine oxidase type B (MAO-B) inhibitor or a catechol-O-methyltransferase (COMT) inhibitor, to levodopa therapy for the treatment of patients with motor complications of PD.

**Design, Setting, and Participants:**

This pragmatic semifactorial (2 × 1) randomized clinical trial recruited from 64 neurology and geriatric clinics (62 in the United Kingdom, 1 in the Czech Republic, and 1 in Russia) between February 23, 2001, and December 15, 2009. A total of 500 patients with idiopathic PD who developed uncontrolled motor complications and did not have dementia were randomly assigned on a 1:1:1 basis using a computerized minimization program. Data were analyzed between 2017 and 2020.

**Interventions:**

Open-label dopamine agonist, MAO-B inhibitor, or COMT inhibitor.

**Main Outcomes and Measures:**

Primary outcomes were scores on the 39-item Parkinson’s Disease Questionnaire (PDQ-39) mobility domain and cost-effectiveness. Outcomes were assessed before study entry, at 6 and 12 months after randomization, and annually thereafter. Repeated-measures and log rank analyses were used in an intention-to-treat approach.

**Results:**

Among 500 participants, the mean (SD) age was 73.0 (8.2) years; 314 participants (62.8%) were men. Over a median of 4.5 years (range, 0-13.3 years) of follow-up, the participants in the dopamine agonist group had a mean PDQ-39 mobility score that was 2.4 points (95% CI, −1.3 to 6.0 points) better than that of the combined MAO-B and COMT groups; however, this difference was not significant (*P*=.20). With regard to DRIs, participants in the MAO-B group had mean PDQ-39 mobility scores that were 4.2 points (95% CI, 0.4-7.9 points; *P*=.03) better than those of the COMT group and EuroQol 5-dimension 3-level (EQ-5D-3L) utility scores that were 0.05 points (95% CI, 0.003-0.09 points; *P*=.04) better than the COMT group. Nonsignificant improvements were found in the PDQ-39 summary index (mean difference, 2.2 points; 95% CI, −0.2 to 4.5 points; *P*=.07) along with nonsignificant reductions in dementia (rate ratio [RR], 0.70; 95% CI, 0.47-1.03; *P* = .07) and mortality (RR, 0.76; 95% CI, 0.56-1.03; *P*=.07). When dopamine agonists were compared with MAO-B inhibitors only, the outcomes were similar.

**Conclusions and Relevance:**

In this study, patient-rated quality of life was inferior when COMT inhibitors were used as adjuvant treatment compared with MAO-B inhibitors or dopamine agonists among people with PD who experienced motor complications that were uncontrolled by levodopa therapy. The MAO-B inhibitors produced equivalent disease control, suggesting that these agents may be underused as adjuvant therapy.

**Trial Registration:**

isrctn.org Identifier: ISRCTN69812316; EU Clinical Trials Register Identifier: 2005-001813-16

## Key Points

**Question:**

Is adding a dopamine reuptake inhibitor (DRI), either a monoamine oxidase type B (MAO-B) inhibitor or a catechol-O-methyltransferase (COMT) inhibitor, to levodopa therapy more effective than adding a dopamine agonist and, if a DRI is more effective, which DRI class (MAO-B or COMT) is preferable for improving patient-rated quality of life among those with motor complications of Parkinson disease (PD) that are uncontrolled by levodopa therapy?

**Findings:**

In this randomized clinical trial involving 500 people with PD, no statistically significant difference was found in Parkinson’s Disease Questionnaire mobility scores between adjuvant therapy with dopamine agonists vs DRIs; however, scores were a mean of 4.2 points better with MAO-B inhibitors compared with COMT inhibitors.

**Meaning:**

In this study, patient-rated quality of life was worse with the addition of COMT inhibitors compared with MAO-B inhibitors or dopamine agonists as adjuvant treatment for people with PD uncontrolled by levodopa therapy.

## Teaser

This randomized clinical trial assesses the long-term effects on patient-rated quality of life of adjuvant treatment with dopamine reuptake inhibitors compared with dopamine agonists among patients with motor complications of Parkinson disease that are inadequately controlled by levodopa therapy.

## Introduction

Levodopa is the most commonly used and effective initial treatment for Parkinson disease (PD).^1^ However, after prolonged or high-dose use, motor complications, such as abnormal involuntary movements (dyskinesia) and motor fluctuations (premature decrease in the drug’s effects and unpredictable switching between on and off phases), can develop. Another drug class can then be added to reduce motor complications and levodopa dose. The most widely used adjuvant drugs are dopamine agonists and dopamine reuptake inhibitors (DRIs), such as catechol-O-methyltransferase (COMT) inhibitors and monoamine oxidase type B (MAO-B) inhibitors.

Studies comparing brief treatment with these drugs vs placebo among patients with motor complications have reported that each drug can improve motor function and activities of daily living scores.^2,3^ However, the occurrence of dyskinesia and numerous other adverse events (AEs) increased. Indirect comparisons between placebo-controlled clinical trials have suggested greater efficacy for dopamine agonists vs DRIs, such as COMT or MAO-B inhibitors, which appear to have comparable efficacy. However, such indirect comparisons can be misleading. Direct head-to-head randomized clinical trials are needed for reliable assessment of the comparative clinical benefits and cost-effectiveness of different adjuvant therapies; however, we have not identified any clinical trials comparing modern agents. Hence, it is unclear which class of drug is preferable for use as adjuvant therapy.^4^

The present Parkinson Disease Medication (PD MED) study was a large pragmatic real-life randomized clinical trial addressing 2 questions about people with PD who experience motor complications: (1) with respect to quality of life and cost-effectiveness, is a DRI, either an MAO-B or COMT inhibitor, or a dopamine agonist more effective as adjuvant treatment for patients receiving levodopa therapy? and (2) if a DRI is more effective, which drug class (COMT or MAO-B) is preferable?

## Methods

### Study Design and Participants

The PD MED clinical trial has compared drug classes used as initial^1^ and adjuvant treatment among people with PD; the present study was a multicenter open-label pragmatic semifactorial (2 × 1) randomized clinical trial that addressed adjuvant treatment only. Patients with idiopathic PD, which was diagnosed by movement disorder specialists using UK Brain Bank criteria,^5^ were eligible for inclusion if they developed motor complications that were uncontrolled by levodopa therapy (alone or in combination with either a dopamine agonist or an MAO-B inhibitor) and hence required the addition of another class of drug (but uncertainty^6^ existed regarding which drug class to use). Patients were ineligible if they had dementia (as defined by the medical team responsible) or were unable to provide informed consent for participation or complete study questionnaires. All participants provided written informed consent before randomization. Study approval was obtained from the West Midlands Research Ethics Committee, the UK Medicines and Healthcare products Regulatory Agency, local ethics committees, and participating hospitals. This study followed the Consolidated Standards of Reporting Trials (CONSORT) reporting guideline for randomized clinical trials. The trial protocol is available in Supplement 1.

### Randomization and Treatment Allocation

**Figure 1.**
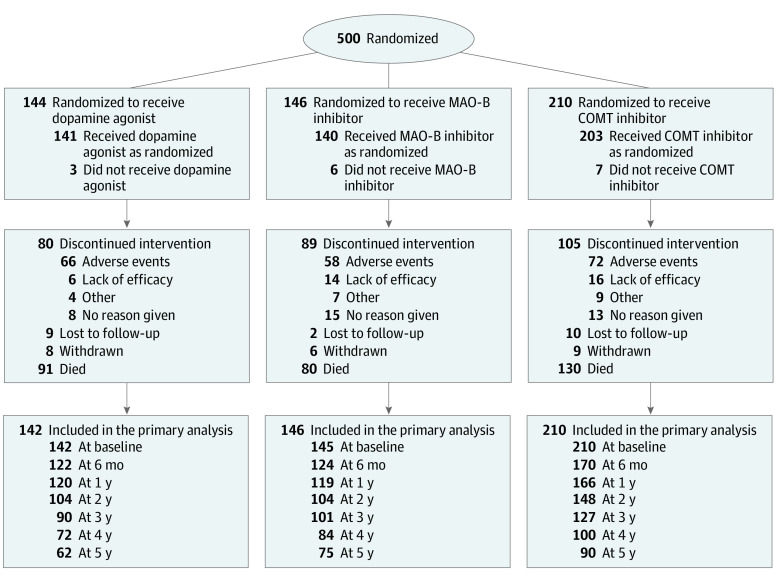
CONSORT Diagram Some patients had more than 1 reason (typically adverse events and lack of efficacy) for discontinuing treatment with the drug class as randomized; many study withdrawals classified as *other reasons* were due to comorbidity. A total of 14 patients who were lost to follow-up were from 2 centers (1 in Russia and 1 in the Czech Republic); of those, 7 patients were in the COMT (catechol-O-methyltransferase) group, 6 were in the dopamine agonist group, and 1 was in the MAO-B (monoamine oxidase type B) group. A total of 17 patients who withdrew from the study or were lost to follow-up later died (9 in the COMT group, 6 in the dopamine agonist group, and 2 in the MAO-B group).

Between February 23, 2001, and December 15, 2009, 500 people with later-stage PD were randomized from 62 neurology and geriatric clinics in England, Scotland, and Wales and 2 clinics outside the UK (1 in the Czech Republic and 1 in Russia) (Figure 1; eFigure 1 in Supplement 2). Patients were randomized 1:1:1 to receive a dopamine agonist, a COMT inhibitor, or an MAO-B inhibitor during a telephone call to the University of Birmingham Clinical Trials Unit. Patients who were already receiving a dopamine agonist when uncontrolled motor complications occurred could be randomized only between a COMT inhibitor or an MAO-B inhibitor. Patients receiving an MAO-B inhibitor when uncontrolled motor complications occurred and patients for whom the clinician considered an MAO-B inhibitor to be definitely contraindicated could be randomized only between a COMT inhibitor or a dopamine agonist. Randomizations were minimized within strata defined by Hoehn and Yahr disease stage,^7^ time since diagnosis (<4 years, 4-6 years, and ≥6 years), previous therapy received (dopamine agonist, MAO-B inhibitor, or neither), and age (<50 years, 50-59 years, 60-69 years, 70-79 years, and ≥80 years). Blinding of randomized treatment group was not practicable because the study was long term and required frequent dose adjustment. In addition, blinding was not considered essential because all patients received active treatment; thus, the potential for subjectively biased assessment was small.

### Intervention

Clinicians could initiate open-label treatment with whichever drug they considered appropriate within the patient’s randomized drug class; they could also switch to another agent in the same drug class and titrate doses as they deemed necessary in the best interests of the patient. If, despite this approach, symptoms were not controlled or patients experienced intolerable AEs, investigators could add or substitute a new agent from another drug class. Follow-up assessments were requested irrespective of treatment adherence to allow intention-to-treat analyses.

### Outcomes

One primary outcome was functional status using the mobility domain of the patient-rated 39-item Parkinson’s Disease Questionnaire (PDQ-39; score range, 0-100 points, with higher scores indicating greater difficulty).^8,9^ A second primary outcome, for which data will be reported in a separate article, was cost per quality-adjusted life-year (QALY) derived from the EuroQol 5-dimension 3-level (EQ-5D-3L) generic quality of life measure (score range, −0.59 to 1.00 points, with higher scores indicating better health-related quality of life),^10^ a health and social care resource use questionnaire, and the England and Wales Hospital Episodes Statistics (HES) database.^11^

Secondary outcomes included scores on other PDQ-39 domains, overall PDQ-39 score (summary index), Hoehn and Yahr disease stage (stages I-V, with stage I indicating unilateral involvement only and stage V indicating confinement to bed or wheelchair),^7^ adherence to treatment, cognitive ability assessed by the Mini-Mental State Examination (score range, 0-30 points, with higher scores indicating better cognitive ability),^12^ onset of dementia, permanent admissions to the hospital or institutional care, and death. Caregivers rated their own well-being using the 36-item Short Form (SF-36) Health Survey, version 2 (score range, 0-100 points, with higher scores indicating better state of health).^13^ Patients and caregivers completed study forms before randomization and by mail at 6 months, 1 year, and annually thereafter. The Mini-Mental State Examination was administered at baseline, then every 5 years. Information about disease status (Hoehn and Yahr stage and any change in PD diagnosis), treatment adherence, AEs, dementia diagnoses, and admissions to institutional care was collected systematically at annual clinical assessments.

Deaths and unexpected serious AEs believed to be associated with clinical trial treatments were reported on serious AE forms. Deaths were monitored through NHS Digital^14^ (the trading name of the Health and Social Care Information Centre, which provided death registration from the United Kingdom National Health Service [NHS]); NHS Digital also provided HES data covering inpatient episodes from April 1, 1999, to March 31, 2013. Data from the HES were linked using clinical trial number, month and year of birth, sex, and the first part of the patient’s postal code. Hospital admissions were classified as potentially PD-related if coded as PD, neuropsychiatric disorders (eg, hallucinations), nonmotor symptoms (eg, constipation), blood pressure–related disorders (eg, hypotension), infections (eg, urinary tract infection and pneumonia), and/or falls, fractures, and injuries.

### Statistical Analysis

The PD MED study was powered to detect a 6-point difference (considered at the time to be the minimum clinically important difference^8^) in the PDQ-39 mobility domain score between groups. Assuming an SD of 18.6 points (2-sided *P* value of .05 and 80% power), this power required randomization of 155 patients to each arm. To allow for 10% withdrawal and for 2-way as well as 3-way randomization, we planned to recruit 500 patients. Interim analyses of efficacy and safety data were reviewed annually by an independent data monitoring committee.

Data were analyzed based on the drug class to which the patient was randomized, regardless of whether the patient adhered to this treatment. For between-group comparisons of drug dosage, levodopa equivalent doses for other drugs were calculated using the formulas derived from a systematic review.^15^ The primary data analyses of PDQ-39 scores, Hoehn and Yahr stages, SF-36 scores, and EQ-5D-3L scores used mixed-effects repeated-measures models to assess the mean difference between treatments over 5 years and to test whether this difference increased over time. Baseline scores were included in the model as covariates. Missing values in PDQ-39 domain scores were imputed using an expectation maximization algorithm.^16,17^ Changes in Mini-Mental State Examination scores were compared using 2-sided paired *t* tests. Log-rank analyses were used to compare rates of dementia, hospital admission, entry to institutional care, and death and to estimate first event rate ratios (RRs) and their 95% CIs, which were displayed as Kaplan-Meier survival curves. The incidence of AEs was compared using Fisher exact or χ^2^ tests as appropriate. Variability of treatment effect across protocol-specified stratification parameters was assessed by tests of heterogeneity or trend. The number and duration of admissions were compared using a negative binomial model, adjusted for the number of years in the clinical trial, baseline Hoehn and Yahr stage, baseline disease duration, sex, and institutionalization status. Data were analyzed between 2017 and 2020, using SAS software, version 9.4 (SAS Institute Inc), and Stata SE software, version 14 (StataCorp LLC). The significance threshold was set at *P* = .05.

## Results

### Study Population

Among 500 total participants (mean [SD] age, 73.0 [8.2] years; 314 men [62.8%] and 186 women [37.2%]), 236 were entered in the 3-way randomization to receive either a dopamine agonist, an MAO-B inhibitor, or a COMT inhibitor. A total of 264 participants were entered in the 2-way randomizations, with 134 participants randomized to receive either an MAO-B inhibitor or a COMT inhibitor and 130 participants randomized to receive either a dopamine agonist or a COMT inhibitor. At randomization, all 236 participants in the 3-way randomization groups were receiving levodopa alone. Among those in the 2-way randomization groups, 133 of 134 participants (99.3%) in the MAO-B vs COMT groups were receiving dopamine agonists at randomization and 63 of 130 participants (48.5%) in the dopamine agonist vs COMT groups were receiving an MAO-B inhibitor in addition to levodopa therapy at randomization (eTable 1 in Supplement 2). Patient characteristics at entry were balanced between treatment groups within each randomization strata. Data on race and ethnicity were not collected because the study had inadequate statistical power to assess any variability in efficacy among racial and ethnic groups.

The intended agents for initial use among 366 participants randomized to receive a dopamine agonist were ropinirole (158 participants [43.2%]), pramipexole (128 participants [35.0%]), and other agents (80 participants [21.9%]). The intended agents for initial use among 370 participants randomized to receive MAO-B inhibitors were oral selegiline (191 participants [51.6%]), sublingual selegiline (51 participants [13.8%]), rasagiline (109 participants [29.5%]), and unknown agents (19 participants [5.1%]). Entacapone was the chosen agent for initial use among most of the 500 participants randomized to receive a COMT inhibitor (454 participants [90.8%]), followed by an entacapone-levodopa conjugate (Stalevo [Novartis]; 34 participants [6.8%]) and another or unknown agent (12 participants [2.4%]).

### Adherence With Randomized Intervention

**Figure 2.**
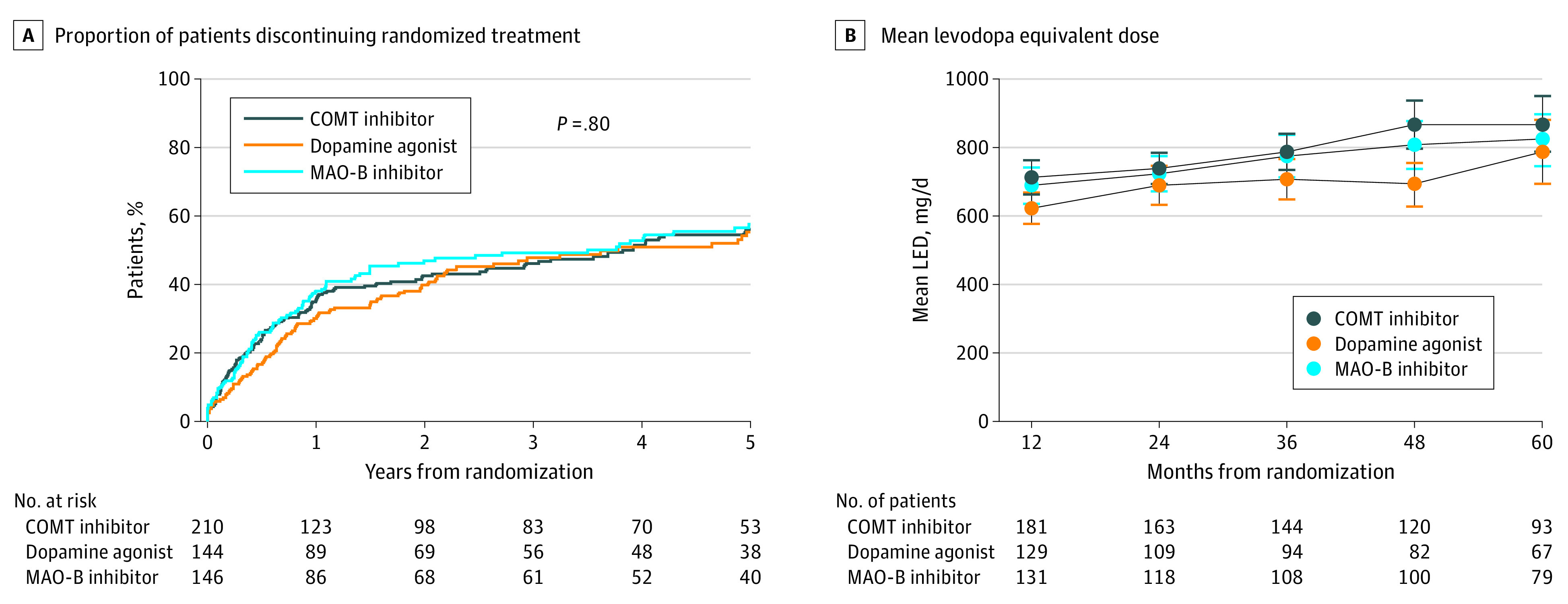
Proportion of Patients Discontinuing Treatment Over 5 Years of Follow-up and Mean Levodopa Equivalent Dose COMT indicates catechol-O-methyltransferase; LED, levodopa equivalent dose; and MAO-B, monoamine oxidase type B.

Drug withdrawal rates were comparable across drug classes (Figure 2A). Among those who received a dopamine agonist, 30% discontinued treatment by 1 year, and 55% discontinued treatment by 5 years. Among those who received an MAO-B inhibitor, 38% discontinued treatment by 1 year, and 58% discontinued treatment by 5 years. Among those who received a COMT inhibitor, 36% discontinued treatment after 1 year, and 56% discontinued treatment after 5 years. The only baseline factor associated with treatment adherence was older age; 218 of 355 participants (61.4%) who were 70 years and older vs 72 of 145 participants (49.7%) who were younger than 70 years discontinued treatment with their randomized drug class (*P* = .02). Adverse events were the predominant reason for withdrawal in all drug classes (66 participants receiving dopamine agonists, 58 receiving MAO-B inhibitors, and 72 receiving COMT inhibitors) (eTable 2 in Supplement 2). Those AEs were mainly psychiatric (eg, mental problems, such as psychosis or confusion) among those receiving dopamine agonists (45 participants) and MAO-B inhibitors (24 participants) and were mainly gastrointestinal among those receiving entacapone (29 participants). The levodopa equivalent dose was slightly higher in the MAO-B and COMT arms (mean [SD], 818 [333] mg/day and 868 [397] mg/day, respectively) vs the dopamine agonist arm (mean [SD], 788 [385] mg/day) (Figure 2B), but the mean levodopa doses were similar (mean [SD], 628 [330] mg/day and 594 [228] mg/day vs 609 [274] mg/day) (eFigure 2 in Supplement 2).

Return rates for PDQ-39 forms were 99% at baseline, 86% at 6 months, 88% at 1 year, 84% at 2 years, 81% at 3 years, 74% at 4 years, and 76% at 5 years, with no significant differences between groups.

### Outcome Measures

**Figure 3.**
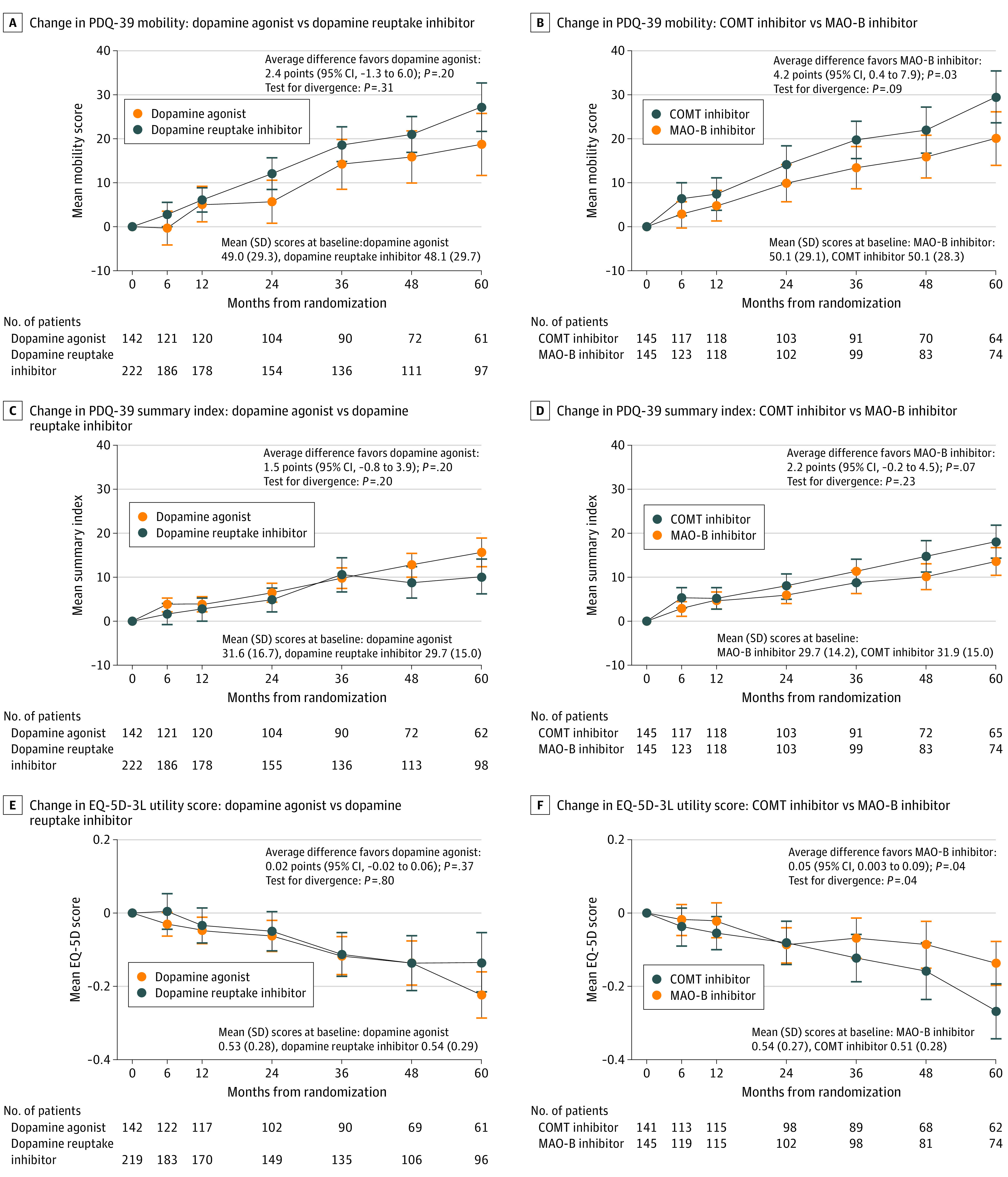
Change in Outcome Measures From Baseline to 5 Years by Treatment The mean differences and 95% CIs for all panels are reported in Outcome Measures in the Results section. Panels A, C, and E combine data from the 3-way and 2-way randomizations. COMT indicates catechol-O-methyltransferase; EQ-5D-3L, EuroQol 5-dimension 3-level measure; MAO-B, monoamine oxidase type B; and PDQ-39, 39-item Parkinson’s Disease Questionnaire.

**Figure 4.**
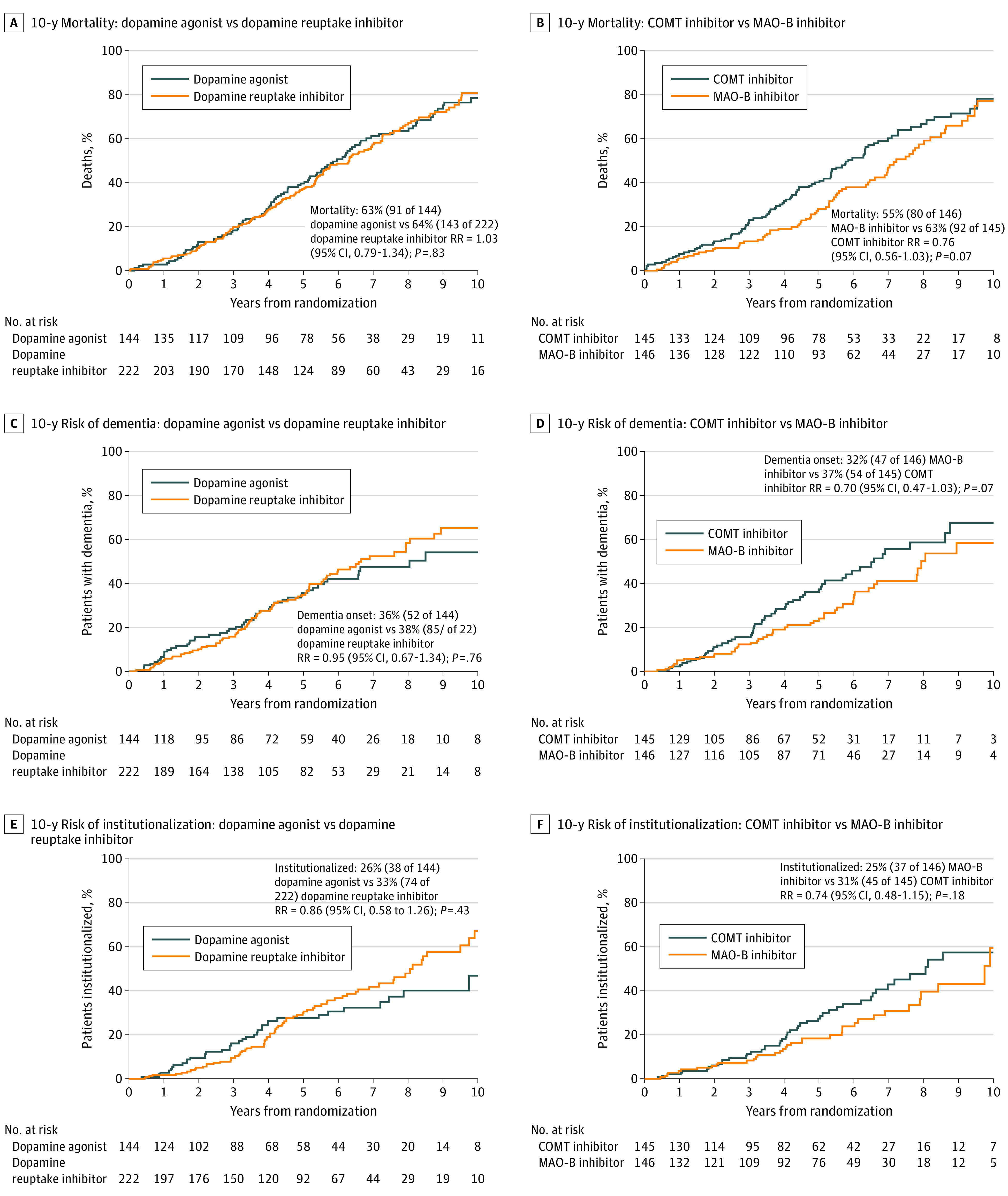
Ten-Year Mortality, Risk of Dementia, and Risk of Institutionalization by Treatment The risk ratios and 95% CIs for all panels are reported in Outcome Measures in the Results section. Panels A, C, and E depict combined data from the 3-way and 2-way randomizations. COMT indicates catechol-O-methyltransferase; and MAO-B, monoamine oxidase type B.

After a median of 4.5 years (range, 0-13.3 years) of follow-up, the dopamine agonist group had PDQ-39 mobility scores that were a mean of 2.4 points (95% CI, −1.3 to 6.0 points) better than the combined MAO-B and COMT groups; however, this difference was not significant (*P* = .20) (Figure 3A). No significant differences were found in any other PDQ-39 domain (eg, activities of daily living: mean difference, 3.4 points; 95% CI, −0.2 to 6.9; *P* = .07), the PDQ-39 summary index (mean difference, 1.5 points; 95% CI, −0.8 to 3.9 points; *P* = .20), the EQ-5D-3L utility score (mean difference, 0.02 points; 95% CI, −0.02 to 0.06 points; *P* = .38), or the Mini-Mental State Examination score (mean difference, 1.77 points; 95% CI, −0.14 to 3.69 points; *P* = .07) (Table; Figure 3C and E). The rates of death, dementia, and institutionalization were also not significantly different between the dopamine agonist group and the combined MAO-B and COMT groups (mortality for dopamine agonist, 63% [91 of 144] vs for dopamine reuptake inhibitor, 64% [143 of 222]: RR, 1.03; 95% CI, 0.79-1.34; *P* = .83; dementia onset for dopamine agonist, 36% [52 of 144] vs for dopamine reuptake inhibitor, 38% [85 of 222]: RR, 0.95; 95% CI, 0.67-1.34; *P* = .76; institutionalization rate for dopamine agonist, 26% [38 of 144] vs for dopamine reuptake inhibitor, 33% [74 of 222]: RR, 0.86; 95% CI, 0.58-1.26; *P* = .43) (Figure 4A, C, and E).

**Table.** Estimated Mean Difference in Outcome Measures Between Treatment Groups

Outcome measure	Dopamine agonist vs DRI		MAO-B inhibitor vs COMT inhibitor		MID
	Estimated mean difference (95% CI)^a^	*P* value	Estimated mean difference (95% CI)^b^	*P* value	
PDQ-39 subscale^c^					
Mobility	2.4 (−1.3 to 6.0)	.20	4.2 (0.4 to 7.9)	.03	3.2
ADL	3.4 (−0.2 to 6.9)	.07	4.0 (0.4 to 7.5)	.03	4.4
Emotional well-being	2.2 (−1.2 to 5.5)	.21	4.4 (1.1 to 7.6)	.009	4.2
Stigma	−0.3 (−3.9 to 3.3)	.87	0.7 (−2.9 to 4.3)	.69	5.6
Social support	−0.5 (−3.0 to 2.1)	.73	3.7 (0.8 to 6.6)	.01	11.4
Cognition	1.0 (−2.2 to 4.3)	.53	2.5 (−1.0 to 6.1)	.16	1.8
Communication	−1.1 (−4.4 to 2.2)	.51	2.9 (−0.7 to 6.6)	.12	4.2
Bodily discomfort	1.7 (−1.8 to 5.2)	.35	−0.6 (−4.5 to 3.2)	.76	2.1
Summary index	1.5 (−0.8 to 3.9)	.20	2.2 (−0.2 to 4.5)	.07	1.6
Hoehn and Yahr stage^d^	−0.16 (−0.29 to −0.03)	.02	0.08 (−0.05 to 0.21)	.23	NA
EQ-5D-3L utility score^e^	0.02 (−0.02 to 0.06)	.38	0.05 (0.003 to 0.09)	.04	NA
MMSE at 5 y^f^	1.77 (−0.14 to 3.69)	.07	1.68 (−0.33 to 3.68)	.10	NA

Abbreviations: ADL, activities of daily living; COMT, catechol-O-methyltransferase; DRI, dopamine reuptake inhibitor; EQ-5D-3L, EuroQol 5-dimension 3-level survey; MAO-B, monoamine oxidase type B; MID, minimally important difference; MMSE, Mini-Mental State Examination; NA, not applicable; PDQ-39, 39-item Parkinson’s Disease Questionnaire.

^a^ Positive values favor dopamine agonist.

^b^ Positive values favor MAO-B inhibitor.

^c^ Score range, 0-100 points, with higher scores indicating greater difficulty.

^d^ Stages I-V, with stage I indicating unilateral involvement only and stage V indicating confinement to bed or wheelchair.

^e^ Score range, −0.59 to 1.00 points, with higher scores indicating better health-related quality of life.

^f^ Score range, 0-30 points, with higher scores indicating better cognitive ability.

In contrast, in the comparison between the MAO-B and COMT groups, a significant mean difference of 4.2 points (95% CI, 0.4-7.9 points; *P* = .03) in PDQ-39 mobility scores in favor of MAO-B inhibitors was observed (Figure 3B). Scores on the PDQ-39 summary index also favored MAO-B vs COMT inhibitors (mean difference, 2.2 points; 95% CI, −0.2 to 4.5 points; *P* = .07) (Figure 3D), as did other PDQ-39 domains, reaching statistical significance for activities of daily living (mean difference, 4.0 points; 95% CI, 0.4-7.5 points; *P* = .03), emotional well-being (mean difference, 4.4 points; 95% CI, 1.1-7.6 points; *P* = .009), and social support (mean difference, 3.7 points; 95% CI, 0.8-6.6 points; *P* = .01) (Table). The EQ-5D-3L utility score was a mean of 0.05 points (95% CI, 0.003-0.09 points; *P* = .04) better with receipt of MAO-B vs COMT inhibitors (Table; Figure 3F). No differences were found in caregiver well-being (as assessed by the SF-36) between the dopamine agonist group and the combined MAO-B and COMT groups (eg, mental component score: mean difference, −0.81 points; 95% CI, −3.11 to 1.48 points; *P* = .48) or between the MAO-B and COMT groups individually (eg, mental component score: mean difference, −0.53 points; 95% CI, −2.79 to 1.74 points; *P* = .65) (eTable 3 in Supplement 2).

Treatment benefit did not significantly increase over time in the MAO-B group vs the COMT group with regard to PDQ-39 mobility scores (Figure 3B) but did significantly increase over time for EQ-5D-3L scores (slopes diverging at a rate of 0.02 points per year [95% CI, 0.001-0.04 points per year] in favor of the MAO-B group) (Figure 3F). The rate of dementia onset was lower in the MAO-B group (32% [47 of 146]) compared with the COMT group (37% [54 of 145]) (RR, 0.70; 95% CI, 0.47-1.03; *P* = .07) (Figure 4D), as was the mortality rate for the MAO-B group (55% [80 of 146]) compared with the COMT group (63% [92 of 145]) (RR, 0.76; 95% CI, 0.56-1.03; *P* = .07) (Figure 4B) and the institutionalization rate for the MAO-B group (25% [37 of 146]) compared with the COMT group (31% [45 of 145]) (RR, 0.74; 95% CI, 0.48-1.15; *P* = .18) (Figure 4F), although none of these differences were significant.

In exploratory analyses restricted to patients randomized to the dopamine agonist and MAO-B groups, no differences were observed in PDQ-39 scores in either the mobility domain (mean difference, 0.2 points; 95% CI, −4.9 to 5.3 points; *P* = .93) or the summary index (mean difference, 0.1 points; 95% CI, −3.3 to 3.6 points; *P* = .94). Among patients receiving dopamine agonists vs COMT inhibitors, PDQ-39 mobility scores were a mean of 3.4 points (95% CI, −0.7 to 7.6 points) better in the dopamine agonist group vs the COMT group, but the difference was not significant (*P* = .10). Treatment efficacy, as measured by the PDQ-39 mobility subscale, did not differ according to baseline stratification variables (ie, age, duration of PD symptoms, Hoehn and Yahr stage, or randomized treatment) (eFigure 3 in Supplement 2).

### Adverse Events and Hospitalization

Nine unexpected serious AEs were reported among 9 participants, none of which were considered unexpected after review. Information on hospitalizations from the HES database was available for 469 of 475 patients (98.7%) from England and Wales, 341 of whom (72.7%) were admitted during the study period, with the number of admissions per patient ranging from 0 to 40 (median, 4 admissions [IQR, 2-7 admissions]). Overall, 1017 of 1781 admissions (57.1%) were nonelective, of which 537 (52.8%) were considered PD-related (eTable 3 in Supplement 2).

The number of nonelective admissions was similar in the dopamine agonist group and the combined MAO-B and COMT groups (mean [SD] per patient, 2.25 [2.99] admissions vs 2.34 [2.71] admissions; *P* = .96), as was admission duration (mean [SD], 14.2 [20.9] days vs 14.3 [21.2] days; *P* = .98). Fewer nonelective admissions per patient occurred in the MAO-B group vs the COMT group (mean [SD], 1.99 [2.38] admissions vs 2.29 [2.79] admissions; *P* = .18), and the duration of each admission was shorter in the MAO-B group vs the COMT group (mean [SD], 12.2 [18.6] days vs 14.7 [22.0] days; *P* = .14); hence, the total admission duration per patient was nonsignificantly shorter in the MAO-B group vs the COMT group (mean [SD], 24.3 [40.0] days vs 33.6 [53.9] days; *P* = .10) (eTable 5 and eFigure 4 in Supplement 2). The rates of first nonelective admissions, PD–related admissions, and fall-related admissions did not differ between groups (eTable 4, eTable 5, eFigure 3, and eFigure 4 in Supplement 2).

## Discussion

This randomized clinical trial found that, among patients with inadequately controlled PD, no advantage occurred from the addition of a dopamine agonist compared with a DRI (either an MAO-B or COMT inhibitor) with regard to the primary outcome of patient-rated mobility (as assessed by PDQ-39 score) or utility (as measured by the EQ-5D-3L). However, of the 2 DRIs, MAO-B inhibitors were superior to COMT inhibitors for both the PDQ-39 mobility score and the EQ-5D-3L utility score, with the effect size similar in extent to the 3.2-point difference now considered the minimal clinically important difference for PDQ-39 mobility scores.^18^ Dopamine agonists also outperformed COMT inhibitors by a similar margin but, perhaps because fewer participants were randomized in this comparison, the difference was not significant.

Although the differences between drug classes in our direct randomized comparisons were not highly significant, they were made more plausible because of the consistency of benefits observed across the different outcome measures. The results were also consistent with indirect comparisons between placebo-controlled clinical trials, which suggested that entacapone, the only COMT inhibitor assessed in the PD MED study, was a relatively weak adjuvant agent compared with dopamine agonists and MAO-B inhibitors with regard to off time and levodopa dose reduction.^2,3^ Indirect comparisons between placebo-controlled clinical trials have suggested that entacapone is also less effective than tolcapone, the alternative COMT inhibitor, with regard to off time and levodopa dose reduction.^2,3^ However, tolcapone was withdrawn from the market because of concerns about hepatic toxic effects, so this medication was rarely used in the PD MED clinical trial; only 7 patients switched to tolcapone at some stage of their treatment.

A full cost-utility analysis will be reported separately. However, the superiority of MAO-B inhibitors over COMT inhibitors and the approximate equivalence of MAO-B inhibitors to dopamine agonists suggests that the economic analyses are likely to favor the less expensive MAO-B inhibitors vs COMT inhibitors or dopamine agonists. The nonsignificant reductions in dementia and mortality among those who received MAO-B inhibitors compared with COMT inhibitors add support for this possibility, and no safety concerns were observed among those receiving MAO-B inhibitors. A clinical trial of MAO-B inhibitors conducted by the Parkinson Disease Research Group of the UK reported that selegiline therapy was associated with increased cardiovascular mortality,^19^ but this finding has not been replicated in the PD MED study^1^ or other clinical trials included in a meta-analysis.^20^

The benefits observed in the PD MED study were achieved despite suboptimal adherence; only an estimated 50% of participants were still receiving their randomized drug class at 5 years. Suboptimal adherence was not likely to have affected the qualitative findings; however, as the proportion of patients discontinuing their randomized treatment was similar across the different drug classes, as were the levodopa and levodopa equivalent doses. Because nonadherence typically compromises real differences between treatment groups, full adherence may have resulted in larger treatment differences. The most frequent reasons for withdrawal from randomized treatment were confusion and psychosis among those receiving dopamine agonists or MAO-B inhibitors and diarrhea among those receiving COMT inhibitors, which are all well-recognized AEs.^21^ Withdrawal from treatment was more common among older patients, suggesting a cautious approach to adjuvant therapy among that population.

### Strengths and Limitations

This study has several strengths. One strength of the PD MED study is that it is one of the first randomized clinical trials of PD to use a national hospital registry database (the UK HES) to assess safety and resource use. Patient-reported resource use is known to be subject to recall bias, with participants often underreporting or overreporting hospitalization.^22^ The HES database can provide almost complete data on hospitalization that are unbiased with respect to treatment allocation, enabling robust assessments of long-term efficacy and safety as well as cost-effectiveness. No clear differences in hospitalization rates were found between groups, although there was some suggestion of longer time in the hospital among patients in the COMT group, which is consistent with findings from the patient and clinical rating scales. The 70% rate of hospitalization in the PD MED study was high but comparable with the 68% rate reported from a 6-year study of PD admissions in Ontario, Canada.^23^ Infections, falls, fractures, injuries, and worsening motor function were the most common reasons for nonelective admissions, which is again consistent with previous reports.^24^

The performance of pragmatic real-world clinical trials such as the PD MED, which determined eligibility based on the uncertainty principle, can facilitate large-scale recruitment and ensure that a heterogeneous population of patients is recruited, producing results that are more generalizable to typical people with PD compared with clinical trials that apply more restrictive entry criteria. For example, adherence to treatment in the PD MED population is likely to reflect real-world adherence, so the results may also be more readily extrapolated to usual practice. The age distribution of patients in the PD MED study was similar to that of people with PD in the general population,^25^ in contrast to most clinical trials of PD therapy, which have recruited younger populations.^26^ Future clinical trials assessing the comparative benefits and risks of different drugs would be more informative if they similarly aimed to recruit older, hence more representative, patients because both the frequency of AEs and the duration of hospital stays increase with age. The 5-year median follow-up period is another strength of the PD MED clinical trial; most previous clinical trials of adjuvant therapy had follow-up periods of only 12 to 24 weeks.^26^ Parkinson disease is a chronic condition, so long-term follow-up is important to reliably assess the clinical benefits and cost-effectiveness of treatments.

The study also has limitations. One potential limitation of the PD MED study’s pragmatic design is that treatment was open label, so the potential for performance and reporting bias exists. Substantial bias is, however, unlikely because all patients were receiving active treatment and, if clinicians or patients had any a priori assumptions about comparative efficacy, these assumptions would likely have been counter to the results found in the PD MED study. Thus, any reporting bias might have been more likely to reduce rather than increase treatment differences.

## Conclusions

In this randomized clinical trial, no measurable improvement in patient-rated quality of life was observed between patients receiving dopamine agonists compared with DRIs, either MAO-B or COMT inhibitors, as adjuvant therapy for the treatment of later-stage PD. However, the use of either dopamine agonists or MAO-B inhibitors as initial adjuvant therapy appeared to be preferable to entacapone, which was the only COMT inhibitor assessed. The MAO-B inhibitors produced disease control that was equivalent to that of dopamine agonists, which suggests that MAO-B inhibitors might be underused as adjuvant therapy for the treatment of people with PD.

## Notes

Supplement 1.
Trial Protocol

Supplement 2. **eTable 1.** Participant Demographic and Baseline Characteristics by Randomization Strata and Protocol Comparison **eTable 2.** Adverse Events Experienced by Patients When Stopping Treatment **eTable 3.** Caregiver-Reported Quality of Life Using the SF-36 **eTable 4.** Elective and Nonelective Hospital Admissions for Any Reason and for PD-Related Reasons **eTable 5.** Nonelective Hospital Admissions **eFigure 1.** Clinical Trial Flowchart **eFigure 2.** Levodopa Dose by Allocated Treatment **eFigure 3.** Subgroup Analyses **eFigure 4.** 10-Year Risk of Nonelective Hospital Admission

Supplement 3.
Data Sharing Statement

Supplement 4. **Nonauthor Collaborators.** The PD MED Collaborative Group
