# Supplementary material for: Long-term Effectiveness of Adjuvant Treatment With Catechol-O-Methyltransferase or Monoamine Oxidase B Inhibitors Compared With Dopamine Agonists Among Patients With Parkinson Disease Uncontrolled by Levodopa Therapy: The PD MED Randomized Clinical Trial
Source: JAMA Neurol. 2021 Dec 28;79(2):1–10. doi: 10.1001/jamaneurol.2021.4736 (PMC8715387; doi:10.1001/jamaneurol.2021.4736)
Supplement: Supplement 1. — Trial Protocol [file jamaneurol-e214736-s001.pdf]

A large randomised assessment of the relative cost-effectiveness of different classes of drugs for Parkinson's disease

PROTOCOL

Parkinson's disease (PD) is one of the commonest causes of disability in older people with at least 8,000 new cases diagnosed each year in the UK alone. Levodopa (LD) controls symptoms for most patients but long-term use is associated with motor complications. A number of other drugs have been used, either alone or with reduced doses of LD, in an attempt to delay the onset of motor complications, or to control complications in later disease once they have developed. These agents have primarily been from three classes of drug: dopamine agonists (DA), monoamine oxidase type B inhibitors (MAOBI) and catechol-O-methyltransferase inhibitors (COMTI).

All of these drugs are beneficial, but there is uncertainty about their relative effectiveness. This is because previous comparative studies included too few patients, most had inadequately short follow-up, and the overall impact of the drugs on the patient's quality of life was not properly assessed. For example, DAs delay the onset of motor complications compared to LD, but this needs to be balanced against poorer control of the symptoms of PD, and worse side-effects - including nausea, hallucinations, sleep disturbance and oedema - which may be more important for patients and carers than motor complications. There are also uncertainties about the role of the potentially neuroprotective MAOBI, selegiline, partly because the UK PDRG trial closed early when an increase in mortality was seen with selegiline compared to LD. However, this has not been confirmed in other studies. Similarly, the COMTI, entacapone, is becoming widely used in later disease, but its efficacy compared to alternative drugs is uncertain. The new DAs and COMTIs are considerably more expensive than either LD or selegiline, and more reliable evidence is needed on the balance of benefits and risks of these drugs to establish their relative cost-effectiveness.

PD MED is a large, simple, "real-life" trial that aims to determine much more reliably which class of drug provides the most effective control, with the fewest side-effects, for both early and later PD. Patients with early PD are randomised between DA, MAOBI and LD alone, with the option to omit either the MAOBI or LD alone arm. Those whose disease is no longer controlled by their first class of drug, after dose titration and/or addition of LD, are randomised between COMTI, MAOBI and DA, with the option to omit either the MAOBI or the DA arm. The main outcome measure is the patient-rated PDQ-39 quality of life scale, which assesses all aspects of the patient's life, and is sensitive to changes considered important to patients but not identified by clinical rating scales.

Early disease randomisation

DA (± LD) MAOBI* (± LD) LD* alone

Later disease randomisation

COMTI (±LD) MAOBI* (±LD) DA* (±LD)

*optional arm

In order to recruit the large number of patients needed to provide reliable answers, and to maximise the clinical relevance of the findings, the trial is designed to fit in with routine practice as far as possible and to impose minimal additional workload: clinicians can use the specific drug within each class that they prefer, treatments are prescribed in the usual way, and extra clinic-based tests and evaluations have been kept to a minimum (the majority of assessments are by postal questionnaires to patients and carers). Because the success of the trial depends entirely on the whole-hearted collaboration of a large number of doctors, nurses and others, publication of the main results will be in the name of the collaborative group and not those of the central organisers.

PD MED is funded by the NIH Research Health Technology Assessment programme and is supported by the European Parkinson's Disease Association, the Parkinson's Disease Society and the Parkinson's Disease Nurse Specialist Association

PD MED Trial Steering Committee:

Neurology
Professor Adrian Williams (Chair)
University Hospital, Birmingham
Professor Carl Clarke
City Hospital, Birmingham
Dr Richard Greenhall,
Radcliffe Infirmary, Oxford

Health Economics
Professor Alastair Gray
Dr Emma McIntosh
Health Economics Research Centre, Oxford

Geriatrics
Dr Brian Wood
North Tyneside General Hospital

General Practice
Professor David Mant
University of Oxford

PD Nurse Representative
Linda Caie
Grampian University Hospital, Aberdeen

Quality of Life
Professor Ray Fitzpatrick
Professor Crispin Jenkinson
University of Oxford

Statistics
Natalie Rowland
Professor Richard Gray
Professor Keith Wheatley
University of Birmingham

International Representatives
Dr Irena Rektorová
St Anna's Hospital, Brno, Czech Republic
Dr Elena Chikina
Russian State Medical University, Moscow, Russia

Patient Representatives
Mary Baker MBE
President European Parkinson's Disease Association
Linda Kelly
Chief Executive, Parkinson's Disease Society

Independent Data Monitoring Committee
For interim analyses and response to specific concerns
Professor Peter Sandercock (Chair), Professor Peter Crome, Dr Colin Baigent

PD MED Trial Office
The University of Birmingham Clinical Trials Unit
University of Birmingham, Public Health Building
Edgbaston, Birmingham, B15 2TT
Telephone: 0121 415 9127/9128/9129 (Voicemail outside office hours)
Fax: 0121 415 9135
E-mail: PD-Trials@bham.ac.uk

Team Leader: Sarah Tearne
Trial Management: Jane Futterer
Data Management: Sophie Reckless

Computing
Nick Hilken

Meta-analysis
Dr Rebecca Stowe

Queries should be directed to the PD MED Trial Office staff.

FOR RANDOMISATIONS TELEPHONE
FREE PHONE 0800 953 0274 (0900-1700 UK TIME)
OR +44 (0)121 415 9129 FROM OUTSIDE THE UK
OR FAX +44 (0)121 415 9135

ISRCT No: 69812316
EudraCT No: 2005-001813-16
Sponsor: University of Birmingham
Chief Investigator: Prof Carl Clarke, University of Birmingham

CONTENTS

1. BACKGROUND 1
1.1. Parkinson's Disease 1
1.2. Treatment of early PD 1
1.3. Treatment of later PD 3
1.4. The need for PD MED, a large 'real life' trial comparing PD therapies 4
2. TRIAL DESIGN 5
2.1. Separate randomisations in early and later disease 5
2.2. Early PD randomisation 5
2.3. Later PD randomisation 6
3. LARGE, SIMPLE TRIAL: MINIMAL EXTRA WORKLOAD 7
3.1. Simple eligibility and randomisation based on "uncertainty" 7
3.2. Open label treatment 8
4. OUTCOME MEASURES 8
4.1. Patient and carer outcomes 8
4.2. Resource Usage 9
5. TRIAL PROCEDURES: RANDOMISATION 10
5.1. Eligibility 10
5.2. Patient and carer information leaflet 11
5.3. Baseline assessments 11
5.4. Randomisation 11
6. TRIAL PROCEDURES: TREATMENT AND FOLLOW-UP 11
6.1. Drug dosages 11
6.2. Treatment modifications 11
6.3. Other management at discretion of local doctors 11
6.4. Follow-up assessments 12
6.5. Serious and unexpected adverse events 12
7. SIZE OF DIFFERENCE TO BE MEASURABLE 13
7.1. Projected accrual 13
7.2. Treatment Comparisons 14
7.3. Stratification variables 14
7.4. Data Monitoring Committee: determining when clear answers have emerged 14
8. ORGANISATION 15
8.1. Principal Investigator at each centre 15
8.2. Central co-ordination: supply of all trial materials, randomisation service, data collection and analysis 15
8.3. Cost implications 15
8.4 Research Governance 16
8.5. Indemnity 16
8.6. Publication and ancillary studies 16
8.7 Archiving 16
9. REFERENCES 17
10. TRIAL SCHEMA 19

“There are nearly no data for comparisons between interventions. If choices among equivalent therapeutic options will always remain a matter of clinical expertise and individual preferences, a lot remains to be done to identify which options are equivalent. Similarly, there is a lack of data to assess the potential interest of simultaneous or successive combinations of different interventions. There are insufficient data on long-term outcomes and mortality. We hope that, by pointing out these insufficiencies, we will encourage the scientific community to do the appropriate investigations to correct such lacunas.”

Olivier Rascol, Christopher Goetz, William Koller, Werner Poewe, Cristina Sampaio
Treatment interventions for Parkinson's disease: an evidence based assessment.
Lancet 2002; **359**: 1589-98

“Substantial uncertainties about fundamental aspects of treating Parkinson's disease remain, and after decades of research into both early and later Parkinson's disease we still have little evidence on which to base decisions between different classes of drug.”

Keith Wheatley, Rebecca L Stowe, Carl E Clarke, Robert K Hills,
Adrian C Williams, Richard Gray
Evaluating drug treatments for Parkinson's disease: how good are the trials?
BMJ 2002; **324**:1508-11

“Most trials of drug treatment for Parkinson’s disease have crucial methodological faults - and provide little reliable evidence on differences between classes of drugs.”

BMJ Commentary. BMJ 2002:**324**:1508

“**More reliable evidence is needed on the balance of benefits and risks of the new DAs to establish their cost-effectiveness. Future trials should include global measures of the patient's quality of life as primary outcome measures.”**

N.J. Ives, R.L. Stowe, L. Shah, R.J. Hawker, C.E. Clarke, R.G. Gray, K. Wheatley
Meta-analysis of 5038 patients in 28 randomised trials comparing dopamine agonists with levodopa
6th International Conference AD/PD 2003 Seville, Spain May 8-12, 2003. Abstract no. 469

“……the long-term benefits of initial dopamine agonist therapy remain unproven. A very large trial currently under way in the United Kingdom (PD MED) is randomizing hundreds of subjects to initial treatment with LD/DI preparations, dopamine agonists, or selegiline. Five-year follow-up is projected. Outcomes evaluated will include quality of life, cost-effectiveness, and incidence of motor complications. Widespread changes in clinical practice should await the accumulation of more trial data.”

R.L.Albin, K. A. Frey
Initial agonist treatment of Parkinson disease. A critique
Neurology, 2003; **60(3)**: 390-394

1. BACKGROUND

1.1. Parkinson's Disease

Parkinson's disease (PD) is a progressive neurological disorder caused by the loss of pigmented dopaminergic neurones in the brain and the consequent depletion of the neurotransmitter dopamine. This leads to increasing disability due to motor complications, including tremor, rigidity, slowness, and postural disturbance. PD is one of the commonest causes of disability in older people. It is estimated that at least 8,000 new cases of PD are diagnosed in the U.K. each year. Average life expectancy is about 15 years, leading to a prevalence of over 100,000 cases and incidence increases rapidly with age, 95% of patients are aged over 40 years at diagnosis, with most patients developing the initial symptoms of PD between 50 and 70 years of age. There is currently no curative therapy for PD, so treatment is directed towards the alleviation of symptoms.[1]

1.2. Treatment of early PD

Levodopa (LD) provides good symptomatic relief for most patients with PD and may improve their survival.[2, 3] However, after a few years of treatment, motor complications ("wearing-off", "on-off" fluctuations and dyskinesia) often develop. It is unclear to what extent these complications are due to disease progression or to cumulative LD effects. Dopamine agonists (DAs) and monoamine oxidase type B inhibitors (MAOBIs) have been used, either alone or with reduced doses of LD, in an attempt to delay the onset of motor fluctuations. A systematic review of the existing randomised evidence confirms the increased risk of motor complications with LD, but also indicates that disease control is not as good and other side-effects are increased with DAs. The available evidence from randomised trials assessing the various therapeutic options in PD, published prior to January 2003, is shown below (see Table 1). As outcome data are inconsistently reported, an informal non-quantitative scoring system is used to indicate outcome.

Table 1 : Summary of results of randomised trials of dopamine agonists and dopamine degradation inhibitors (MAOBIs and COMTIs) in early Parkinson's disease

Comparison	No. of trials (patients)	Mean follow-up (years)	Clinical disability scales	Motor Complications	Other side effects	LD Dose Reductions
Levodopa v placebo[3]	1 (361)	0.8	++	++	-	n/a
DA v placebo (± LD)	11 (1308)	0.9	++	No data	- -	+
MAOBI v placebo (± LD)	13 (1485)	3.5	+	o	(-)	+
COMTI v placebo (± LD)	2 (381)	0.8	(+)	(+)	(-)	+
DA (± LD) v LD	21 (4393)	4.6	-	++	- -	+
MAOBI (± LD) v LD	2 (852)	7.2	o	(+)	o	No data
MAOBI v DA (± LD)	1 (335)	2.8	o	o	No data	(-)
Other[$]	7 (1091)	6.2				
**TOTAL**	**46 (8072)**	**3.7**				

(+) = possible benefit; + = small benefit; ++ = moderate benefit; o = no difference; (-) = possible adverse affect; - = small adverse affect; - - = moderate adverse effect (scores indicate benefit, or harm, from left hand comparator)

Patients in 5 trials with multiple comparisons count towards more than one comparison but just once to the total, which give the actual numbers of trials, patients randomised and follow-up lengths.

[$] Other early disease comparisons included DA v DA (±LD), DA (+MAOBI) v LD, DA (+MAOBI) v DA, DA (+MAOBI) v MAOBI (+LD).

Abbreviations: LD = levodopa; DA = dopamine agonist; MAOBI = monoamine oxidase type B inhibitor; COMTI = catechol-O-methyltransferase inhibitor

For detailed information on trials included in the table, see references 3 & 8.

Dopamine agonists: Dopamine agonists (DAs) are widely used as add-on therapy in later disease, and are now being increasingly used in first-line treatment, particularly for younger patients. Trials comparing DA with placebo in early disease have generally reported improved outcome with DA, usually with respect to one of a number of clinician-scored impairment/disability scales, most commonly the Unified Parkinson's Disease Rating Scale (UPDRS). Trials comparing DA (with LD introduced as necessary) with LD alone have established benefits for DAs in terms of delaying the onset of motor complications, with reductions in dyskinesia and in the dose of LD required.[4-7] However, symptomatic control is inferior with DAs and some side-effects, including nausea, oedema, hallucinations, constipation, dizziness and sleep disturbance, are increased by DAs. As previous trials have failed to assess the overall impact of the drugs on the patients' quality of life, it is unclear whether DAs are superior to LD, from the patients' perspective. The newer DAs (pergolide, ropinirole, cabergoline and pramipexole) are considerably more expensive than LD, or the older DA, bromocriptine, and their relative cost-effectiveness needs to be more reliably assessed. Very few trials have directly compared one new DA with another and hence there is no reliable evidence on whether particular DAs are better than others.[8,9]

MAOB inhibitors: Inhibitors of dopamine degradation, such as the monoamine oxidase type B inhibitors (MAOBIs), are a second class of drugs that has been widely used as LD-sparing therapy in early PD, or as an add-on to LD in later PD. Trials of selegiline, the most frequently used MAOBI, versus control, with or without LD in both arms, in early PD have consistently shown improvements in the UPDRS and other disability scales with selegiline. These trials have also demonstrated that selegiline treated patients can be maintained on lower doses of LD, and provide some support for a neuroprotective effect of selegiline. However, the largest trial of selegiline plus LD versus LD alone was halted prematurely because of increased mortality in the selegiline arm compared to LD, raising concerns about its use. [10,11] Subsequent data, including later follow up from the initial adverse study, show no increase in deaths with selegiline[12-15] and a meta-analysis of all trials confirms this.[16] In total, 283 (24%) deaths have occurred among 1181 selegiline allocated patients and 255 (22%) among 1149 control patients, a non-significant difference (see below).

Meta–analysis of deaths in trials of selgiline

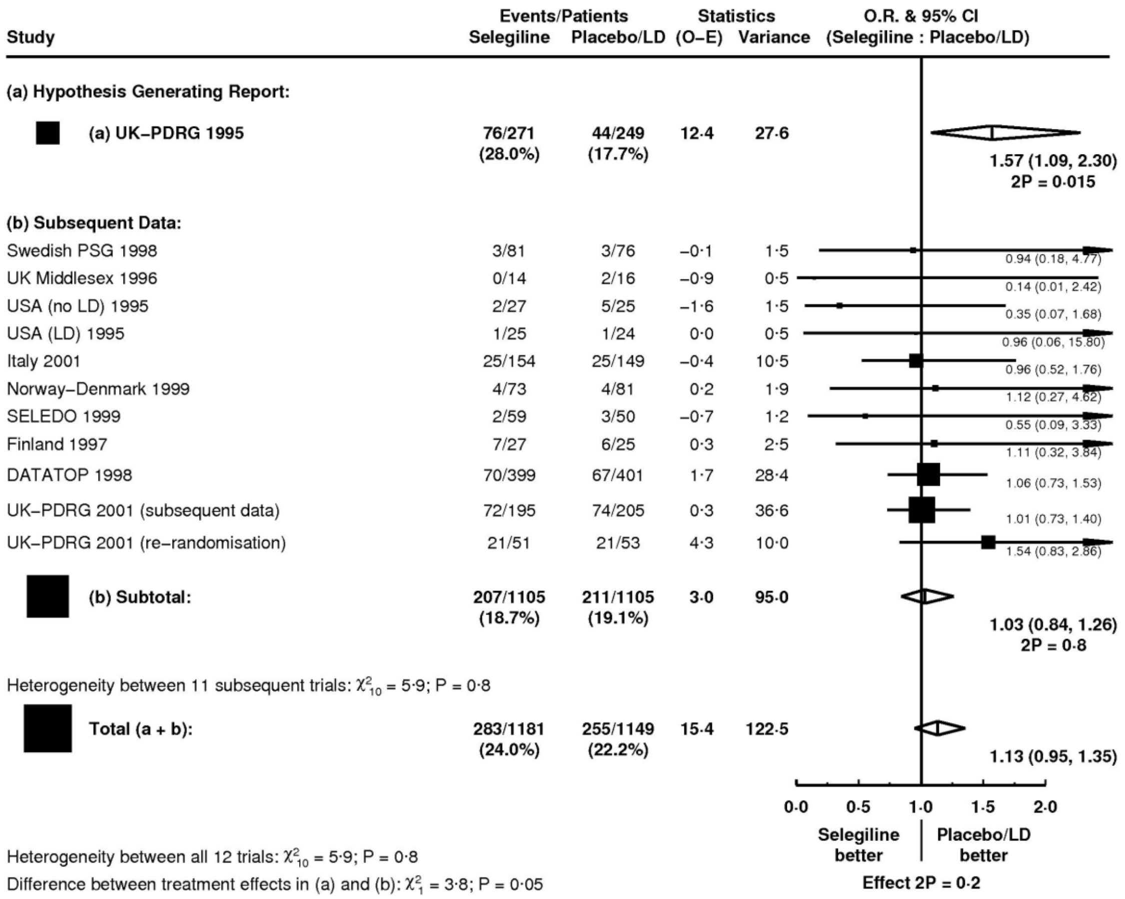

It seems highly likely, therefore, that the excess deaths with selegiline in the UK-PDRG trial, which was of only borderline statistical significance, was a chance finding. Nonetheless, this "scare" has had the effect of reducing the use of selegiline, an inexpensive drug which may be the most cost-effective drug available for treatment of early PD. This reassuring finding, plus the recent licensing of a new MAOBI, rasagiline, has revived interest in MAOBIs as an alternative LD-sparing therapy. There is, unfortunately, only one trial comparing MAOBIs with DAs in early PD and hence no good evidence on the relative effectiveness of MAOBIs compared to the more expensive DAs is available. A sub-lingual form of selegiline is also available but, again, this has not been properly evaluated in large randomised trials.

1.3. Treatment of later PD

Once dyskinesia, "wearing-off" and "on-off" fluctuations develop with LD monotherapy, it is unclear which drugs should be introduced. DAs and dopamine degradation inhibitors (DDIs), such as MAOBIs and the newer catechol-O-methyltransferase inhibitors (COMTIs), are commonly used. However, there is even greater uncertainty as to the relative value of these alternatives as even fewer patients with later PD have been entered into randomised comparative trials than with early disease. Most trials have compared LD plus another drug with LD alone with the largest body of evidence on the role of DAs. Almost all trials have been short-term with a mean duration of less than 6 months. Table 2 summarises the results in an informal non-quantitive fashion, as with Table 1.

Table 2 : Summary of results of randomised trials of dopamine agonists and dopamine degradation inhibitors (MAOBIs and COMTIs) in later Parkinson's disease

Comparison#	No. of trials (patients)	Mean follow-up (years)	Clinical disability scales	Other side effects	LD dose reductions
DA v placebo	23 (2231)	0.5	++	(-)	+
MAOBI v placebo	17 (498)	0.4	++	(-)	+
COMTI v placebo	16 (2166)	0.5	++	(-)	+
DA v DA	9 (1265)	0.5	N/A	N/A	N/A
COMTI v DA	3 (499)	0.2	o	o	o
TOTAL	68 (6472)	0.5			

See Table 1 footnote

Patients in one multiple comparison trial count towards more than one comparison but just once to the total, which give the actual numbers of trials, patients randomised and follow-up lengths.

#All trials in later PD are on a background of LD-based therapy.
Abbreviations: PD = Parkinson's disease; LD = levodopa; DA = dopamine agonist; MAOBI = monoamine oxidase type B inhibitor; COMTI = catechol-O-methyltransferase inhibitor

For detailed information on trials included in the table, see reference 8.

Dopamine agonists: Trials of DAs versus placebo in later PD have shown improvements in UPDRS scores with DAs and some reductions in “off” time. Patients receiving DA also required slightly lower doses of LD but side-effects appeared higher with DA. Few trials have directly compared one DA with another, although advantages for the newer DAs over bromocriptine have been suggested. For example, a Cochrane review of three trials of pergolide versus the older DA, bromocriptine, has reported a moderate benefit for pergolide on clinician-based rating scales.[17]There have been no trials comparing one new DA with another.

MAOB inhibitors: Eighteen trials of selegiline versus placebo in later PD have been undertaken, but the majority of these have been very small (with an average trial size of 30 patients) and have used cross-over designs aimed at evaluating short-term endpoints. The trials indicate a moderate benefit for selegiline with respect to "off" time and clinical disability scales. However, remarkably, only one trial[17] comparing MAOBI with either DA or COMTI has been undertaken in later PD. Comparative trials, including larger numbers, more clinically relevant endpoints, and longer term outcome assessments are needed.

COMT inhibitors: COMTI's are another class of dopamine degradation inhibitors. Trials of the COMTI's, tolcapone and entacapone, compared to placebo in later PD have shown improvements in "on" time, clinician-rated disability scales, and reduction in LD dose. Tolcapone was, however, withdrawn in Europe following three fatal cases of fulminant hepatitis in about 60,000 treated patients. Although tolcapone can cause liver toxicity, it is not certain that these fatalities were caused by tolcapone, and it remains possible that the potential benefits of tolcapone in later PD might out-weigh the risks. The newer COMTI, entacapone, has also shown some evidence of improvements in disability scales, and appears not to be hepatotoxic. Although tolcapone is now available again, it is recommended that COMTI treatment should commence with entacapone with tolcapone used only if an inadequate response to entacapone. Again, the efficacy of COMTIs compared to other classes of drug has not been properly assessed, with only three trials of tolcapone versus DAs having been identified, one trial of rasagiline versus entacapone, and no trials of COMTI versus the much less expensive DDI, selegiline.

1.4. The need for PD MED, a large 'real life' between-class comparison of PD therapies

Previous trials have, on the whole, been too small to evaluate reliably moderate differences between different classes of drug: even the largest trial accrued only 800 patients, and most recruited less than 50.8 Furthermore, the majority of trials have concentrated on short-term efficacy (many trials have used cross-over designs) and used physician-rated disability assessments as sole outcome measures. It is essential, in a slowly progressive disease such as PD, to evaluate the long-term effectiveness of treatment, based on clinically and socially important outcomes, and to assess the patients' perception of benefit as well as that of clinicians. Cochrane reviews are addressing a number of comparisons in PD treatment, but these are limited by serious methodological problems with some of the contributing trials (e.g. "major methodological problems preclude a conclusion on the efficacy of bromocriptine ",[18] "studies can be criticised for inadequate data on concealment of allocation, variable reporting of data on a per protocol or intention-to-treat basis and their short duration"[19]).

There is, therefore, an urgent need for much more reliable evidence on the balance of risks and benefits of LD-sparing therapy compared to LD alone in early disease, and on whether LD-sparing therapy with a DA or an MAOBI is preferable. The PD MED trial addresses this fundamental question, comparing DA versus MAOBI versus LD alone (with LD being added into the first two arms as necessary). Similarly, few data are available on the comparative efficacy of COMTIs, MAOBIs and DAs in later PD, and this question is also addressed in PD MED by a randomisation between DDI and DA, with a sub-randomisation between types of DDI (i.e. COMTI versus MAOBI), which will provide important new information on the relative efficacy of these different classes of drug.

PD MED does not directly compare different agents within particular classes of drug as this would require an impracticably large sample size. For example, there are five different DAs currently available and no good evidence that any particular DA is better than any other in either early or later disease. A randomisation between all five would require a very large sample size to allow for multiple comparisons and the consequent risk of false positive results. Choosing just two DAs (or three, at most) to compare would require an arbitrary choice and would decrease the flexibility of the trial should new evidence emerge during its course to suggest that any one particular DA may be of greater or lesser benefit. Inflexibility in choice of DA could also limit recruitment. Instead, there-fore, the choice of which DA to use in PD MED is left to the individual clinician. The DA will need to be specified at the time of randomisation, which will allow indirect comparisons of the efficacy of the various DAs, although such comparisons are statistically weak and will be used only in an exploratory hypothesis-generating sense.

As with DAs, the choice of MAOBI will be left to the individual clinician. Selegiline has been the most widely used and is also available in a sub-lingual formulation and a new MAOBI, rasagiline, was licensed in May 2005. Any of these may be selected, and the analyses will again be stratified by the MAOBI chosen, allowing indirect comparisons. Similarly, either entacapone or tolcapone (if inadequate response to entacapone) may be used as the COMTI. The withdrawal of licensing approval for tolcapone re-inforces the advisability of this pragmatic approach of allowing clinicians freedom of choice within a particular class of drug.

Thus, the first priority in PD MED is to answer reliably the fundamental qualitative question of which class of agents provides the most effective control of symptoms with the fewest side-effects. The quantitative questions of whether particular drugs within a class are more effective than others, or whether combinations of different classes of drugs are more effective than one class alone, will be questions for future trials.

2. TRIAL DESIGN

2.1. Separate randomisations in early and later disease

PD MED is a large, simple, "real-life", randomised assessment of the relative cost-effectiveness of different classes of drugs for both early and later PD.

The four fundamental questions being addressed by two semi-factorial randomisations in this trial are:

1. Does early treatment with levodopa-sparing therapy (either a DA or a MAOBI) delay deterioration in quality of life compared to LD alone?
2. Which class of LD-sparing treatment is preferable (DA or MAOBI)?
3. For patients with motor complications uncontrolled by LD alone, should DDIs or DAs be added to LD?
4. If so, which class of DDI (COMTI or MAOBI) is preferable?

2.2. Early PD randomisation

Patients recently diagnosed with PD are eligible for the early PD randomisation if:

1. They are previously untreated for PD and therapeutic intervention is considered appropriate. Patients not thought to require dopaminergic treatment at diagnosis are eligible once it is considered that such treatment becomes necessary.
or
2. They have previously been treated with dopaminergic medication, but for less than 6 months, and there is now uncertainty as to which class of drug to use. This randomisation may entail stopping, or modifying the previous therapy. This will be left to the discretion of the investigator.

Early disease randomisation

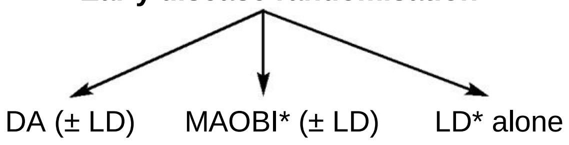

*optional arm

Question 1 will be addressed by comparison of arms 1 & 2 (DA or MAOBI) with arm 3 (LD alone).

Question 2 will be addressed by comparison of arm 1 (DA) with arm 2 (MAOBI).

If treatment with either MAOBI or LD alone is considered to be **definitely** inappropriate for a particular patient, then this arm can be omitted. For example, some clinicians may consider that for particular types of patients (e.g. younger ones) LD alone is not appropriate and, in this circumstance, a two-way randomisation between DA and MAOBI may be performed. Similarly, if a clinician considers that a MAOBI is not appropriate, a patient may be randomised two-ways between DA and LD alone. Definite indications for, or definite contraindications against, any of the therapies in the trial are not specified by the protocol, but by the responsible clinician (see Section 3.1).

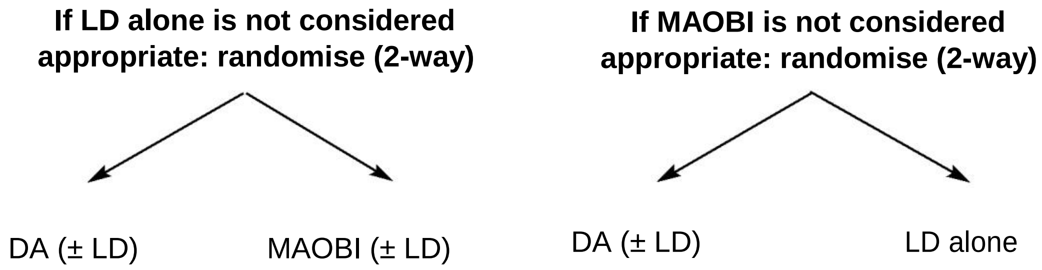

2.3. Later PD randomisation

Patients who develop motor complications that are uncontrolled by LD (alone or in combination with either DA or MAOBI), and hence require the addition of another class of drug are eligible for the later disease randomisation.

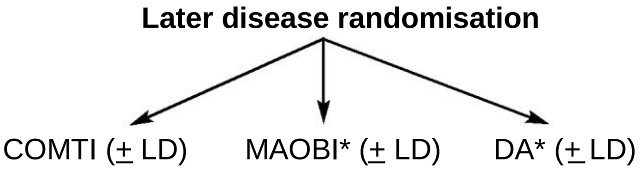

*optional arm

Question 3 will be addressed by comparison of arms 1 and 2 (COMTI or MAOBI) with arm 3 (DA).

Question 4 will be addressed by comparison of arm 1 (COMTI) and arm 2 (MAOBI).

Patients who were already receiving a DA when uncontrolled motor complications arose are not eligible for the DA arm but can be randomised between COMTI and MAOBI. Patients who were receiving a MAOBI when uncontrolled motor complications arose, or for whom the clinician considers that MAOBI treatment is definitely contraindicated, are not eligible for the MAOBI arm but can be randomised between COMTI and DA.

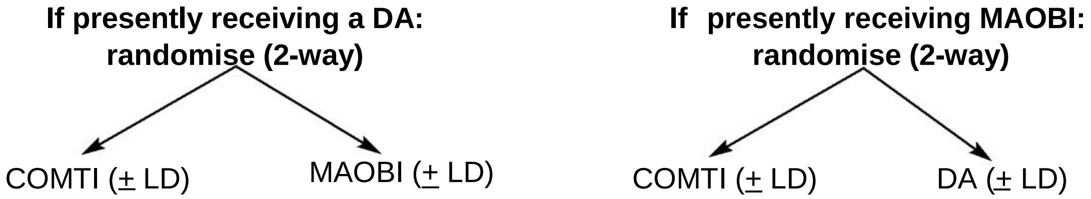

N.B. Patients who have been entered into the early disease randomisation should be re-randomised into the later disease randomisation if motor complications develop that cannot be controlled by drug dose titration and/or addition of LD if on DA/MAOBI.

3. LARGE, SIMPLE TRIAL: MINIMAL EXTRA WORKLOAD

The differences between active agents are likely to be smaller than those between any one agent and a placebo control. Thus, larger numbers of patients will be required for the reliable detection, or reliable refutation, of any worthwhile differences between different classes of drugs. To make widespread participation from a large number of centres practicable, the PD MED trial procedures are 'streamlined', with minimal extra workload placed on participating clinicians, beyond that required to treat their patients. This is achieved by simple entry procedures (a single phone/fax call to the randomisation office), the use of standard, open-label treatment regimens, follow-up as in routine practice (with no additional hospital visits or tests to be performed above those done as part of standard care), minimising documentation, and largely patient-based evaluation of outcome (through postal questionnaires). This information will be supplemented by the use of national mortality records to ensure long-term follow-up. Regular newsletters will keep participants informed of trial progress, and regular meetings of collaborators will be held to address any problems encountered in the conduct of the study.

3.1. Simple eligibility and randomisation based on "uncertainty"

There is disagreement on the extent to which the development of motor fluctuations and dyskinesia after long-term LD therapy is due to cumulative effects of LD or to progressive disease. LD-sparing therapy does appear to delay the onset of dyskinesia, but this needs to be weighed against the poorer symptomatic control, and an increase in other troublesome side-effects such as hallucinations. Because of the lack of reliable randomised evidence on which initial therapy is best, there is considerable divergence in clinical opinion and practice. At one end of the spectrum, some clinicians consider that the evidence for LD-sparing therapy is insufficient to justify use of the more expensive and clinically less effective new DAs. Such doctors, who are sceptical about LD toxicity, might consider using LD-sparing therapy only for younger patients for whom the potential for long-term toxicity is a more important consideration. Other clinicians believe that younger patients should be offered LD-sparing therapy on existing evidence but are uncertain whether more elderly patients should be offered LD or LD-sparing therapy. Still others would wish to consider either LD or LD-sparing therapy for their whole range of patients. Other factors, such as the level of disability of patients, are also potential determinants of the appropriateness of different Parkinson's treatment - and, again, there are divergent opinions. Similarly, some doctors are concerned about the safety of selegiline because of the UKPDRG Study and would wish to avoid using it. Others are sceptical about this evidence and believe that this inexpensive drug of proven effectiveness should not be discarded prematurely.

In view of these considerations, the PD MED trial adopts a pragmatic approach with eligibility based not on rigid entry criteria but on the "**uncertainty principle**". That is, if the doctor considers, for any reason, that there is a **definite indication** for, or a **definite contraindication** against, a particular class of PD drug, then the patient is not eligible for a randomisation including this class of drug (although the patient can still be randomised in one of the two-way randomisation options). If, on the other hand, the doctor is **substantially uncertain** which class of drug a particular patient should be offered, that patient is eligible to be randomised. In these circumstances randomisation is both scientifically and ethically preferable to the uninformative alternative of not randomising and treating patients in an ad hoc way outside of a study. Eligibility based on uncertainty has been used in many previous trials (e.g. the "ISIS" trials in acute MI, the MRC carotid endarcterectomy trial, and the QUASAR colorectal cancer trial) and has been shown to simplify trial procedures and to facilitate large-scale recruitment of an appropriately heterogeneous group of patients.[20]

3.2. Open label treatment

Blinding of treatment allocation is not considered necessary in PD MED because the potential for subjectively biased assessment is small. There is no reason to expect that patients will have any prior beliefs that one treatment will be better than another (all patients in both randomisations receive active therapy - there are no placebo arms). Likewise the main outcome measures are well-validated, reproducible, patient-rated measures of disability and quality of life.

Moreover, the pragmatic, ‘real life’ design of the trial, which allows clinicians to choose which DA, MAOBI and COMTI to use, and to vary the dose as they see fit, has substantial advantages. The eventual results will be more clinically relevant, in that drug usage will reflect normal clinical

practice which involves frequent dose adjustments to achieve optimal symptom control. Another factor that precludes blinding is the cost of buying, encapsulating and distributing all the drugs for this long-term study, which would be prohibitive. Furthermore, patients with PD will normally obtain their prescriptions from their GPs. Trial procedures are simplified, treatments are given as they would be in normal clinical practice and administrative costs are greatly reduced with open treatment. The substantial advantages of simple, 'real life' procedures that will facilitate large-scale recruitment from many centres, enabling a uniquely large and therefore a uniquely reliable evaluation of the relative merits of different drugs to be undertaken, greatly outweigh the small possibility of assessment bias with open-label treatment.

4. OUTCOME MEASURES

4.1. Patient and carer outcomes

The primary outcomes will be the patient's self-evaluation of their functional status and quality of life (using the PDQ-39 questionnaire) and cost-effectiveness (EuroQoL EQ-5D).

Secondary endpoints will evaluate other aspects of functionality, and safety:

- Cognitive function (MMSE)
- Well being of carers (SF-36)
- Resource usage
- Toxicity and side-effects, including mortality rates
- Time to onset of motor complications (early disease randomisation only) and time to surgical intervention or start of apomorphine (later disease randomisation only)
- Capability in older people used in economic evaluation (ICECAP-O)
- Carer Experience Scale (CES)

PDQ-39: A clinically and socially meaningful outcome measure needs to address matters of most concern to the individual with PD. The PDQ-39 is a patient-completed questionnaire developed by qualitative in-depth interviews involving patients with PD. It includes items that reflect patients' concerns in relation to eight aspects of PD: mobility, activities of daily living, emotional well-being, stigma, social support, cognition, communication and bodily discomfort.[21,22] The instrument has been extensively tested for validity, reproducibility and sensitivity to change in both clinic and population survey applications. For example, the instrument has high convergent and discriminant validity in relation to neurologists' assessments of PD severity using conventional clinical scores, such as Hoehn and Yahr, Columbia and UPDRS, and is sensitive to changes considered of importance to patients, but not identified by clinical ratings.[23,24] It has been translated and used in most European, Australasian and North American countries and has been widely used as an outcome measure in trials of drugs, neurosurgery and nursing care packages.[25]

EuroQoL EQ-5D: The main outcome measure for the economic evaluation will be the EuroQol EQ-5D. Responses will be given valuations derived from published UK population tariffs and the mean number of quality adjusted life-years (QALYs) per patient and incremental QALYs will be calculated. The incremental cost per QALY will then be calculated. All parameters subject to uncertainty will be systematically varied in sensitivity analyses.

ICECAP-O: is a new measure of capability in older people for use in economic evaluation. Unlike most profile measures used in economic evaluations, the ICECAP-O focuses on wellbeing defined in a broader sense, rather than health. The measure covers attributes of wellbeing that were found to be important to older people in the UK. It will be evaluated to see if it provides an improved sensitivity in outcome measures for patients with PD compared to the EQ-5D.

Cognitive function - Mini-Mental State Examination (MMSE): About 10% of PD patients develop dementia. The trial aims to determine whether therapies prevent or decrease the decline of cognitive function - as measured by MMSE - in PD. MMSE is a well-established 30-point

measure of cognitive function in older people. It is easy to administer, shows good test/retest and inter-rater reliability and performs satisfactorily against more detailed measures of cognitive function. The MMSE is more sensitive than alternative measures at milder levels of cognitive impairment. MMSE score is influenced by sociodemographic status but this will even out in a large randomised study. Levels of 10 to 26 correspond to mild to moderate cognitive impairment in dementia. A score below 10 represents severe disabling dementia and is a milestone from which patients rarely recover.

Carers
The primary carer should be in at least weekly contact with the patient, preferably co-resident, and should not be someone who is employed as a carer. If there is no suitable carer, or the carer chooses not to participate, the patient can still take part in PD MED.

Carers' psychological well-being: Little work has been done on the effect of anti-Parkinsonian drug prescription on carer attitudes, stress or physical and psychological morbidity. The person identified by the patient as their primary carer will be assessed by the SF-36, a well-validated measure of health status.

Carer Experience Scale: It is a new profile measure of the caring experience for use in economic evaluation. Unlike most profile measures used in economic evaluations, the CES focuses on 'care-related quality of life' rather than health-related quality of life, comprising attributes that are pertinent to unpaid carers.

Side-effects of treatment: The PDQ-39 includes items to assess self-rated severity of PD symptoms. In addition, potential side-effects of drugs, changes to drugs and institutional stays will be assessed by a patient-based instrument developed specifically for the study.

4.2. Resource usage

Direct medical costs: An economic evaluation will be undertaken as part of the trial. Depending on the clinical results of the study, a cost minimisation study (no clinical difference between therapies) or cost-utility analysis (cost per incremental QALY gained) will be performed. Data will be collected from a sample of patients on the volume and type of resources used over the follow-up period. Information on medications, clinic visits, adverse events, hospitalisations and institutionalisation will be collected as an integrated part of the trial case record forms. A sub-sample of patients will be used to estimate the volume and opportunity costs of formal and informal care received by patients. Further details of hospitalisations (main reasons for admission, length of stay) will be collected from the relevant hospitals as required. In addition, patients will be asked at 12-month intervals to complete a simple (one A4 sheet) postal questionnaire covering GP consultations, physiotherapy out-patient visits, hospital stays and other health care resources used over the previous 12 months. All resources used will be costed using current unit costs derived from national statistics and participating centres, and a mean net cost per patient in each trial arm and incremental cost per patient with associated measures of variance will be calculated.

Institutional care: Progression of PD may lead to increased requirements for formal domiciliary or residential care as the limits of informal care are exceeded in some patients. Transitions to more intensive forms of care can be viewed both as outcome and as costs. The transitions to formal or paid inputs of care will impose costs either on the public sector or families. Public sector costs are likely to be borne initially by the NHS in terms of short term admissions (geriatrics, neurology), followed by individual needs assessment by the Local Authority Social Services (LASS), leading in turn to packages of domiciliary care and later, if and as appropriate, to placement in a residential care or nursing home. To the extent that PD therapies delay these transitions, they may reduce costs. The economic evaluation will adopt a societal perspective including informal and formal costs, i.e. those borne by the NHS and by LASS or privately by patients or their families.

5. TRIAL PROCEDURES: RANDOMISATION

5.1. Eligibility

Eligibility will be based on the uncertainty principle (see Section 3.1). Patients will be eligible if they have a confirmed diagnosis of Parkinson's disease, either early disease (newly/recently diagnosed) or later disease (motor complications).

Early disease randomisation:

Patients are eligible for the early disease randomisation if:

- They are newly or recently diagnosed with Parkinson's disease. It is important to ensure the accurate diagnosis of PD and the UK Brain Bank criteria should be used.
- They have functional disability requiring medical therapy. Patients not thought to require dopaminergic treatment at diagnosis may be entered once it is considered that such treatment becomes necessary.
- They are previously untreated for PD or have been treated with dopaminergic PD medication for less than 6 months.
- There is no definite contraindication to, or definite indication for, any of the therapies to which they might be allocated. (If it is considered that LD only is not an appropriate option for a patient, they may be randomised two ways between DA and MAOBI. Similarly, if a MAOBI is not considered appropriate, a patient may be randomised two ways between LD and DA.)
- They are able to complete the trial questionnaires. Non-English speaking patients may be entered if they have a carer, relative or other person who can help them fill in the questionnaires, or if translated documentation is available.

Patients are not eligible for the early disease randomisation if:

- They have received previous dopaminergic drug therapy for PD for more than 6 months.
- They are demented (as defined by the medical team responsible).
- They are unable to give informed consent.

Later disease randomisation:

Patients are eligible for the later disease randomisation if:

- They have PD and develop motor complications that are uncontrolled by LD (either alone or in combination with either a DA or a MAOBI) and hence require the addition of another class of drug.
- There is no definite contraindication to, or definite indication for, any of the therapies to which they might be allocated. (Patients who were already receiving a DA when uncontrolled motor fluctuations arose are not eligible for the DA arm and will be randomised between MAOBI and COMTI only. Patients who were receiving a MAOBI when uncontrolled motor fluctuations arose, or for whom the clinician does not wish a MAOBI to be an option, are not eligible for the MAOBI arm and will be randomised between DA and COMTI only.)
- They are able to complete the trial questionnaires. Non-English speaking patients may be entered if they have a carer, relative or other person who can help them fill in the questionnaires, or if translated documentation is available.

Patients are not eligible for the later disease randomisation if:

- They are demented (as defined by the medical team responsible).
- They are unable to give informed consent.

N.B. Patients who have been entered into the early disease randomisation should be re-randomised into the later disease randomisation if motor complications develop that are uncontrolled by drug-dose titration and/or addition of LD if on DA/MAOBI.

5.2. Patient and carer information leaflet

The conduct of the trial will be in accordance with the Medical Research Council policy on ethical considerations. The patient's consent (according to usual local practice) to participate in the trial must be obtained before randomisation and after a full explanation has been given of the treatment options and the manner of treatment allocation. Patient and carer information sheets and consent form will be provided so that patients and their carers can find out more about the trial before deciding whether or not to participate.

5.3. Baseline assessments

Once the patient has consented to take part, the MMSE should be administered. The patient should be asked to complete the PDQ-39, and EuroQol EQ-5D. The carer, if taking part, should be asked to complete the SF-36.

5.4. Randomisation

Randomisation notepads should be used to collate the necessary information prior to randomisation. Complete the baseline assessments as specified in Table 3 overleaf. The person randomising will need to answer all of the questions before a treatment allocation is given. Patients are entered and randomised into the trial by one telephone call to the randomisation service (0800 953 0274 free phone from within the UK or +44 121 415 9127/9128/9129 from outside the UK) or by fax (0121 415 9135 or +44 (0)121 415 9135, from outside the UK). Telephone randomisations are available Monday-Friday, 09:00-17:00 UK time. The patient's GP will need to be notified, and a “specimen letter to GP” is supplied.

6. TRIAL PROCEDURES: TREATMENT AND FOLLOW-UP

6.1. Drug dosages

The pragmatic design of the trial allows clinicians to start treatment with whichever drug they prefer as long as it is within the class of drug (i.e. LD, DA, MAOBI or COMTI) to which the patient was allocated at randomisation. Clinicians can give the chosen drug at the dose and scheduling that they normally use and can titrate the dose as they see fit in the best interests of the patient. Drug dosage information is provided and clinicians are referred to the Summary of Product Characteristics (SPC) for each drug for further details.

6.2. Treatment modifications

If disease symptoms are not adequately controlled by the class of drug allocated, after titrating the dose to the maximum tolerated, then it is permissible, as in usual practice, to add a new agent from another class of drugs. In particular, for patients with early disease allocated to a dopamine agonist or MAOBI, levodopa can be introduced as required. Investigators are encouraged to re-randomise patients whose disease is no longer controlled by the class of drug allocated, even with the addition of levodopa, into the later disease randomisation.

Treatment modifications are also permissible if patients are believed to be experiencing adverse effects from a particular drug. A different drug within the same class is preferable - for example, trying a different dopamine agonist - but an agent from a different class of drug can also be used if considered to be in the patient's best interests. Treatment modifications, and the reason for modification, should be recorded on the follow-up forms.

N.B. For purposes of follow-up and analyses, patients remain in the PD MED study irrespective of treatment compliance. It is important that questionnaires and study documentation are completed for all patients randomised so that unbiased 'intention-to-treat' analyses can be undertaken.

6.3. Other management at discretion of local doctors

Apart from giving out the trial treatments, all other aspects of patient management are entirely at the discretion of the local doctors. Patients are managed in whatever way appears best for them, with no special treatments, no special investigations, and no extra follow-up visits.

6.4. Follow-up assessments

The principal evaluations will be by means of postal questionnaires to be completed by patients and their carers. These patient-based outcome measures (PDQ-39 and EQ-5D) will be collected at base-line, six months, one year and yearly thereafter (see table 3 below). In addition patients' reports of side-effects will be collected at six months, one year and yearly thereafter and resource use data will be collected at one year and yearly thereafter. The trial follow-up involves minimal administration and paperwork on the part of clinicians and their staff. There is just a simple annual questionnaire to clinicians to ascertain changes in disease status (e.g. onset of motor complications) and changes in therapy. MMSE is measured at baseline and at every subsequent 5 years.

6.5. Serious and unexpected adverse events

Treatment-related toxicity with the drugs and dosages employed in the trial is expected to be minor. For potential toxicities, refer to the Study Product Characteristics for further details. However, to monitor the safety of the drugs used in PD MED, all serious, unexpected adverse events (see footnote A) believed to be due to the PD treatments should be reported to the Trial Office within 48 hours by telephone, e-mail or fax. A detailed report of the event on the Serious Adverse Event Form should be returned to the Trial Office within 7 days. Adverse events that might reasonably be expected to occur in PD patients receiving the trial treatments do not need to be reported in this way but should be recorded on the annual review form, when this form becomes due.

Dementia

If the patient becomes demented (as defined by the medical team responsible for the patient) then as much data should be collected as practical during the follow-up period using the EQ-5D, Resource Usage Form and an adapted version of the PDQ-39, called the PDQ-17, which may be completed by the carer. These forms will be sent directly to the patient and carer by the Trial Office. Clinical follow-up information will continue to be obtained from the patient's current doctors.

Deaths

A Serious Adverse Event form and an Annual Follow-up form should be completed and returned within two weeks if a patient dies. This information will be supplemented by use of national mortality statistics to monitor long-term survival.

Table 3 - Baseline & Follow-up assessments

Assessment	Outcome Measure	Completed by	At Entry	6 Months	1* Year	2* Years	3* Years	4* Years	5* Years
Functional Status/Quality of Life	PDQ-39, EQ-5D, ICECAP-O	Patient	✓	✓	✓	✓	✓	✓	✓
Side effects	Side effect form	Patient		✓	✓	✓	✓	✓	✓
Health Economics	Resource usage	Patient			✓	✓	✓	✓	✓
Carer well-being	SF-36, CES	Carer	✓	✓	✓	✓	✓	✓	✓
Cognitive function	MMSE	Clinician	✓						✓
Disease status	Follow up form	Clinician	Rand. notepad		✓	✓	✓	✓	✓

*Assessment schedule years 1 – 5 repeated for all participants until end of trial December 2019 (ie at least 10 years).

Footnote A "Unexpected" adverse events are defined as those that would not be expected among elderly patients given anti-parkinsonian medication (which has certain expected side-effects) for Parkinson's disease (which has expected symptoms). For the purposes of this study, **"serious"** adverse events are those which are fatal, life-threatening, disabling or require hospitalisation.

7. SIZE OF DIFFERENCE TO BE MEASURABLE

7.1. Projected accrual

The PD MED study adopts a pragmatic approach to recruitment aiming to include, if possible, 1500 patients in the early disease randomisation and 1000 in the later disease randomisation. These numbers would give very high statistical power (i.e. over 90% power at $p<0.01$) to confirm, or refute, even small differences between the different classes of drugs and, should differences emerge, would also be enough to allow meaningful exploration of any differences in the size of benefit between different types of patient, between particular drugs within a class, or over time.

The minimum clinically meaningful difference used for sample-size calculations is 6 points on the PDQ-39 mobility scale. This 6-point difference is based on a study of patients attending neurology clinics with PD who completed the PDQ-39 at base-line and four months later and were also asked to complete 'transition questions' at follow-up. Patients who rated themselves as worse at follow-up, whether in terms of a transition item on physical function or an item on their PD generally, experienced a mean deterioration of 7 points on the PDQ-39 mobility scale.[20] A 6-point change is used in PD MED because it translates more easily into meaningful categories, both of health states and health changes. The mobility scale has 10 items with 5 response categories (ranging from 'never' to 'always') and scores ('0' to '4') are transformed to produce a range 0-100. A 6-point change therefore results if a respondent changes three categories on one item, for example from 'being confined to the house - never' to 'being confined to the house - often'. The same change in score would also result from changing one response category - for example, from 'sometimes' to 'often' - on three of the ten items.

The main analyses in PD MED will compare changes from baseline in PDQ-39 score between groups. The standard deviation (SD) between patients of the 1-year changes in the PDQ-39 mobility dimension in early PD MED data is 18.6. This estimate appears robust, as the SD is about the same for 6-month change and for patients in the early and late randomisations, but is smaller - as, consequently, is the sample size - than the original protocol estimate of 31.6. The earlier estimate was larger because it was based on the between-patient SD seen in an unselected series of neurological clinic attendees with PD. To detect a 6-point difference (i.e. a standardised difference of 6/18.6 = 0.32) with 90% power at $p<0.01$ would require 300 patients in each arm. 155 patients in each arm would give 80% power at $p<0.05$. Thus, although it will be highly desirable for PD MED to randomise a total of 1500 early PD patients and 1000 later PD patients - to improve precision of treatment estimates and for more meaningful subgroup investigations - the study would still have good statistical power to detect small differences with about half as many patients, although subgroup analyses would then only be possible if the treatment differences were of moderate size.

Large-scale recruitment to PD MED should be feasible. There are at least 8,000 new cases of PD diagnosed in the UK each year. If just 5-6% of these were to be randomised between the early PD treatment options, then 1500 patients could be randomised in just 3-4 years. If only 3% of patients were to be entered, 900 could be randomised in the same time scale. The number of patients available for the later disease randomisation should be comparable as most patients diagnosed with PD eventually develop motor complications requiring treatment modifications. The majority of these patients are likely to have received only prior LD, so would be potentially eligible for randomisation between all 3 arms. Some patients (perhaps 20%) will have been previously treated with either a DA or MAOBI, and will only be randomised between MAOBI versus COMTI (if previous DA exposure) or between DA versus COMTI (if previous MAOBI exposure). To recruit 300 patients in each arm, about 1000 patients will need to be randomised (perhaps approximately: DA 300, MAOBI 300, COMTI 400). Again, the study would have good statistical power to detect small differences with about half as many patients.

7.2 Treatment comparisons

In the semi-factorial early disease randomisation, there will be two pre-specified comparisons:

1. LD-sparing therapy (either DA or MAOBI) versus LD alone, to determine whether LD-sparing therapy is better than LD alone.
2. DA versus MAOBI, to determine which form of LD-sparing therapy is the better.

In the later disease randomisation, there will also be two pre-specified comparisons:

3. DDI (either MAOBI or COMTI) vs DA, to determine whether DDI or DA is better.
4. COMTI versus MAOBI, to determine which form of DDI is the better.

Should one class of LD-sparing therapy or one class of DDI be clearly better than the other, then this drug class will be compared with LD alone in early disease or DA in later disease respectively.

7.3. Stratification variables

The early disease randomisation will be 'minimised' within strata defined by whether or not the patient has received previous LD therapy (none, up to one month, one to three months, three to six months), disease stage (Hoehn & Yahr stage - see Randomisation Notepad - for definitions) and by age (<50, 50-59, 60-69, 70-79, 80+ years). Prior hypotheses will be that younger patients and LD-naïve patients derive greater net benefit from LD-sparing therapy. The later disease randomisation will be minimised by age, disease stage (as above) by previous therapy (LD only, DA, MAOBI, COMTI), and by time from initial diagnosis of PD to entry (<4 years, 4-6 years, 6+ years). Subgroup analyses within randomisation strata will be undertaken. Indirect comparisons between types of DA, MAOBI and COMTI will be used to generate hypotheses for prospective testing, rather than to provide definitive answers. Because of the serious dangers of misinterpretation, all subgroup analyses will be interpreted appropriately cautiously.

7.4. Data Monitoring Committee: determining when clear answers have emerged

If any of the Parkinson's disease therapies being tested really are substantially better or worse than the others with respect to the main endpoints, or survival, then this may become apparent before the target recruitment has been reached. Alternatively, new evidence might emerge that particular drugs are definitely more, or less, effective than all, or some of, those used in the trial. To protect against this, during the period of intake of the study, interim analyses of major endpoints will be supplied, in strict confidence, to an independent Data Monitoring Committee (DMC) along with updates on results of other related studies, and any other analyses that the DMC may request. The DMC will advise the chair of the Trial Steering Committee if, in their view, any of the randomised comparisons in the trial have provided both (a) "proof beyond reasonable doubt" (see footnote B) that for all, or for some, types of patient one particular treatment is definitely indicated or definitely contraindicated in terms of a net difference in the primary outcome measures, and (b) evidence that might reasonably be expected to influence the patient management of many clinicians who are already aware of the other main trial results. The Steering Committee can then decide whether to close or modify any part of the trial.

Unless this happens, however, the Steering Committee, the collaborators and all of the central administrative staff (except the statisticians who supply the confidential analyses) will remain unaware of the interim results.

If the clinical coordinators are unable to resolve any concern satisfactorily, collaborators, and all others associated with the study, may write through the PD MED Trial Office to the chair of the DMC, drawing attention to any worries they may have about the possibility of particular side-effects, or of particular categories of patient requiring special study, or about any other matters thought relevant.

Footnote B : Appropriate criteria of proof beyond reasonable doubt cannot be specified precisely, but a difference of at least three standard deviations in an interim analysis of a major endpoint may be needed to justify halting, or modifying, the study prematurely. If this criterion were to be adopted, it would have the practical advantage that the exact number of interim analyses would be of little importance, so no fixed schedule is proposed.

8. ORGANISATION

To ensure the smooth running of the trial, and to conform with research governance requirements, it is proposed that each participating centre should designate individuals who would be chiefly responsible for local coordination of clinical and administrative aspects of the trial.

8.1. Principal Investigator at each centre

Each Centre should nominate one person to act as the Principal Investigator. Their responsibilities will include:

Acting as lead clinician for Local Research Ethics Committee (LREC) and Trust approvals for the trial on behalf of their centre: (See Section 8.4.) Once all necessary approvals have been gained, the Trial Office will send a folder containing all trial materials to the Principal Investigator. Screening and recruitment of patients into the trial can then begin.

To ensure that all medical and nursing staff involved in the care of Parkinson's disease are well informed about the study: This involves distributing protocols and patient information sheets to all relevant staff, displaying the wall-chart where it is likely to be read, and distributing the plastic protocol summaries (which can be carried in the pockets of the medical and nursing staff) and the regular newsletters. A regularly updated PowerPoint presentation will be provided for each hospital so that they can be shown from time to time, especially to new staff.

Chief nursing co-ordinator at each centre: It is suggested that each participating centre should designate one nurse as local nursing co-ordinator. This person would be responsible for ensuring that all eligible patients are considered for the study, that patients are provided with study information sheets, and have an opportunity to discuss the study if required. The nurse may be responsible for collecting the patient consent form, baseline PDQ-39, EuroQoL EQ-5D, MMSE and SF36 questionnaires. Again, this person would be sent updates and newsletters, and would be invited to training and progress meetings.

8.2. Central co-ordination

The PD MED Trial Office at the University of Birmingham Clinical Trials Unit (BCTU) is responsible for providing the trial folders containing all trial materials. These will be supplied to each collaborating centre, after relevant ethics committee approval has been obtained. Additional supplies of any printed material can be obtained on request. The Trial Office also provides the central randomisation service and is responsible for collection and checking of data (including reports of serious adverse events thought to be due to trial treatment) and for analyses.

8.3. Cost implications

The trial has been designed to minimise extra costs for participating hospitals. No extra visits to hospital need to be made, and no extra tests are required. The only extra work involved for participants will be informing patients about the study, obtaining their consent to participate, providing baseline data at randomisation, and reporting, infrequently, their progress. Centres can obtain extra support for this work from the NHS Research Support budget and the Trial Office will help them do this. Allowing clinicians to choose whichever DA, MAOBI or COMTI that they would use in their usual daily practice, means that the trial should not involve additional drug costs. Indeed, it could lead to wider use of LD and selegiline, which are considerably less expensive than any of the newer drugs, and thus could lead to substantial future cost savings if LD or selegiline are shown to be of equal or greater efficacy than the newer DAs or COMTIs.

8.4. Research Governance

The University of Birmingham is the sponsor of the PD MED trial. It has Multi-centre Research Ethics Committee (MREC) approval and Clinical Trials Authorisation from the Medicines and Healthcare Regulatory Authority (MHRA). The Trial Office will assist the Principal Investigator to obtain a site specific assessment from the local research ethics committee (LREC) and approval from the Hospital Trust.

The study will adopt a centralised approach to monitoring data quality and compliance. A computer database will be constructed specifically for the study data and will include range and logic checks to prevent erroneous data entry. The trial statistician will regularly check the balance of allocations by the stratification variables. Source data verification will only be employed if there is reason to believe data quality has been compromised, and then only in a sub-set of practices.

8.5. Indemnity

There are no special arrangements for compensation for non-negligent harm suffered by patients as a result of participating in the study. PD MED is not an industry-sponsored trial and so ABPI guidelines on indemnity do not apply. The manufacturers of the various PD therapies have not been involved in any way in the design or conduct of the trial. The normal NHS indemnity arrangements for non-negligent liability in clinician-initiated research will therefore operate. It should be noted that NHS Trust and non-Trust hospitals are responsible for any negligent liability because of their duty of care to a patient being treated within their hospital, whether or not that patient is participating in a clinical trial.

8.6. Publication and ancillary studies

A meeting will be held after the end of the study to allow discussion of the main results among the collaborators prior to publication. The success of the study depends entirely on the wholehearted collaboration of a large number of doctors, nurses and others. For this reason, chief credit for the main results will be given not to the committees or central organisers but to all those who have collaborated in the study, who will be listed as co-authors.

It is requested that any proposals for formal additional studies of the effects of the trial treatments on some patients (e.g. special investigations in selected hospitals) be referred to the Steering Committee for consideration. In general, it would be preferable for the trial to be kept as simple as possible, with very few add-on studies.

8.7. Archiving

Archiving will be authorised by BCTU on behalf of the Sponsor following submission of the end of trial report. It is the responsibility of the PI to ensure all essential trial documentation and source documents (e.g. signed ICFs, Investigator Site Files, Pharmacy Files, participants' hospital notes, copies of CRFs) at their site are securely retained as per their NHS Trust policy for at least 20 years after completion of the trial. Destruction of essential documents will require authorisation from the BCTU on behalf of the Sponsor.

9. REFERENCES

1) Quinn NP. Parkinson's disease: Clinical features. *Baillieres Clin Neurol* 1997;6:1-13.

2) Oertel WH, Quinn NP. Parkinson's disease: drug therapy. *Baillieres Clin Neurol* 1997;6:89-108.

3) Fahn S, Oakes D, Shoulson I, Kieburtz K, Rudolph A, Lang A, Olanow CW, Tanner C, Marek K; Parkinson Study Group. Levodopa and the progression of Parkinson's disease. N Engl J Med. 2004 351(24):2498-508

4) Lees AJ, Katenschlager R, Head J, Ben-Shlomo Y on behalf of the Parkinson's Disease Research Group of the United Kingdom. Ten year follow-up of three different initial treatments in de-novo PD. A randomised trial. *Neurology* 2001;57:1687-1694.

5) Rascol O, Brooks DJ, Korczyn AD, De Deyn PP, Clarke CE, Lang AE. A five-year study of the incidence of dyskinesia in patients with early Parkinson's Disease who were treated with ropinirole or levodopa. *N Engl J Med* 2000;342:1484-91.

6) Rinne UK, Bracco F, Chouza C, DuPont E, Gershanik O, Marti Masso JF, et al. Early treatment of Parkinson's disease with carbergoline delays the onset of motor complications. Results of a double-blind levodopa controlled trial. The PKDS009 Study Group. *Drugs* 1998;55 (Suppl1):23-30.

7) Parkinson Study Group. Pramipexole vs levodopa as initial treatment for Parkinson disease: A randomised controlled trial. *JAMA* 2000;284:1931-1938.

8) Wheatley K, Stowe RL, Clarke C, Hills RK, Williams AC, Gray R. Evaluating drug treatments for Parkinson's disease: how good are the trials? *BMJ* 2002;324:1508-1511.

9) Rascol O, Goetz C, Koller W, Poewe W, Sampaio C. Treatment interventions for Parkinson's disease: an evidence based assessment. *Lancet* 2002;359:1589-98.

10) Lees AJ. Comparison of therapeutic effects and mortality data of levodopa and levodopa combined with selegiline in patients with early, mild Parkinson's disease. Parkinson's Disease Research Group of the United Kingdom. *BMJ* 1995;311:1602-7.

11) Ben-Shlomo Y, Churchyard A, Head J, Hurwitz B, Overstall P, Ockelford J, et al. Investigation by Parkinson's Disease Research Group of United Kingdom into excess mortality seen with combined levodopa and selegiline treatment in patients with early, mild PD: further results of randomised trial and confidential inquiry. *BMJ* 1998;316:1191-6.

12) Parkinson Study Group. Mortality in DATATOP: A muliticentre trial in early Parkinson's disease. *Ann Neurol* 1998;43:318-25.

13) Olanow CW, Fahn S, Langston JW, Godbold J. Selegiline and mortality in Parkinson's disease. *Ann Neurol* 1996;40:841-5.

14) Maki-Ikola O, Kilkku O, Heinonen E. Effect of adding selegiline to levodopa in early, mild Parkinson's disease. *BMJ* 1996;312:720(letter).

15) Counsell C. Effect of adding selegiline to levodopa in early, mild Parkinson's disease. Formal systematic review of data on patients in all relevant trials are required. *BMJ* 1998;317:1586.

16) Ives NJ, Stowe RL, Marro J, Counsell C, Macleod A, Clarke C, Gray R, Wheatley K. Monoamine oxidase type B inhibitors in early Parkinson’s disease: a meta-analysis of 17 randomised trials involving 3525 patients. BMJ 2004;329:593-599.

17) Rascol O, Brooks DJ, Melamed E, et al. Rasagiline as an adjunct to levodopa in patients with Parkinson’s disease and motor fluctuations (LARGO, Lasting effect in Adjunct therapy with Rasagiline Given Once daily, study): a randomised double-blind parallel-group trial. Lancet 2005; 365: 947-54.

18) van Hiltern JJ, Ramaker C, van de Beek WJT, Finken MJJ. Bromocriptine for levodopa-induced complications in Parkinson's disease (Cochrane Review). In:*The Cochrane Library* Issue 3, 2002.

19) Collins R, Peto R, Gray R et al. Large-scale randomised evidence: trials and overviews. In: D. Weatherall, JGG. Ledingham, DA. Warrell eds Oxford Textbook of Medicine (3rd edn); Vol 1 Section 2.4, Oxford University Press 1996.

20) Peto V, Jenkinson C, Fitzpatrick R, Greenhall R. The development and validation of a short measure of functioning and well being for individuals with Parkinson's disease. *Qual Life Res* 1995;4:241-8.

21) Jenkinson C, Peto V, Greenhall R, Hyman N. Self-reported functioning and well-being in patients with Parkinson's disease: comparison of the short-form health survey (SF-36) and the Parkinson's Disease Questionnaire (PDQ-39). *Age & Ageing* 1995;24:505-9.

22) Fitzpatrick R, Peto V, Jenkinson C, Greenhall R, Hyman N. Health-related quality of life in Parkinson's disease: a study of outpatient clinic attenders. *Mov Disord* 1997;12:916-22.

23) Martinez-Martin P, Frades Payo B. Quality of life in Parkinson's disease: validation study of the PDQ-39 Spanish version. The Grupo Centro for Study of Movement Disorders. *J Neurol* 1998;245 (Suppl 1):S34-8.

24) Jenkinson C, Fitzpatrick R, Peto V. The Parkinson's Disease Questionnaire: User manual for the PDQ-39, PDQ-8 and PDQ Summary Index. *Health Services Research Unit*. University of Oxford, 1998.

PD MED TRIAL SCHEMA

ELIGIBILITY

Early disease randomisation: Patients with newly or recently diagnosed PD requiring medical therapy. No prior, or less than 6 months, treatment with PD medication.
Later disease randomisation: Patients with PD who develop motor complications that are uncontrolled by their current therapy: either levodopa (LD) alone or LD with the addition of a dopamine agonist (DA) or a monoamine oxidase type B inhibitor (MAOBI).
Both randomisations: Patient not demented, able to give informed consent and able to complete questionnaires.

RANDOMISATION

Randomisation is based on the "uncertainty principle". That is, if there is a definite indication for, or a definite contraindication against, a particular class of drug, then the patient is not eligible for a randomisation that includes this class of drug (Note A). If, however, the doctor is substantially uncertain which class of drug a patient should be offered, that patient is eligible to be randomised. Options are (Note B):

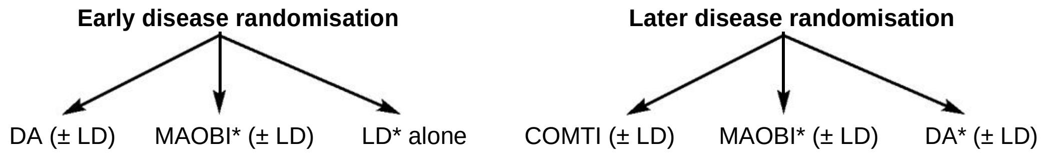

*optional arm

Note A: If one class of drug is contra-indicated the patient can still be randomised two-ways between the other two classes in both early and later disease (see protocol sections 2.2 and 2.3)

Note B: A patient who was initially entered into the early disease randomisation may also be entered into the later disease randomisation if motor complications subsequently develop

TELEPHONE RANDOMISATION

Obtain patient's consent. Administer baseline assessments (section 5.3).
Prepare for telephone questions using the randomisation notepad (see Note C).
Telephone or fax the randomisation service (contact details below).
When all the relevant questions on the randomisation notepad have been answered, a treatment allocation and patient reference number will be given.

Note C: The person randomising will need to answer all questions on the randomisation notepad.

TREATMENT

The patient should be prescribed the class of drug to which they were allocated at randomisation.
The specific drug used within this class, and drug dose and schedule, is up to each clinician's preference and local practice (Note D).
All other management is as considered appropriate by the responsible physicians.

Note D: Clinicians are referred to the Summary of Product Characteristics for further information.

FOLLOW-UP

The majority of assessments will be patient (or carer) based, with postal questionnaires at 6 months and 1 year after entry, then annually (see section 6.4)
Once a year, clinicians will be asked to fill in a simple form giving details of any changes in disease status or therapies used.

FOR RANDOMISATION, TELEPHONE (FREEPHONE IN UK): 0800 953 0274
OR +44 (0)121 415 9129 FROM OUTSIDE THE UK OR FAX 0121 415 9135
For queries, contact the PD MED Trial Office, Birmingham Clinical Trials Unit, University of Birmingham, Public Health Building, Edgbaston, Birmingham B15 2TT Tel: 0121 415 9127/9128/9129
