# Supplementary material for: Long-term Effectiveness of Adjuvant Treatment With Catechol-O-Methyltransferase or Monoamine Oxidase B Inhibitors Compared With Dopamine Agonists Among Patients With Parkinson Disease Uncontrolled by Levodopa Therapy: The PD MED Randomized Clinical Trial
Source: JAMA Neurol. 2021 Dec 28;79(2):1–10. doi: 10.1001/jamaneurol.2021.4736 (PMC8715387; doi:10.1001/jamaneurol.2021.4736)
Supplement: Supplement 2. — eTable 1. Participant Demographic and Baseline Characteristics by Randomization Strata and Protocol Comparison eTable 2. Adverse Events Experienced by Patients When Stopping Treatment eTable 3. Caregiver-Reported Quality of Life Using the SF-36 eTable 4. Elective and Nonelective Hospital Admissions for Any Reason and for PD-Related Reasons eTable 5. Nonelective Hospital Admissions eFigure 1. Clinical Trial Flowchart eFigure 2. Levodopa Dose by Allocated Treatment eFigure 3. Subgroup Analyses eFigure 4. 10-Year Risk of Nonelective Hospital Admission [file jamaneurol-e214736-s002.pdf]

# Supplemental Online Content

Gray R, Patel S, Ives N, et al; PD MED Collaborative Group. Long-term effectiveness of adjuvant treatment with catechol-O-methyltransferase or monoamine oxidase B inhibitors vs dopamine agonists among patients with Parkinson disease inadequately controlled by levodopa therapy: the PD MED randomized clinical trial. *JAMA Neurol*. Published online December 28, 2021. doi:10.1001/jamaneurol.2021.4736

**eTable 1.** Participant Demographic and Baseline Characteristics by Randomization Strata and Protocol Comparison

**eTable 2.** Adverse Events Experienced by Patients When Stopping Treatment

**eTable 3.** Caregiver-Reported Quality of Life Using the SF-36

**eTable 4.** Elective and Nonelective Hospital Admissions for Any Reason and for PD-Related Reasons

**eTable 5.** Nonelective Hospital Admissions

**eFigure 1.** Clinical Trial Flowchart

**eFigure 2.** Levodopa Dose by Allocated Treatment

**eFigure 3.** Subgroup Analyses

**eFigure 4.** 10-Year Risk of Nonelective Hospital Admission

This supplementary material has been provided by the authors to give readers additional information about their work.

© 2021 Gray R et al. *JAMA Neurology*.

**eTable 1.** Participant Demographic and Baseline Characteristics by Randomization Strata and Protocol Comparison

| | | Randomization stratum | | | DA vs. DDI comparison | | DDI comparison: MAOBI vs. COMTI | |
|---|---|---|---|---|---|---|---|---|
| | | **3-way (DA vs. MAOBI vs. COMTI) (n=236)** | **2-way (DA vs. COMTI) (n=130)** | **2-way: (MAOBI vs. COMTI) (n=134)** | **DA (N=144)** | **DDI (N=222)** | **MAOBI (N=146)** | **COMTI (N=145)** |
| **Demographics** | | | | | | | | |
| Age (years) | | 74 (41 – 91) | 73 (53 – 87) | 70 (28 – 87) | 73 (49 – 91) | 73 (41 – 88) | 72 (28 – 88) | 73 (48 – 86) |
| Men | | 154 (65%) | 79 (61%) | 81 (60%) | 90 (63%) | 143 (64%) | 91 (62%) | 95 (66%) |
| Patient has regular carer | | 163 (69%) | 76 (58%) | 91 (68%) | 86 (60%) | 153 (69%) | 99 (68%) | 109 (75%) |
| **Baseline characteristics** | | | | | | | | |
| Duration of Parkinson's disease (years) | | 4.8 (0.03 to 21.2) | 5.9 (0.3 – 20.7) | 6.6 (1.0 – 19.9) | 5.1 (0.3 – 19.7) | 5.3 (0.03 – 21.2) | 5.7 (0.5 – 21.2) | 5.7 (0.03 – 17.2) |
| Hoehn & Yahr Stage | | | | | | | | |
| | 1 to 1.5 | 27 (11%) | 19 (15%) | 11 (8%) | 19 (13%) | 27 (12%) | 13 (9%) | 16 (11%) |
| | 2 | 71 (30%) | 38 (29%) | 38 (28%) | 44 (31%) | 65 (29%) | 42 (29%) | 42 (29%) |
| | 2.5 to 5 | 138 (59%) | 73 (56%) | 85 (64%) | 81 (56%) | 130 (59%) | 91 (62%) | 87 (60%) |
| Currently on DA | | 0 (-) | 0 (-) | 133 (99%) | 0 (-) | 0 (-) | 67 (46%) | 66 (46%) |
| Currently on MAOBI | | 0 (-) | 63 (48%) | 0 (-) | 31 (22%) | 32 (14%) | 0 (-) | 0 (-) |
| PDQ-39 mobility score | | 49.3 (29.4) | 47.0 (29.7) | 52.5 (27.1) | 49.0 (29.3) | 48.1 (29.7) | 50.1 (29.1) | 50.1 (28.3) |
| PDQ-39 summary index | | 30.4 (15.3) | 30.5 (16.5) | 32.0 (14.6) | 31.6 (16.7) | 29.7 (15.0) | 29.7 (14.2) | 31.9 (15.0) |

Data are mean (range), number (%), or mean (SD). PDQ=Parkinson's disease questionnaire

© 2021 Gray R et al. *JAMA Neurology*.

**eTable 2.** Adverse Events Experienced by Patients When Stopping Treatment

| | DA | MAOBI | COMTI | p-value |
|---|---|---|---|---|
| **Side Effects[#]** | 66 | 58 | 72 | |
| – *Mental problems* | 45 | 24 | 20 | <0.001 |
| – *Sleep problems* | 7 | 8 | 6 | 0.61 |
| – *Gastrointestinal* | 3 | 5 | 29 | <0.001 |
| – *Autonomic* | 3 | 5 | 6 | 0.60 |
| – *Dizziness/Fainting* | 2 | 8 | 6 | 0.09 |
| – *Oedema* | 6 | 0 | 0 | 0.002 |
| – *Impulse control disorder* | 4 | 0 | 0 | 0.02 |
| – *Motor complications* | 2 | 6 | 6 | 0.25 |
| – *Headache* | 2 | 4 | 3 | 0.58 |
| – *Cardiac* | 2 | 3 | 0 | 0.17 |
| – *Skin* | 0 | 0 | 1 | 0.42 |
| – *Muscular* | 0 | 1 | 0 | 0.30 |
| – *Musculoskeletal* | 0 | 0 | 1 | 0.42 |
| – *Deteriorating PD* | 2 | 3 | 3 | 0.83 |
| – *Other* | 4 | 2 | 7 | 0.35 |
| – *Not stated* | 1 | 3 | 1 | 0.32 |

[#] Some patients had more than one side effect

© 2021 Gray R et al. *JAMA Neurology*.

**eTable 3.** Caregiver-Reported Quality of Life Using the SF-36

| | DA v DDI | | MAOBI v COMTI | |
|---|---|---|---|---|
| | **Estimate (95% CI)*** | **p-value** | **Estimate (95% CI)[#]** | **p-value** |
| **Domains** | | | | |
| Physical Function | -3.76 (-8.24 to 0.71) | 0.10 | 1.34 (-3.27 to 5.95) | 0.57 |
| Role Limitation due to Physical problems | -1.20 (-6.07 to 3.68) | 0.63 | -0.47 (-5.64 to 4.70) | 0.86 |
| Role Limitation due to Emotional problems | -1.89 (-7.55 to 3.78) | 0.51 | 2.92 (-2.71 to 8.56) | 0.31 |
| Social Functioning | -5.38 (-10.78 to 0.02) | 0.05 | 1.45 (-4.07 to 6.96) | 0.60 |
| Energy/Vitality | -2.35 (-6.09 to 1.38) | 0.22 | -2.86 (-6.86 to 1.14) | 0.16 |
| Pain | -0.75 (-5.81 to 4.30) | 0.77 | -3.13 (-7.98 to 1.73) | 0.21 |
| General Health Perception | -3.22 (-7.05 to 0.61) | 0.10 | -0.72 (-4.64 to 3.19) | 0.72 |
| **Summary Scores** | | | | |
| Physical Component Score | -1.31 (-3.60 to 0.99) | 0.26 | 0.01 (-2.17 to 2.20) | >0.99 |
| Mental Component Score | -0.81 (-3.11 to 1.48) | 0.48 | -0.53 (-2.79 to 1.74) | 0.65 |

* positive numbers favours DA; [#] positive numbers favours MAOBI

© 2021 Gray R et al. *JAMA Neurology*.

**eTable 4.** Elective and Nonelective Hospital Admissions for Any Reason and for PD-Related Reasons*

| **Elective admissions** | N | % |
|---|---|---|
| Zero day admissions | 549 | 72% |
| Non-zero day admissions | 215 | 28% |
| Total, all elective admissions | 764 | 43% |
| | | |
| **Non-elective admissions** | | |
| Zero day admissions | 150 | 15% |
| Non-zero day admissions | 867 | 85% |
| Total, all non-elective admissions | 1017 | 57% |
| | | |
| Subtotal, not PD-related non-elective admissions | 480 | 47% |
| PD-related admissions: | | |
| infections | 176 | 33% |
| falls, fractures or other injuries | 146 | 27% |
| Parkinson's disease | 120 | 22% |
| hypotension | 53 | 10% |
| neuropsychiatric disorders | 35 | 7% |
| other PD-related | 7 | 1% |
| Subtotal PD-related non-elective admissions | 537 | 53% |
| | | |
| **All admissions** | | |
| Total: zero day admissions | 699 | 39% |
| Total: non-zero day admissions | 1082 | 61% |
| **Total all admissions** | 1781 | |

*Inpatient episodes with exact matches on three or more variables were included. An episode was defined as the time spent under the care of one consultant, whereas an admission comprised one or more finished consultant episodes. Duplicate episodes and episodes prior to patient randomisation were excluded. Zero bed days are defined by HES as admissions where a patient was admitted and discharged within 24 hours.

© 2021 Gray R et al. *JAMA Neurology*.

**eTable 5.** Nonelective Hospital Admissions

Time to Any Admission, PD-Related Admission, or Fall-Related Admission, Number of Admissions, and Duration of Admissions by Treatment Allocation for Dopamine Agonists vs Dopamine Reuptake Inhibitors and MAO-B Inhibitors vs COMT Inhibitors

| | DA v DDI | | p-value | MAOBI vs COMTI | | p-value |
|---|---|---|---|---|---|---|
| | DA (N=134) | DDI (N=206) | | N=143 | N=139 | |
| Mean number of admissions per patient (SD) | 2.25 (2.99) | 2.34 (2.71) | 0.96[1] | 1.99 (2.38) | 2.29 (2.79) | 0.18[1] |
| Mean difference (95% C.I)* | -0.09 (-0.71, 0.52) | | | -0.29 (-0.90,0.31) | | |
| Number of zero duration admissions | 47 | 71 | | 43 | 48 | |
| Number of non-elective admission | 302 | 483 | | 285 | 318 | |
| Mean admission duration per patient [days (SD)] | 14.2 (20.9) | 14.3 (21.2) | 0.98[2] | 12.2 (18.6) | 14.7 (22.0) | 0.14[2] |
| Total admission duration per patient [days (SD)]$^{\alpha}$ | 32.1 (50.3) | 33.3 (52.1) | 0.81[2] | 24.3 (40.0) | 33.6 (53.9) | 0.10[2] |
| Number of patients who had any non-elective admission | 86 | 138 | | 95 | 91 | |
| Time to first non-elective admission: Rate Ratio (95% C.I) | 0.94 (0.72, 1.23) | | 0.65[3] | 0.94 (0.71, 1.31) | | 0.66[3] |
| Number of patients who had PD-related admissions | 63 | 110 | | 69 | 68 | |
| Time to first PD-related admission: Rate Ratio (95% C.I) | 0.86 (0.63,1.16) | | 0.32[3] | 0.93 (0.67,1.32) | | 0.69[3] |
| Number of PD related admission | 156 | 264 | | 142 | 175 | |
| Mean duration of PD related admission per patient [days (SD)] | 17.8 (23.1) | 16.7 (22.6) | 0.65[2] | 14.5 (20.1) | 17.8 (23.6) | 0.18[2] |
| Number of patients who had fall-related admissions | 22 | 49 | | 26 | 32 | |
| Time to first fall-related admission: Rate Ratio (95% C.I) | 0.68 (0.43,1.10) | | 0.11[3] | 0.75 (0.45,1.26) | | 0.28[3] |

*Negative mean difference favours DA; Ratio less than 1 favours DA; α Including patients who hadn't had an admission as if they'd had a zero day admission

[1]p-value taken from an unadjusted negative binomial model with patient years at risk included as an offset; [2]p-value taken from t-test; [3]p-value taken from log-rank test

© 2021 Gray R et al. *JAMA Neurology*.

© 2021 Gray R et al. *JAMA Neurology*.

**eFigure 1.** Clinical Trial Flowchart

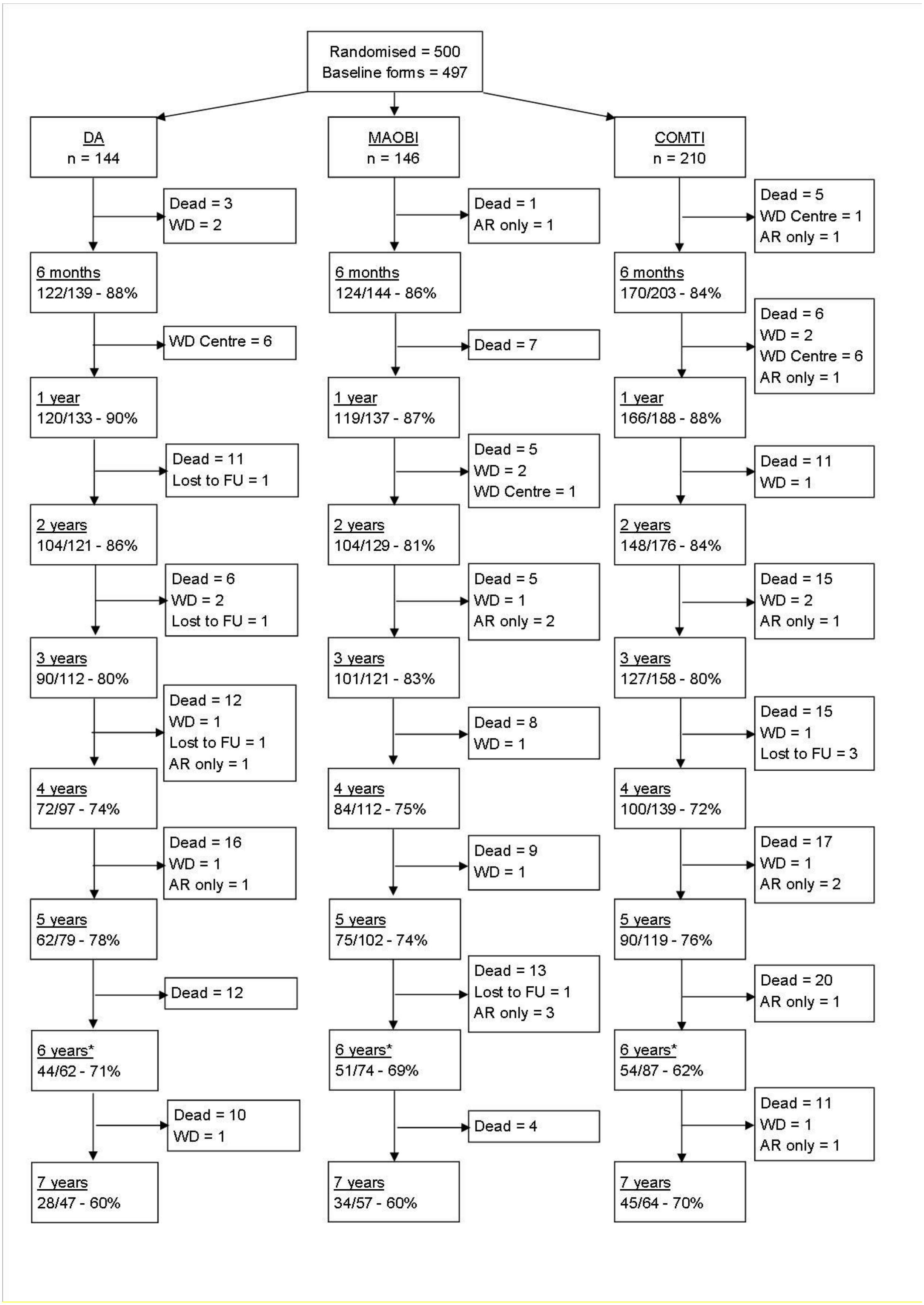


© 2021 Gray R et al. *JAMA Neurology*.

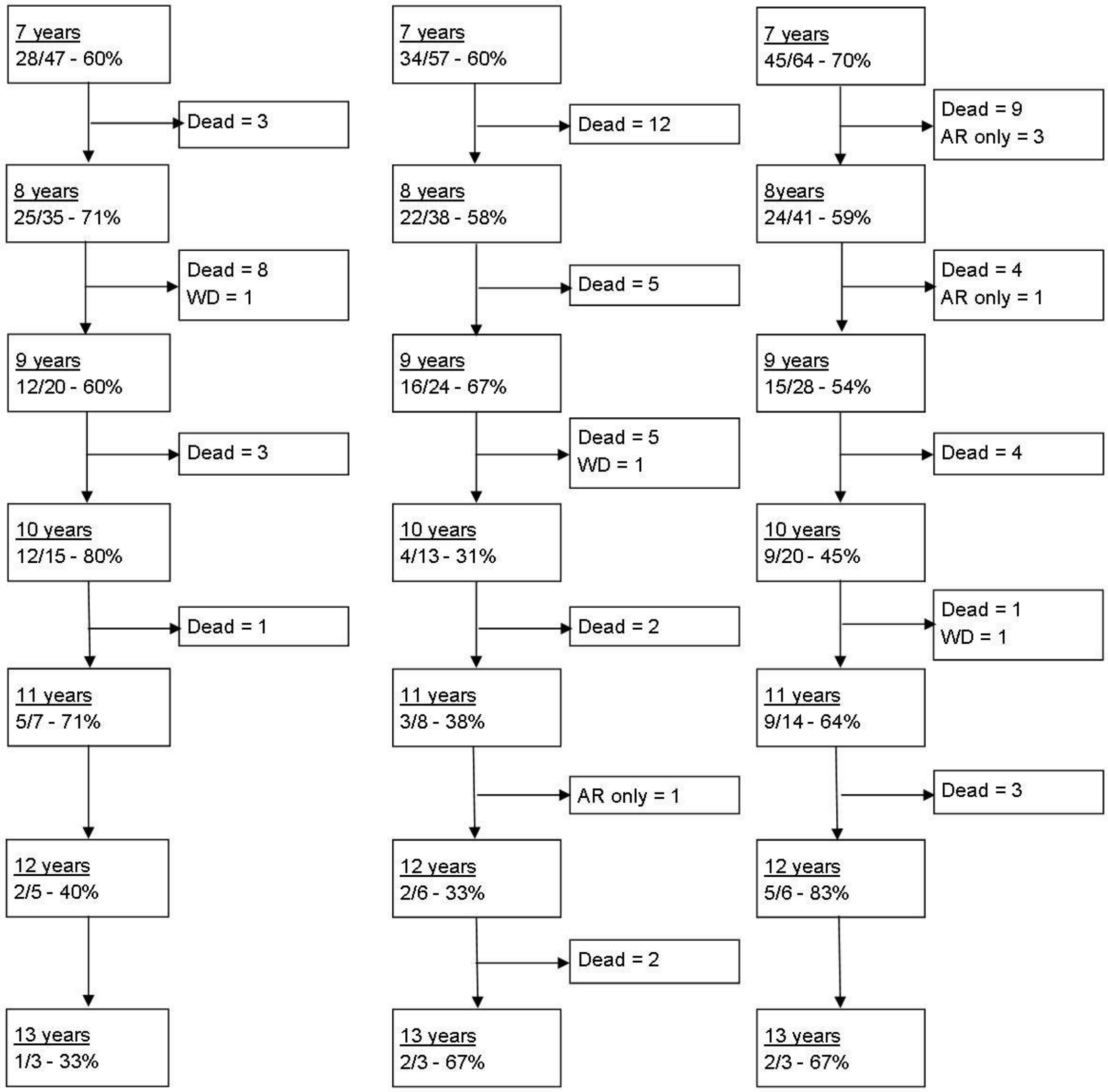


Key:
Dead = number of patients died
WD = number of patients withdrawn
Lost to FU = number of patients lost to follow-up
WD Centre = Czech/Russian patients censored following centres withdrawal from study and follow-up
AR only = patients who have withdrawn from completing patient follow-up forms but continue to have clinician assessments
* Not all patients have reached the 6 year time point

Reasons for withdrawal:
Withdrawn consent = 8
Declined to return forms = 5
Ineligible = 2
Moved away = 1
Other = 4
Unknown/not stated = 3

© 2021 Gray R et al. *JAMA Neurology*.

**eFigure 2.** Levodopa Dose by Allocated Treatment

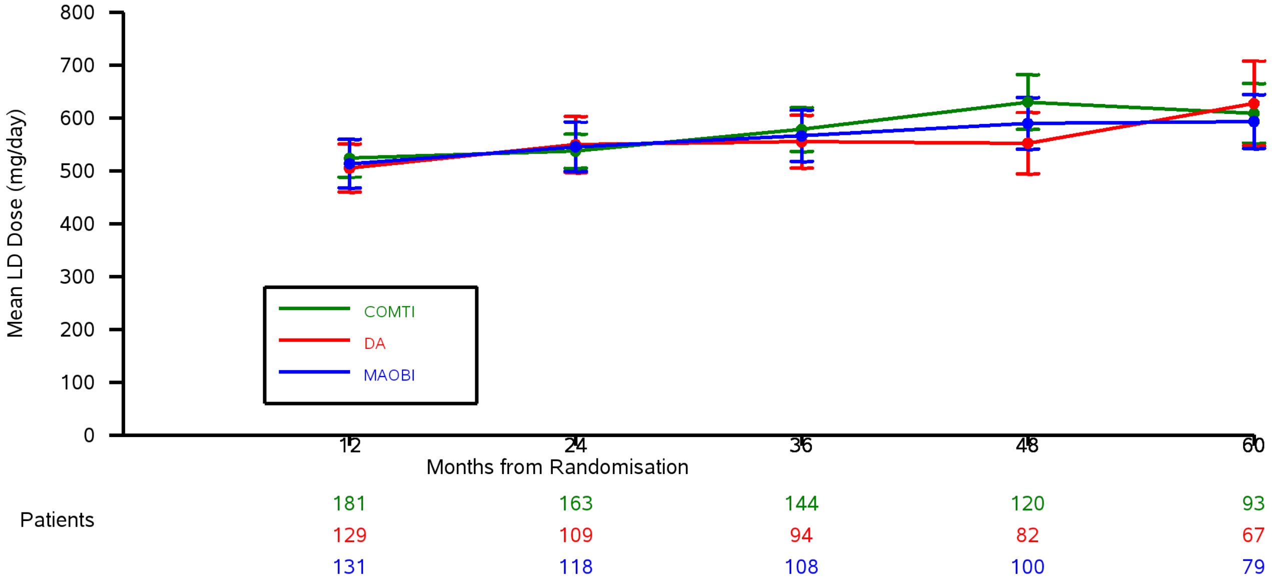


© 2021 Gray R et al. *JAMA Neurology*.

**eFigure 3.** Subgroup Analyses

A. Mean PDQ-39 mobility score over 5 years of follow-up in patients aged under and over age 70 allocated dopamine agonist and dopamine degradation inhibitor

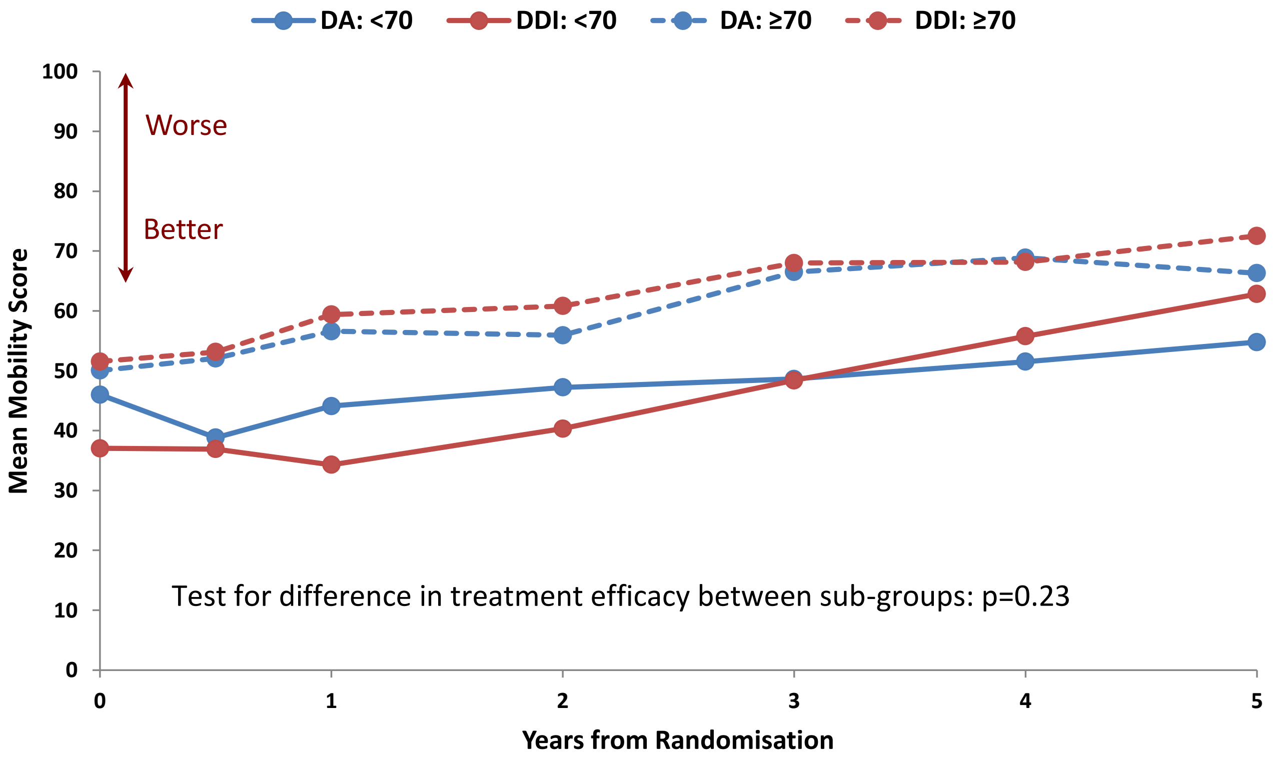


| Age | Difference* (95% CI) |
|---|---|
| Age <70 (n=90) | 6.29 (-1.21 to 13.78) |
| Age ≥70 (n=276) | 1.08 (-3.05 to 5.21) |

*Positive difference favours DA

© 2021 Gray R et al. *JAMA Neurology*.

B. Mean PDQ-39 mobility score over 5 years of follow-up in patients diagnosed less than and more than 5 years ago by treatment allocation: dopamine agonist versus dopamine degradation inhibitor

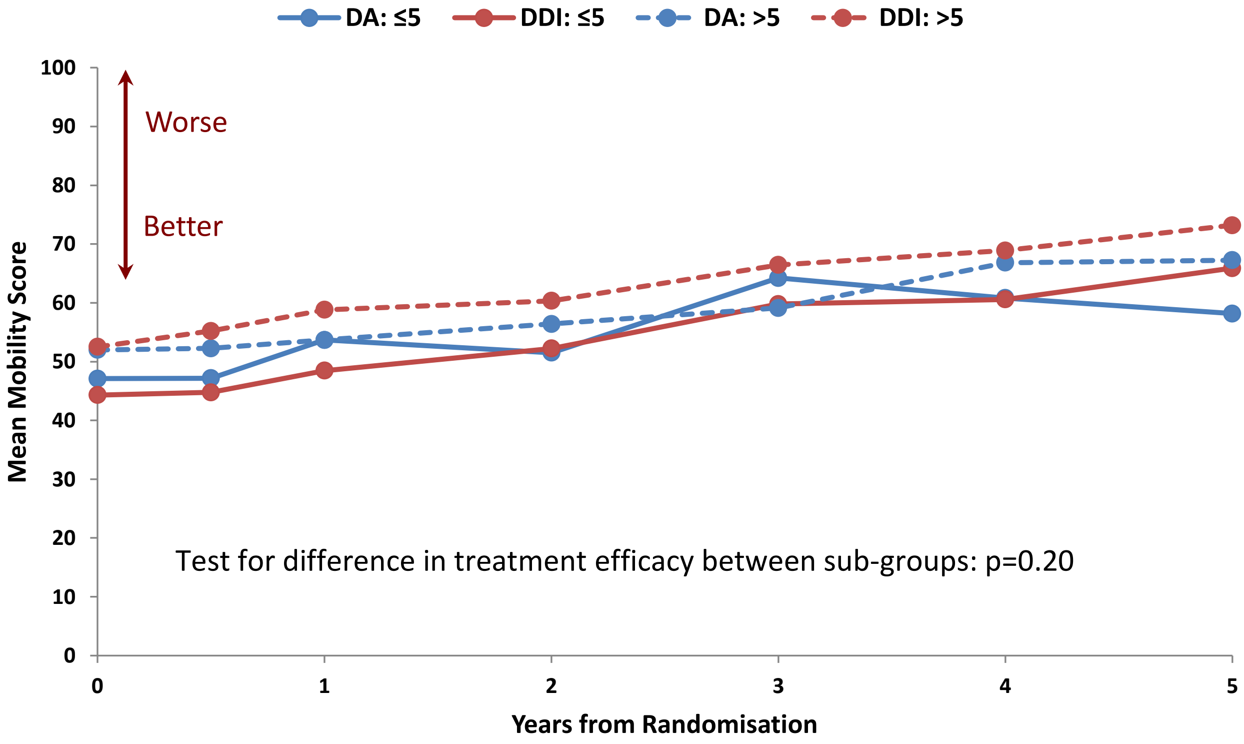


| Duration of PD | Difference* (95% CI) |
| --- | --- |
| ≤5 years (n=209) | 0.18 (-4.68 to 5.03) |
| >5 years (n=157) | 5.11 (-0.63 to 10.86) |

*Positive difference favours DA

© 2021 Gray R et al. *JAMA Neurology*.

C. Mean PDQ-39 mobility score over 5 years of follow-up in patients with Hoehn and Yahr scores 1-2.5 and 3 or more by treatment allocation: dopamine agonist versus dopamine degradation inhibitor

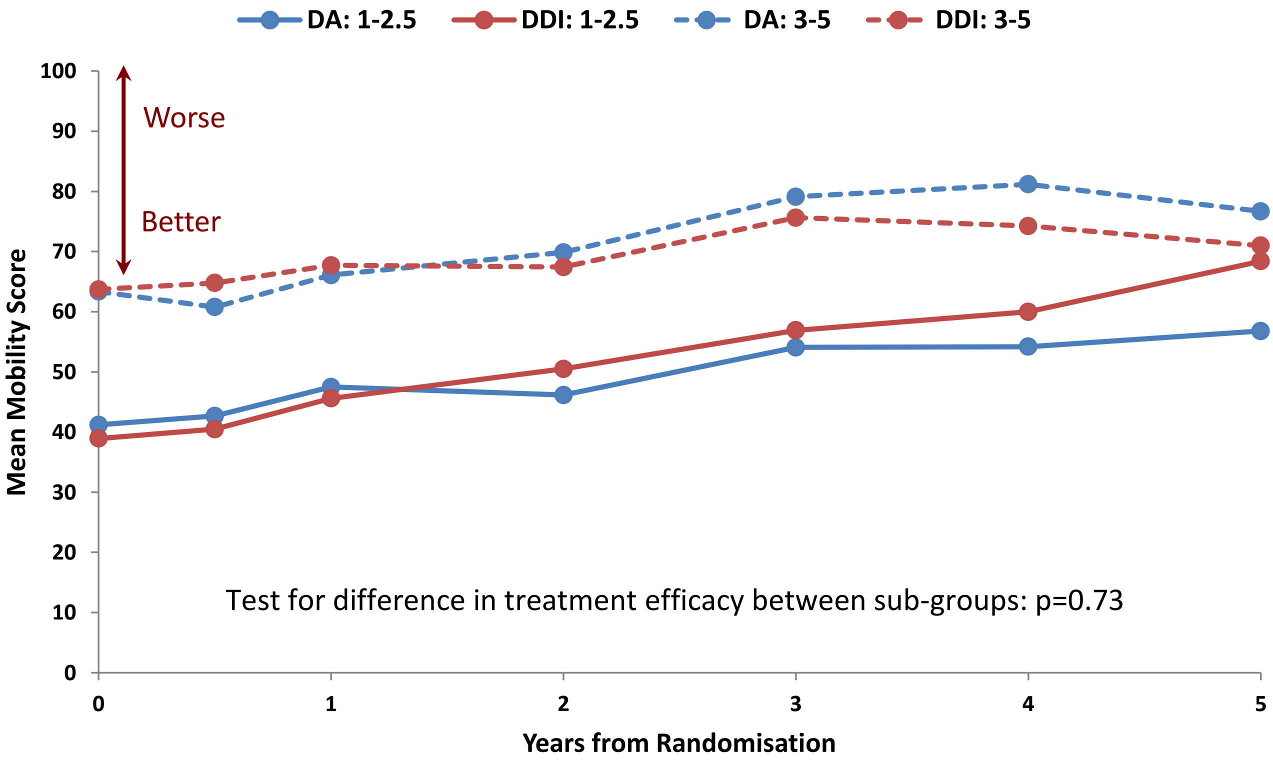


| Hoehn & Yahr score | Difference* (95% CI) |
|---|---|
| 1-2.5 (n=232) | 2.74 (-1.85 to 7.32) |
| 3-5 (n=134) | 1.40 (-4.80 to 7.61) |

*Positive difference favours DA

© 2021 Gray R et al. *JAMA Neurology*.

D. Mean PDQ-39 mobility score over 5 years of follow-up in patients randomised 2-ways and 3-ways by treatment allocation: dopamine agonist versus dopamine degradation inhibitor

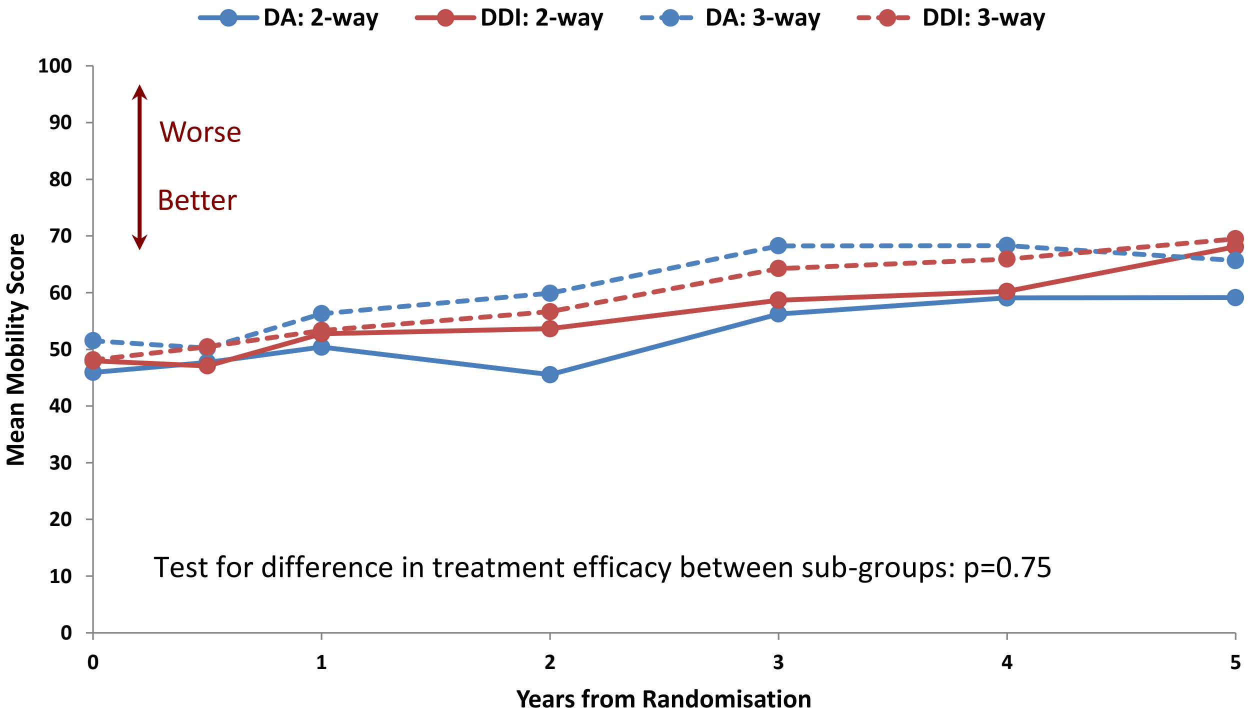


| Randomisation | Difference* (95% CI) |
|---|---|
| 2-way (n-130) | 1.48 (-4.73 to 7.68) |
| 3-way (n=236) | 2.76 (-1.97 to 7.49) |

*Positive difference favours DA

© 2021 Gray R et al. *JAMA Neurology*.

E. Mean PDQ-39 mobility score over 5 years of follow-up in patients aged under and over age 70 allocated MAOBI and COMTI

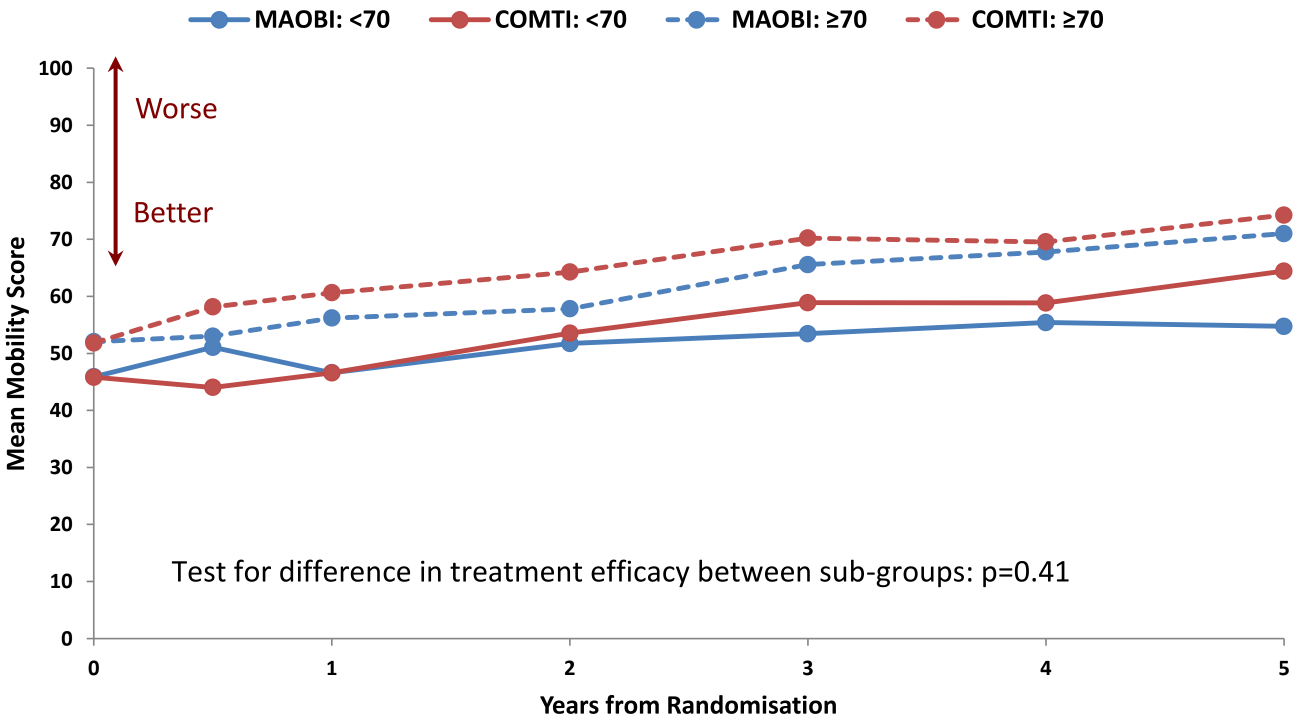


| Age | Difference* (95% CI) |
|---|---|
| Age <70 (n=88) | 1.65 (-5.18 to 8.48) |
| Age ≥70 (n=203) | 5.07 (0.45 to 9.70) |

*Positive difference favours MAOBI

© 2021 Gray R et al. *JAMA Neurology*.

F. Mean PDQ-39 mobility score over 5 years of follow-up in patients diagnosed less than and more than 5 years ago by treatment allocation: MAOBI versus COMTI

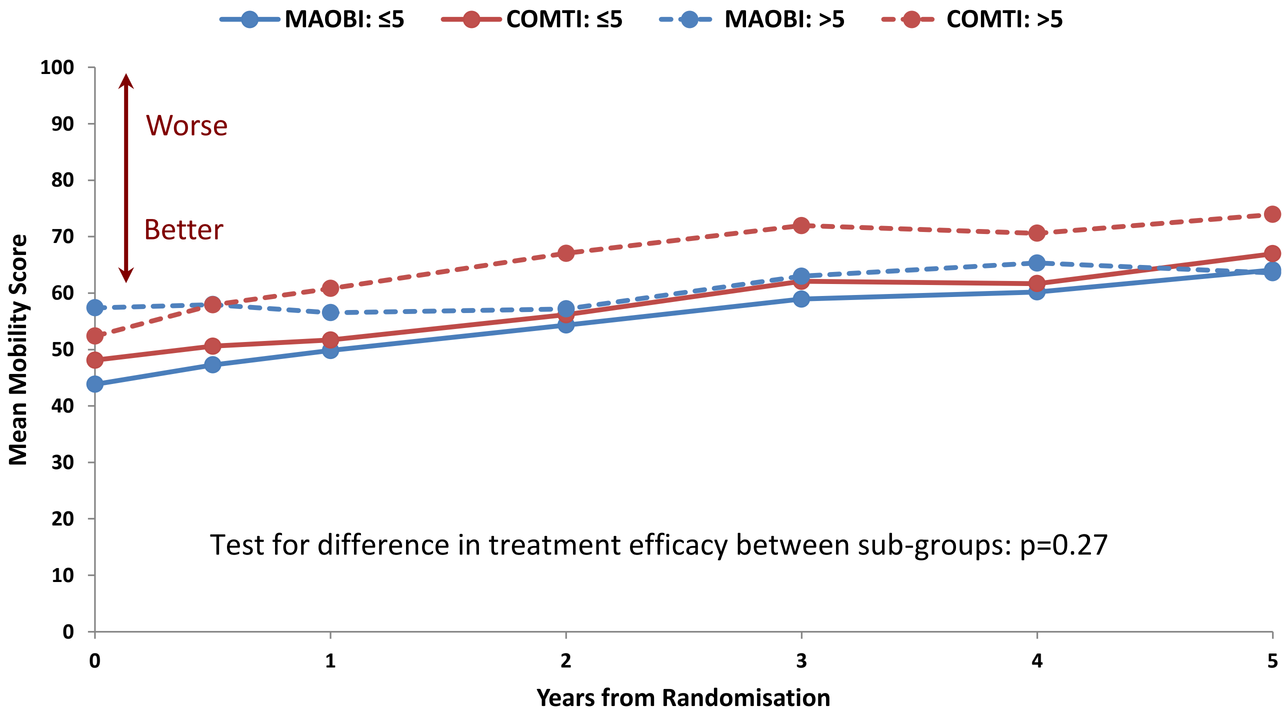


| Duration of PD | Difference* (95% CI) |
|---|---|
| ≤5 years (n=154) | 2.09 (-3.23 to 7.40) |
| >5 years (n=137) | 6.43 (0.82 to 12.03) |

*Positive difference favours MAOBI

© 2021 Gray R et al. *JAMA Neurology*.

G. Mean PDQ-39 mobility score over 5 years of follow-up in patients with Hoehn and Yahr scores 1-2.5 and 3 or more by treatment allocation: MAOBI versus COMTI

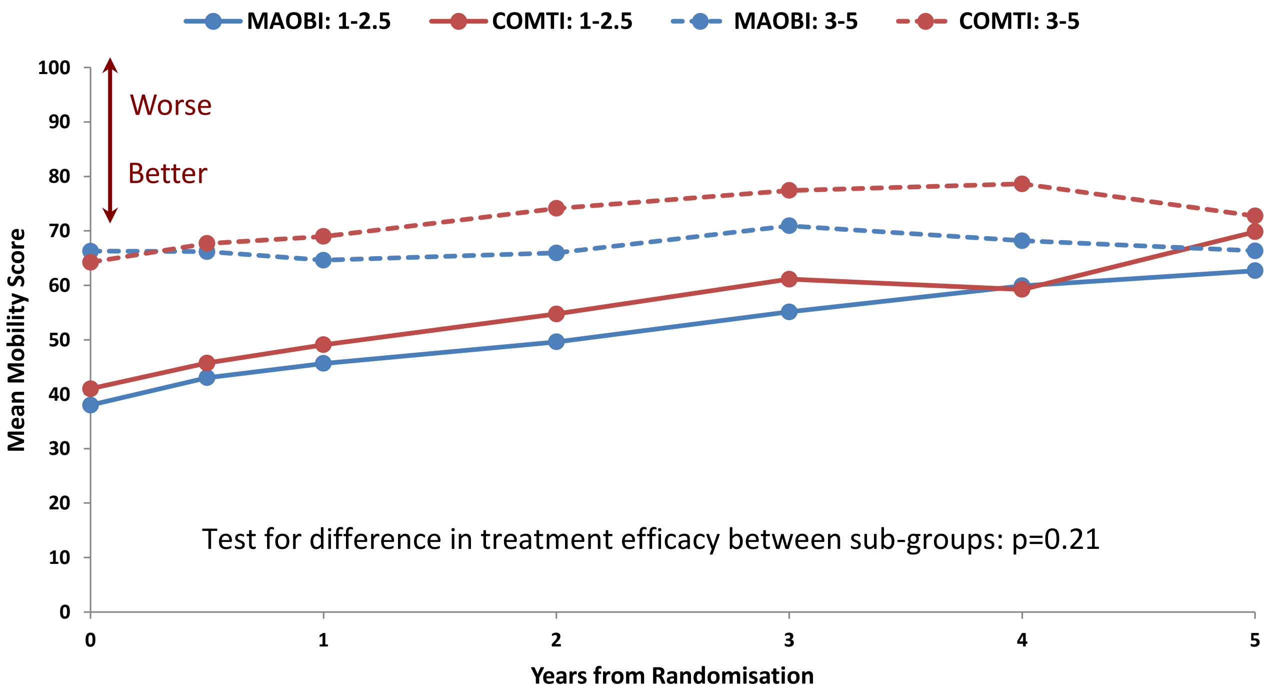


| Hoehn & Yahr score | Difference* (95% CI) |
|---|---|
| 1-2.5 (n=171) | 2.20 (-2.68 to 7.09) |
| 3-5 (n=120) | 7.26 (1.03 to 13.49) |

*Positive difference favours MAOBI

© 2021 Gray R et al. *JAMA Neurology*.

H. Mean PDQ-39 mobility score over 5 years of follow-up in patients randomised 2-ways and 3-ways by treatment allocation: MAOBI versus COMTI

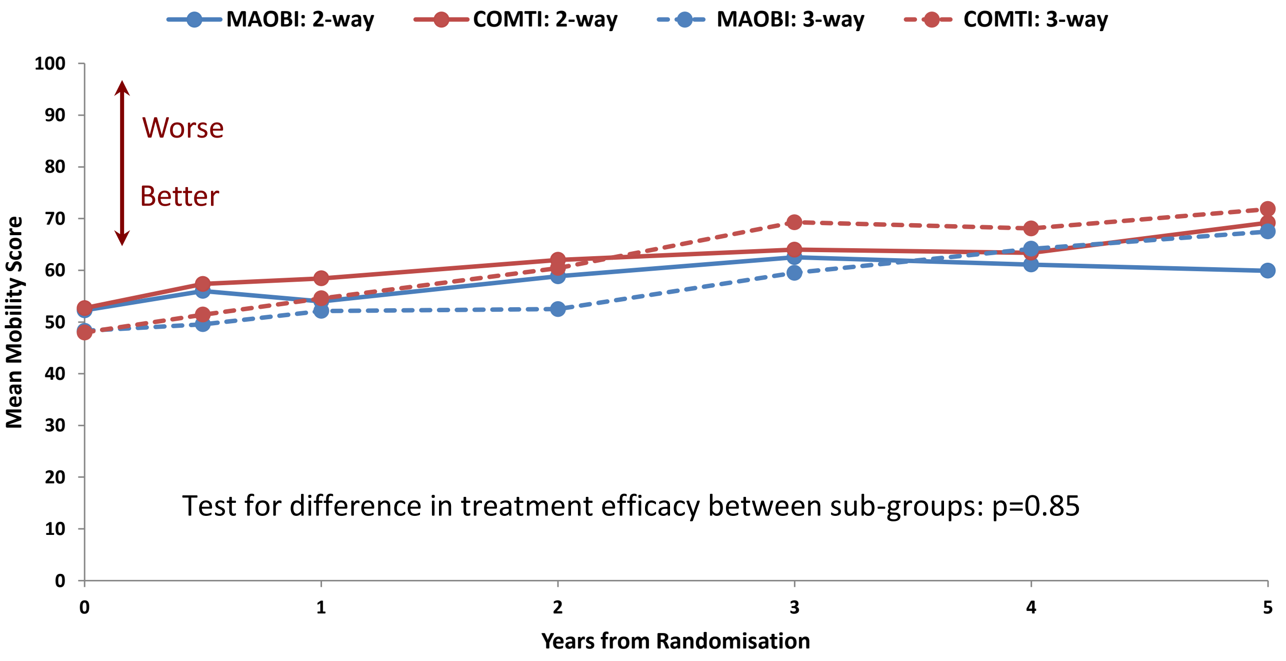


| Randomisation | Difference* (95% CI) |
|---|---|
| 2-way (n-134) | 3.78 (-1.99 to 9.54) |
| 3-way (n=157) | 4.50 (-0.69 to 9.69) |

*Positive difference favours MAOBI

© 2021 Gray R et al. *JAMA Neurology*.

**eFigure 4.** 10-Year Risk of Nonelective Hospital Admission

A. 10-year risk of non-elective hospital admission by treatment allocation: dopamine agonist versus dopamine degradation inhibitor

Kaplan-Meier graphs showing the effects of treatment allocation on 10-year risk of admission.

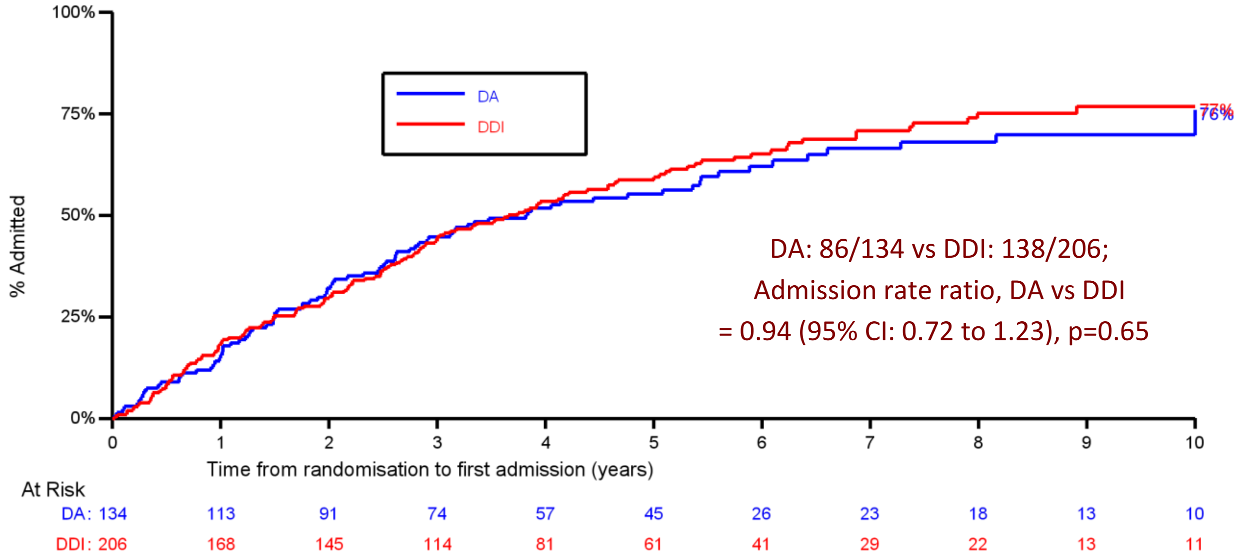


B. 10-year risk of hospital admission for PD-related condition by treatment allocation: dopamine agonist versus dopamine degradation inhibitor

Kaplan-Meier graphs showing the effects of treatment allocation on 10-year risk of admission.

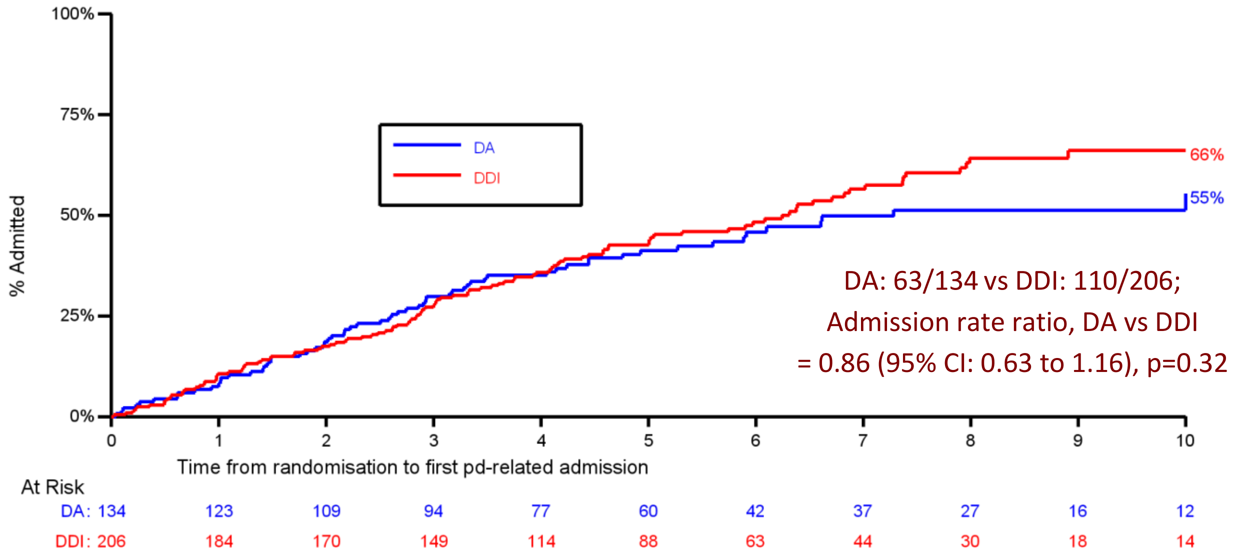


© 2021 Gray R et al. *JAMA Neurology*.

C. 10-year risk of hospital admission because of fall by treatment allocation: dopamine agonist versus dopamine degradation inhibitor

Kaplan-Meier graphs showing the effects of treatment allocation on 10-year risk of admission

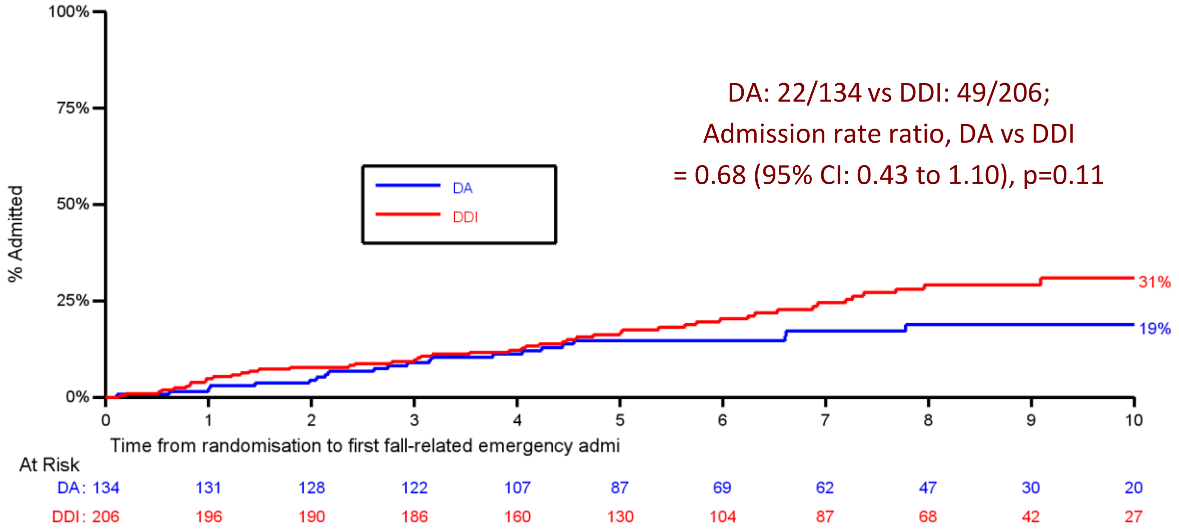


© 2021 Gray R et al. *JAMA Neurology*.

D. 10-year risk of non-elective hospital admission by treatment allocation: MAOBI versus COMTI

Kaplan-Meier graphs showing the effects of treatment allocation on 10-year risk of admission.

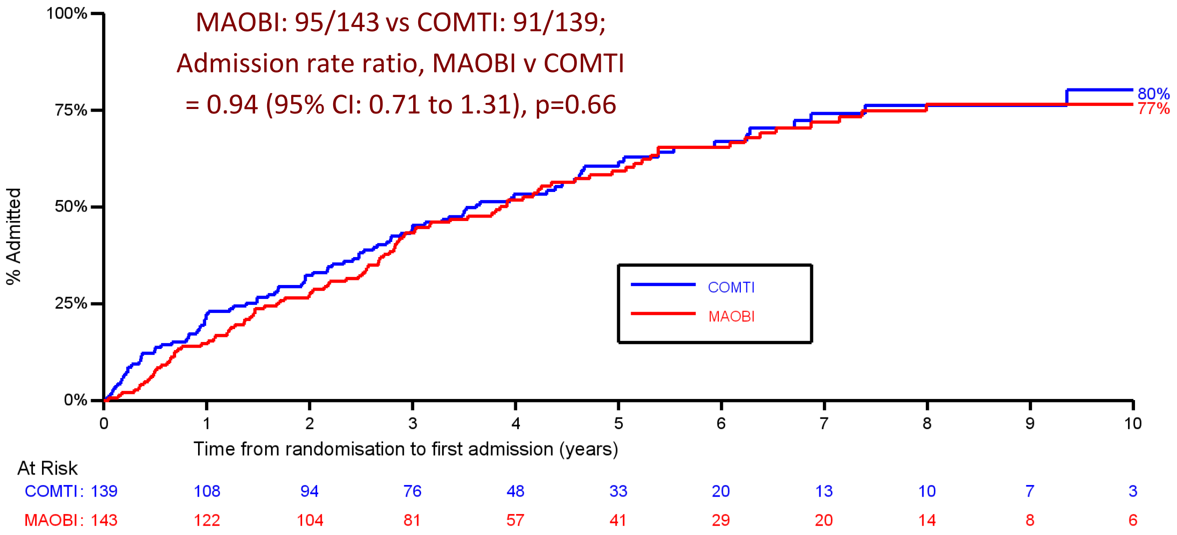


© 2021 Gray R et al. *JAMA Neurology*.

E. 10-year risk of hospital admission for PD-related cause by treatment allocation: MAOBI versus COMTI

Kaplan-Meier graphs showing the effects of treatment allocation on 10-year risk of admission.

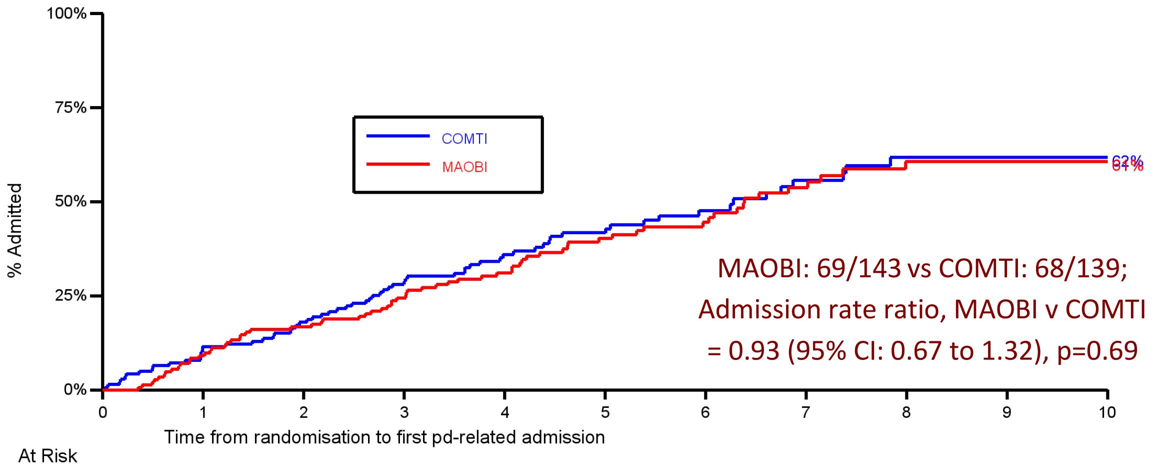


| At Risk | | | | | | | | | | | |
|---|---|---|---|---|---|---|---|---|---|---|---|
| COMTI: | 139 | 123 | 114 | 99 | 70 | 53 | 36 | 25 | 17 | 11 | 7 |
| MAOBI: | 143 | 130 | 119 | 108 | 81 | 60 | 45 | 30 | 20 | 11 | 6 |

F. 10-year risk of hospital admission because of fall by treatment allocation: MAOBI versus COMTI

Kaplan-Meier graphs showing the effects of treatment allocation on 10-year risk of admission.

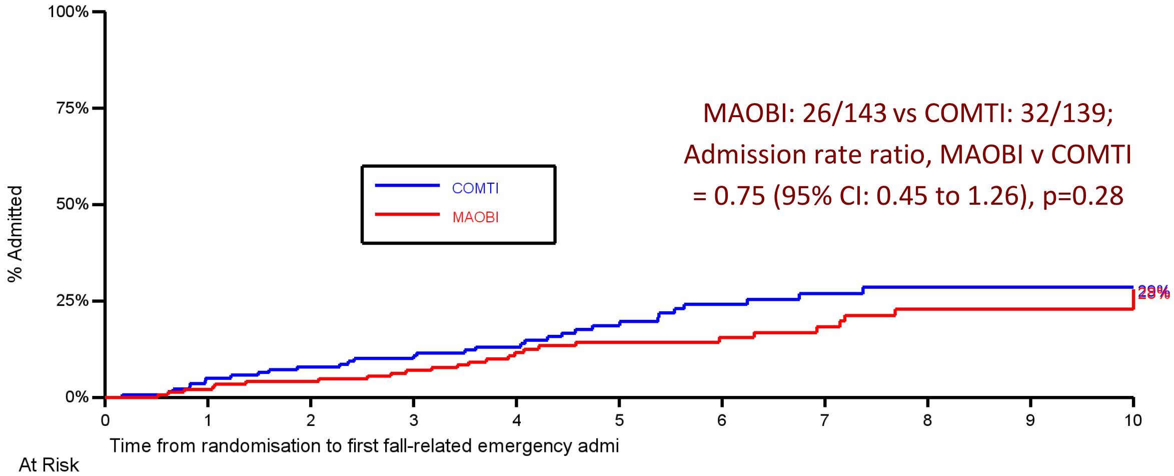


| At Risk | | | | | | | | | | | |
|---|---|---|---|---|---|---|---|---|---|---|---|
| COMTI: | 139 | 132 | 128 | 124 | 99 | 77 | 58 | 48 | 34 | 21 | 12 |
| MAOBI: | 143 | 140 | 137 | 133 | 105 | 87 | 70 | 56 | 43 | 26 | 15 |

© 2021 Gray R et al. *JAMA Neurology*.
