# Supplementary material for: Long-term Effectiveness of Adjuvant Treatment With Catechol-O-Methyltransferase or Monoamine Oxidase B Inhibitors Compared With Dopamine Agonists Among Patients With Parkinson Disease Uncontrolled by Levodopa Therapy: The PD MED Randomized Clinical Trial
Source: JAMA Neurol. 2021 Dec 28;79(2):1–10. doi: 10.1001/jamaneurol.2021.4736 (PMC8715387; doi:10.1001/jamaneurol.2021.4736)
Supplement: Supplement 3. — Data Sharing Statement [file jamaneurol-e214736-s003.pdf]

Supplemental Online Content: Nonauthor Collaborators

*Indicates required information. Only first name, last name, and suffix will appear in PubMed.

*Group Name(s): PD MED Collaborative Group

*First Name and Middle Initial(s)	*Last Name	*Suffix (eg, Jr, III)	Academic Degrees	Institution	Location (city, state/province, country)	Role or Contribution, eg, chair, principal investigator	Group (if more than 1 Group listed in the byline) and/or Subgroup (eg, Steering Committee)
Keith	Young				Birmingham, England		PPI
Helen	Price				Birmingham, England		PPI
Jon	Price				Birmingham, England		PPI
Anne	Lambert				Birmingham, England		PPI
Robin	Reeve				Birmingham, England		PPI
Martin	Sewell				UK		PPI
Sally	Broome				Birmingham, England		PPI
Adrian	Williams		MBChB	Queen Elizabeth Hospital, Birmingham, UK	Birmingham, England	Chair	Steering Committee
Mary	Baker			European Parkinson's Disease Association	UK		Steering Committee
Carl	Clarke		MBChB	Sandwell and West Birmingham Hospitals NHS Trust and University of Birmingham	Birmingham, England		Steering Committee
Ray	Fitzpatrick		BA, MA, MSc, PhD, FMedSci	University of Oxford	Oxford, England		Steering Committee
Alastair	Gray		BA, DPhil	University of Oxford	Oxford, England		Steering Committee
Richard	Greenhall		MBChB	University of Oxford	Oxford, England		Steering Committee
Crispin	Jenkinson		BA, MA, MSc, Dphil	University of Oxford	Oxford, England		Steering Committee
David	Mant		OBE, MBChB, FRCGP, FRCP, FMEDSci	University of Oxford	Oxford, England		Steering Committee
Emma	McIntosh		BA, MSc, PhD	University of Glasgow	Glasgow, Scotland		Steering Committee
Peter	Sandercock		BMBCh, MA, DM	University of Edinburgh	Edinburgh, Scotland	Chair	Data Monitoring Committee
Colin	Baugent		BMBCh, FMedSci	University of Oxford	Oxford, England		Data Monitoring Committee
Peter	Crome		MD, PhD, DSc, FRCP, FFPM	University of Keele	Keele, Newcastle, England		Data Monitoring Committee

Supplemental Online Content: Nonauthor Collaborators

*Indicates required information. Only first name, last name, and suffix will appear in PubMed.

*First Name and Middle Initial(s)	*Last Name	*Suffix (eg, Jr, III)	Academic Degrees	Institution	Location (city, state/province, country)	Role or Contribution, eg, chair, principal investigator	Group (if more than 1 Group listed in the byline) and/or Subgroup (eg, Steering Committee)
Pui	Au		BSc, MSc	University of Birmingham	Birmingham, England	Data manager	Birmingham Clinical Trials Unit
Thomas	Boodell			University of Birmingham	Birmingham, England	Trial coordinator	Birmingham Clinical Trials Unit
Versha Cheed	Cheed			University of Birmingham	Birmingham, England	Statistician	Birmingham Clinical Trials Unit
Jane	Daniels			University of Birmingham	Birmingham, England	trial coordinator	Birmingham Clinical Trials Unit
Francis	Dowling			University of Birmingham	Birmingham, England	data manager and trial coordinator	Birmingham Clinical Trials Unit
Lorraine	Evans			University of Birmingham	Birmingham, England	Trial coordinator	Birmingham Clinical Trials Unit
Robert	Hawker			University of Birmingham	Birmingham, England	Trial coordinator	Birmingham Clinical Trials Unit
Surinder	Kaur			University of Birmingham	Birmingham, England	Trial coordinator	Birmingham Clinical Trials Unit
Caroline	Rick		PhD	University of Birmingham	Birmingham, England	Team leader	Birmingham Clinical Trials Unit
Keith	Wheatley		BA, Dphil, Dipl Appl Stats	University of Birmingham	Birmingham, England	Co-investigator, Prof of statistics	Birmingham Clinical Trials Unit
Neil	Winkles		BEng	University of Birmingham	Birmingham, England	Analyst, programmer	Birmingham Clinical Trials Unit
David	Hingley			University of Birmingham	Birmingham, England	data manager	Birmingham Clinical Trials Unit
Lauren	Sturdy		BSc	University of Birmingham	Birmingham, England	data manager	Birmingham Clinical Trials Unit
Rebecca	Wooley			University of Birmingham	Birmingham, England	Statistician	Birmingham Clinical Trials Unit
Ryan	Ottridge			University of Birmingham	Birmingham, England	data manager	Birmingham Clinical Trials Unit
Lisa	Peto			University of Birmingham	Birmingham, England	data manager	Birmingham Clinical Trials Unit
Nicholas	Hilken			University of Birmingham	Birmingham, England	Analyst, programmer	Birmingham Clinical Trials Unit
Carl	Counsell		MBChB, MRCP, MD	Aberdeen Royal Infirmary	Aberdeen, Scotland	Principal investigator	

*Indicates required information. Only first name, last name, and suffix will appear in PubMed.

*First Name and Middle Initial(s)	*Last Name	*Suffix (eg, Jr, III)	Academic Degrees	Institution	Location (city, state/province, country)	Role or Contribution, eg, chair, principal investigator	Group (if more than 1 Group listed in the byline) and/or Subgroup (eg, Steering Committee)
L.	Caie			Aberdeen Royal Infirmary	Aberdeen, Scotland	Parkinson's disease specialist nurse	
R.	Caslake		MBChB	Aberdeen Royal Infirmary	Aberdeen, Scotland	Clinical Research Fellow	
Richard	Coleman		MBChB	Aberdeen Royal Infirmary	Aberdeen, Scotland	Consultant	
P.	Crowley		MBChB	Aberdeen Royal Infirmary	Aberdeen, Scotland	Consultant	
L.	Gerrie		MBChB	Aberdeen Royal Infirmary	Aberdeen, Scotland	Consultant	
J.	Gordon			Aberdeen Royal Infirmary	Aberdeen, Scotland	Research nurse	
C.	Harris			Aberdeen Royal Infirmary	Aberdeen, Scotland	Research nurse	
V.	Leslie			Aberdeen Royal Infirmary	Aberdeen, Scotland	Parkinson's disease specialist nurse	
M.A.	MacLeod		MBChB	Aberdeen Royal Infirmary	Aberdeen, Scotland	Consultant	
K.	Taylor		MBChB	Aberdeen Royal Infirmary	Aberdeen, Scotland	Clinical Research Fellow	
Paul	Worth		BMBCh, FRCP	Addenbrooke's Hospital	Cambridge, England	Prinicipal Investigator	
R.A.	Barker		MBChB	Addenbrooke's Hospital	Cambridge, England	Consultant	
Duncan	Forsyth		MBChB, MA, FRCP	Addenbrooke's Hospital	Cambridge, England	Consultant	
M.	Halls			Addenbrooke's Hospital	Cambridge, England		
J.	Young			Addenbrooke's Hospital	Cambridge, England	Parkinson's disease specialist nurse	
Wendy	Phillips		MBChB	Addenbrooke's Hospital	Cambridge, England	Consultant	
Mark	Manford		MBChB	Bedford Hospital	Bedford, England	Principal Investigator	
N.	Thangarajah		MBChB	Bedford Hospital	Bedford, England	Consultant	
Debbie	Blake			Bedford Hospital	Bedford, England	Parkinson's disease specialist nurse	
Richard	Prescott		MBChB	Bishop Aukland General Hospital	Bishop Aukland, England	Principal Investigaor	
P.	Carr		MBChB	Bishop Aukland General Hospital	Bishop Aukland, England	Consultant	

Supplemental Online Content: Nonauthor Collaborators

*Indicates required information. Only first name, last name, and suffix will appear in PubMed.

***First Name and Middle Initial(s)**	***Last Name**	***Suffix (eg, Jr, III)**	Academic Degrees	Institution	Location (city, state/province, country)	Role or Contribution, eg, chair, principal investigator	Group (if more than 1 Group listed in the byline) and/or Subgroup (eg, Steering Committee)
L.	Cochrane			Bishop Aukland General Hospital	Bishop Aukland, England	Parkinson's disease specialist nurse	
A.	Rose			Bishop Aukland General Hospital	Bishop Aukland, England	Parkinson's disease specialist nurse	
Andrew	McLaren		MBChB	Borders General Hospital	Melrose, Scotland	Principal Investigator	
M.	Drover			Borders General Hospital	Melrose, Scotland	Research nurse	
P.	Karunaratne		MBChB	Borders General Hospital	Melrose, Scotland	Associate specialist	
Duncan	Forsyth		MBChB, FRCP, MA	Brookfields Hospital	Cambridge, England	Principal Investigator	
M.	Halls			Brookfields Hospital	Cambridge, England	Parkinson's disease specialist nurse	
J.	Young			Brookfields Hospital	Cambridge, England	Parkinson's disease specialist nurse	
Amanda	Eady			Brookfields Hospital	Cambridge, England	Parkinson's disease specialist nurse	
Magdalena	Wislocka-Kryjak		MBChB	Calderdale Royal Hospital	Halifax, England	Prinicipal Investigator	
N.	Ghaus		MBBS, MRCP, FRCP	Calderdale Royal Hospital	Halifax, England	Consultant	
A.	Grueger		Dr Med	Calderdale Royal Hospital	Halifax, England	Consultant	
B.	Mallinson			Calderdale Royal Hospital	Halifax, England	Parkinson's disease specialist nurse	
G.	Wihl		Dr. Med	Calderdale Royal Hospital	Halifax, England	Consultant	
Carl	Clarke		MBChB	City Hospital, Birmingham	Birmingham, England	Prinicipal Investigator	
S.	Ballantyne		MBChB	City Hospital, Birmingham	Birmingham, England	Consultant	
S.	Hutchinson		MBChB	City Hospital, Birmingham	Birmingham, England	Consultant	
Alistair	Lewthwaite		MBChB	City Hospital, Birmingham	Birmingham, England	Consultant	
David	Nicholl		MBChB	City Hospital, Birmingham	Birmingham, England	Consultant	
A.	Ritch		MBChB	City Hospital, Birmingham	Birmingham, England	Consultant	
S.	Coyle			City Hospital, Birmingham	Birmingham, England	Parkinson's disease specialist nurse	

*Indicates required information. Only first name, last name, and suffix will appear in PubMed.

*First Name and Middle Initial(s)	*Last Name	*Suffix (eg, Jr, III)	Academic Degrees	Institution	Location (city, state/province, country)	Role or Contribution, eg, chair, principal investigator	Group (if more than 1 Group listed in the byline) and/or Subgroup (eg, Steering Committee)
R.	Hornabrook			City Hospital, Birmingham	Birmingham, England	Parkinson's disease specialist nurse	
H.	Irfan			City Hospital, Birmingham	Birmingham, England	Parkinson's disease specialist nurse	
S.	Poxon			City Hospital, Birmingham	Birmingham, England	Parkinson's disease specialist nurse	
Uma	Nath		Bmed Sci, MBBS, FRCP	City Hospital, Sunderland	Sunderland, England	Principal Investigator	
J.	Davison			City Hospital, Sunderland	Sunderland, England	Research nurse	
S.	Dodds			City Hospital, Sunderland	Sunderland, England	Research nurse	
G.	Robinson			City Hospital, Sunderland	Sunderland, England	Parkinson's disease specialist nurse	
C.	Gray			City Hospital, Sunderland	Sunderland, England		
Peter	Fletcher		MBChB, FRCP, MSc	Delancey Hospital	Cheltenham, England	Prinicipal Investigator	
P.	Morrow		MBBS, FRCP	Delancey Hospital	Cheltenham, England	Consultant	
M.	Sliva		MD, MBChB, FRCP, PhD	Delancey Hospital	Cheltenham, England	Consultant	
E.	Folkes			Delancey Hospital	Cheltenham, England	Registered nurse	
A.	Gilbert			Delancey Hospital	Cheltenham, England	Parkinson's disease specialist nurse	
H.	Hayes			Delancey Hospital	Cheltenham, England	Registered nurse	
E.	Burrows			Delancey Hospital	Cheltenham, England		
Shona	Donaldson		MBChB, MRCGP, DGM	Dumfries & Galloway Royal Infirmary	Dumfries, Scotland	Principal Investigator	
J.	Lawrence		MBChB	Dumfries & Galloway Royal Infirmary	Dumfries, Scotland	Consultant, Director R&D	
G.	Rhind		MBChB	Dumfries & Galloway Royal Infirmary	Dumfries, Scotland	Consultant	
G.	Baxter			Dumfries & Galloway Royal Infirmary	Dumfries, Scotland	R&D Director	

*Indicates required information. Only first name, last name, and suffix will appear in PubMed.

*First Name and Middle Initial(s)	*Last Name	*Suffix (eg, Jr, III)	Academic Degrees	Institution	Location (city, state/province, country)	Role or Contribution, eg, chair, principal investigator	Group (if more than 1 Group listed in the byline) and/or Subgroup (eg, Steering Committee)
J.	Bell			Dumfries & Galloway Royal Infirmary	Dumfries, Scotland	Research nurse	
J.	Gorman			Dumfries & Galloway Royal Infirmary	Dumfries, Scotland	Research nurse	
Sunku	Guptha		MBBS, FRCP	Peterborough City Hospital	Peterborough, England	Principal Investigator	
C.	Noble			Peterborough City Hospital	Peterborough, England	Parkinson''s disease specialist nurse	
John	Hindle		MBChB	Eryri Hospital	Caenarfon, Wales	Principal Investigator	
S.	Jones		MBChB	Eryri Hospital	Caenarfon, Wales	Consultant	
P.	Ohri		MBChB	Eryri Hospital	Caenarfon, Wales	Consultant	
R.	Subashchandran		MBChB	Eryri Hospital	Caenarfon, Wales	Consultant	
E.	Roberts			Eryri Hospital	Caenarfon, Wales	Parkinson's disease speciialist nurse	
Jason	Raw		MBChB, MSc	Fairfield General Hopsital	Bury, England	Prinicipal Investigator	
U.	Wadhwa		MBChB, MSc	Fairfield General Hopsital	Bury, England	Consultant	
L.	Aspden			Fairfield General Hopsital	Bury, England	Research nurse	
L.	Partington			Fairfield General Hopsital	Bury, England	Research nurse	
H.	Vanek			Fairfield General Hopsital	Bury, England	Research nurse	
Alan	Whone		MBChB, FRCP, PhD	Southmead Hospital	Bristol, England	Principal Investigator	
Ronald	Barber		MBChB	Southmead Hospital	Bristol, England	Consultant	
Beverly	Haywood			Southmead Hospital	Bristol, England	Research nurse	
Peter	Heywood		MBChB	Southmead Hospital	Bristol, England	Consultant	
Helen	Lewis			Southmead Hospital	Bristol, England	Research nurse	
Karen	O'Sullivan			Southmead Hospital	Bristol, England	Parkinson's disease specialist nurse	
K.	Prout			Southmead Hospital	Bristol, England	Parkinson's disease specialist nurse	
Liz	Whelan			Southmead Hospital	Bristol, England		
Pippa	Medcalf		MBChB, FRCP	Gloucester Royal Hospital	Gloucester, England	Principal Investigator	

Supplemental Online Content: Nonauthor Collaborators

*Indicates required information. Only first name, last name, and suffix will appear in PubMed.

***First Name and Middle Initial(s)**	***Last Name**	***Suffix (eg, Jr, III)**	Academic Degrees	Institution	Location (city, state/province, country)	Role or Contribution, eg, chair, principal investigator	Group (if more than 1 Group listed in the byline) and/or Subgroup (eg, Steering Committee)
Peter	Fletcher		MBChB, FRCP, MSc	Gloucester Royal Hospital	Gloucester, England	Consultant	
M	Sliva		MD, MBChB, FRCP, PhD	Gloucester Royal Hospital	Gloucester, England	Consultant	
G.	Fuller		MBChB, FRCP	Gloucester Royal Hospital	Gloucester, England	Consultant	
P.	Morrish		MBChB, FRCP	Gloucester Royal Hospital	Gloucester, England	Consultant	
E.	Folkes			Gloucester Royal Hospital	Gloucester, England	Registered nurse	
Emma	Wales		MBBS	Hereford County Hospital	Hereford, England	Principal Investigator	
J.	Dalziel		MBChB	Hereford County Hospital	Hereford, England		
P.	Overstall		MBChB	Hereford County Hospital	Hereford, England		
K.	Bouifraden			Hereford County Hospital	Hereford, England	Parkinson's disease specialist nurse	
Caroline	Evans			Hereford County Hospital	Hereford, England	Parkinson's disease specialist nurse	
G.	Ward			Hereford County Hospital	Hereford, England	Research nurse	
P.	Matheson			Hereford County Hospital	Hereford, England	Research nurse	
Timothy	Lockington		MD, FRCP	Ipswich Hospital	Ipswich, England	Principal Investigator	
A.	Graham		MBChB	Ipswich Hospital	Ipswich, England	Consultant	
S.F.M.	Grimmer		MBChB	Ipswich Hospital	Ipswich, England	Consultant	
L.J.	Sheehan			Ipswich Hospital	Ipswich, England		
H.	Williams			Ipswich Hospital	Ipswich, England		
Irene	Hubbard		MBChB, FRCP	Kettering General Hospital	Kettering, England	Principal Investigator	
R.	Walters		MBChB	Kettering General Hospital	Kettering, England	Consultant	
R.	Glasspool			Kettering General Hospital	Kettering, England	Parkinson's disease specialist nurse	
Peter	Critchley		MD, MBChB, FRCP	Leicester General Hospital and Leicester Royal Infirmary	Leicester, England	Principal Investigator	
R.	Abbott		MBChB	Leicester General Hospital and Leicester Royal Infirmary	Leicester, England	Consultant	
B.	Kendall		MBChB	Leicester General Hospital and Leicester Royal Infirmary	Leicester, England	Consultant	

*Indicates required information. Only first name, last name, and suffix will appear in PubMed.

***First Name and Middle Initial(s)**	***Last Name**	***Suffix (eg, Jr, III)**	Academic Degrees	Institution	Location (city, state/province, country)	Role or Contribution, eg, chair, principal investigator	Group (if more than 1 Group listed in the byline) and/or Subgroup (eg, Steering Committee)
M.	Lawden		MBChB	Leicester General Hospital and Leicester Royal Infirmary	Leicester, England	Consultant	
N.	Lo		MBChB	Leicester General Hospital	Leicester, England	Consultant	
Y.	Rajaally		MBChB	Leicester General Hospital and Leicester Royal Infirmary	Leicester, England	Consultant	
B.	Simpson		MBChB	Leicester General Hospital	Leicester, England	Consultant	
J.	Martey			Leicester General Hospital and Leicester Royal Infirmary	Leicester, England	Parkinson's disease specialist nurse	
L. Goodwin	Wray			Leicester General Hospital	Leicester, England	PD Counselor	
John	Hindle		MBChB	Llandudno General Hospital	Llandudno, Wales	Principal Investigator	
M.	Omar		MBChB	Llandudno General Hospital	Llandudno, Wales	Associate Specialist	
Avinash	Sharma		MBChB	Luton & Dunstable University Hospital	Luton, England	Principal Investigator	
A.	Gale		MBChB	Luton & Dunstable University Hospital	Luton, England	Consultant	
D.	Phirii		MBChB	Luton & Dunstable University Hospital	Luton, England	Consultant	
L.	Sekaran		MBChB	Luton & Dunstable University Hospital	Luton, England	Consultant	
S.	Wijayasiri		MBBS, FRCP	Luton & Dunstable University Hospital	Luton, England	Consultant	
Monty	Silverdale		MD, PhD, FRCP	Macclesfield District General Hospital	Macclesfield, England	Principal Investigator	
D.	Walker		MBChB	Macclesfield District General Hospital	Macclesfield, England	Consultant	
H.	Fleary			Macclesfield District General Hospital	Macclesfield, England	Research nurse	
A.	Monaghan			Macclesfield District General Hospital	Macclesfield, England		

*Indicates required information. Only first name, last name, and suffix will appear in PubMed.

*First Name and Middle Initial(s)	*Last Name	*Suffix (eg, Jr, III)	Academic Degrees	Institution	Location (city, state/province, country)	Role or Contribution, eg, chair, principal investigator	Group (if more than 1 Group listed in the byline) and/or Subgroup (eg, Steering Committee)
H.	Vanek			Macclesfield District General Hospital	Macclesfield, England	Research nurse	
Viswanathan	Senthil		MBBS, MD, FRCP	Manor Hospital	Walsall, England	Principal Investigator	
S.	Reynolds			Manor Hospital	Walsall, England	Parkinson's disease specialist nurse	
M. S.	Chong		MBBS, MD, FRCP	Medway Maritime Hospital	Gillingham, England	Principal Investigator	
D.	Diem		MBChB	Medway Maritime Hospital	Gillingham, England	Consultant	
B.	Kundu		MBChB	Medway Maritime Hospital	Gillingham, England	COnsultant	
D.	Arnold			Medway Maritime Hospital	Gillingham, England	Parkinson's disease speciaist nurse	
N.	Quinn		MBChB	National Hospital for Neurology & Neuroscience	London, England	Principal Investigator	
H.	Benamer		MBChB	New Cross Hospital	Wolverhampton, England	Principal Investigator	
J.	Billings		MBChB	New Cross Hospital	Wolverhampton, England	Consultant	
R.	Corston		MBChB	New Cross Hospital	Wolverhampton, England	Consultant	
D.	D'Costa		MBBS, FRCP	New Cross Hospital	Wolverhampton, England	Consultant	
M.	Green			New Cross Hospital	Wolverhampton, England	Research nurse	
J.	Shuri			New Cross Hospital	Wolverhampton, England	Parkinson's disease specialist nurse	
J.M.	Noble		MbBChir, PhD, FRCP	Newcastle General Hospital	Newcastle-upon-Tyne, England	Principal Investigator	
T.	Cassidy		MBChB	Newcastle General Hospital	Newcastle-upon-Tyne, England	Consultant	
A.	Gani		MBChB, MD, MRCP	Newcastle General Hospital	Newcastle-upon-Tyne, England	Consultant	
S.	Dodds			Newcastle General Hospital	Newcastle-upon-Tyne, England	Research nurse	
R.	Lawson			Newcastle General Hospital	Newcastle-upon-Tyne, England	Research nurse	

Supplemental Online Content: Nonauthor Collaborators

*Indicates required information. Only first name, last name, and suffix will appear in PubMed.

*First Name and Middle Initial(s)	*Last Name	*Suffix (eg, Jr, III)	Academic Degrees	Institution	Location (city, state/province, country)	Role or Contribution, eg, chair, principal investigator	Group (if more than 1 Group listed in the byline) and/or Subgroup (eg, Steering Committee)
Alagaratnam	Nirubin		MBChB, MSc	Norfolk & Norwich University Hospital	Norwich, England	Principal Investigator	
J.	Cochius		MBBS, FRCAP	Norfolk & Norwich University Hospital	Norwich, England	Consultant	
D.	Dick		MBChB, MD, FRCP	Norfolk & Norwich University Hospital	Norwich, England	Consultant	
M.	Lee		MBChB	Norfolk & Norwich University Hospital	Norwich, England	Consultant	
B.	Payne		MBChB	Norfolk & Norwich University Hospital	Norwich, England	Consultant	
M.	Roche		MBChB	Norfolk & Norwich University Hospital	Norwich, England	Consultant	
K.	Sabanathan		MBChB	Norfolk & Norwich University Hospital	Norwich, England	Consultant	
S.	Shields		MBChB	Norfolk & Norwich University Hospital	Norwich, England	Consultant	
Paul	Worth		BMBCh Oxen, FRCP	Norfolk & Norwich University Hospital	Norwich, England	Consultant	
M.	Hipperson			Norfolk & Norwich University Hospital	Norwich, England	Parkinson's disease specialist nurse	
F.	Reading			Norfolk & Norwich University Hospital	Norwich, England	Parkinson's disease specialist nurse	
Julia	Saunders		MBChB	North Devon District Hospital	Barnstaple, England	Principal Investigator	
G.	Harper		MBChB	North Devon District Hospital	Barnstaple, England	Consultant	
W.	Honan		MBChB	North Devon District Hospital	Barnstaple, England	Consultant	
L.	Gill			North Devon District Hospital	Barnstaple, England	Parkinson's disease specialist nurse	
J.	Stanley			North Devon District Hospital	Barnstaple, England	Parkinson's disease specialist nurse	
Nathan	Vernon			North Devon District Hospital	Barnstaple, England	Research nurse	

Supplemental Online Content: Nonauthor Collaborators

*Indicates required information. Only first name, last name, and suffix will appear in PubMed.

***First Name and Middle Initial(s)**	***Last Name**	***Suffix (eg, Jr, III)**	Academic Degrees	Institution	Location (city, state/province, country)	Role or Contribution, eg, chair, principal investigator	Group (if more than 1 Group listed in the byline) and/or Subgroup (eg, Steering Committee)
A.	Skinner			North Devon District Hospital	Barnstaple, England	Research nurse	
P.	McCann		MBChB	N Manchester General Hospital	Manchester, England	Principal Investigator	
Richard	Walker		MBChB, FRCP	N Tyneside General Hospital	North Shields, England	Principal Investigator	
P.	Edmonds		MBChB	N Tyneside General Hospital	North Shields, England	Consultant	
S.	O'Hanlon		MBChB	N Tyneside General Hospital	North Shields, England	Consultant	
Brian	Wood		MBChB	N Tyneside General Hospital	North Shields, England	Consultant	
A.	Hand			N Tyneside General Hospital	North Shields, England	Research nurse	
L.	Robinson			N Tyneside General Hospital	North Shields, England	Research nurse	
Jane	Liddle		MBBS, FRCP	Northern General Hospital	Sheffield, England	Principal Investigator	
D.	Bolam			Northern General Hospital	Sheffield, England	Parkinson's disease specialist nurse	
Sandip	Raha		MBChB, FRCP	Princess of Wales Hospital	Glamorgan, Wales	Principal Investigator	
Louise	Ebebezer			Princess of Wales Hospital	Glamorgan, Wales	Parkinson's disease specialist nurse	
S.	Thompson			Princess of Wales Hospital	Glamorgan, Wales	Research nurse	
Adrian	Williams		MBChB	Queen Elizabeth Hospital Birmingham	Birmingham, England	Principal Investigator	
H.	Benamer		MBChB	Queen Elizabeth Hospital Birmingham	Birmingham, England	Consultant	
David	Nicholl		MBChB	Queen Elizabeth Hospital Birmingham	Birmingham, England	Consultant	
H.	Pall		MBChB	Queen Elizabeth Hospital Birmingham	Birmingham, England	Consultant	
P.	Praamstra		MBChB	Queen Elizabeth Hospital Birmingham	Birmingham, England	Consultant	
Rose	Crouch			Queen Elizabeth Hospital Birmingham	Birmingham, England	Parkinsons' disease specialist nurse	

*Indicates required information. Only first name, last name, and suffix will appear in PubMed.

*First Name and Middle Initial(s)	*Last Name	*Suffix (eg, Jr, III)	Academic Degrees	Institution	Location (city, state/province, country)	Role or Contribution, eg, chair, principal investigator	Group (if more than 1 Group listed in the byline) and/or Subgroup (eg, Steering Committee)
Karen	Healy			Queen Elizabeth Hospital Birmingham	Birmingham, England	Parkinsons' disease specialist nurse	
R.	Hornabrook			Queen Elizabeth Hospital Birmingham	Birmingham, England	Research nurse	
M.	Johnson			Queen Elizabeth Hospital Birmingham	Birmingham, England	Parkinsons' disease specialist nurse	
Michael	Jenkinson		MBChB, FRCP	Queen Elizabeth, the Queen Mother Hospital	Margate, England	Principal Investigator	
A.	Abdel-Hafiz		MBChB	Rotherham General Hospital	Rotherham, England	Principal Investigator	
F.	Al-Modaris		MBChB	Rotherham General Hospital	Rotherham, England	Consultant	
S.	Dutta		MBChB	Rotherham General Hospital	Rotherham, England	COnsultant	
T.	Mallik		MBBS	Rotherham General Hospital	Rotherham, England	Consultant	
B.	Mondal		MBChB	Rotherham General Hospital	Rotherham, England	COnsultant	
J.	Roberts			Rotherham General Hospital	Rotherham, England	Parkinson's disease specialist nurse	
S.	Sinha		MBChB	Rotherham General Hospital	Rotherham, England	Consultant	
Khaled	Amar		MD, FRCP	Royal Bournemouth General Hospital	Bournemouth, England	Principal Investigator	
S.	Atkins			Royal Bournemouth General Hospital	Bournemouth, England	Parkinson's disease specialist nurse	
G.	Devadason			Royal Bournemouth General Hospital	Bournemouth, England		
A.	Martin			Royal Bournemouth General Hospital	Bournemouth, England	Parkinson's disease specialist nurse	
C.	Cox			Royal Bournemouth General Hospital	Bournemouth, England	Parkinson's disease specialist nurse	
Timothy	Malone		MB, BS, DA, FRCA	Royal Devon & Exeter Hospital	Exeter, England	Principal Investigator	
G.	Fenwick		MBChB	Royal Devon & Exeter Hospital	Exeter, England	Consultant	
K.	Gormley		MBChB	Royal Devon & Exeter Hospital	Exeter, England	Consultant	
N.	Gutowski		MBChB	Royal Devon & Exeter Hospital	Exeter, England	Consultant	

*Indicates required information. Only first name, last name, and suffix will appear in PubMed.

*First Name and Middle Initial(s)	*Last Name	*Suffix (eg, Jr, III)	Academic Degrees	Institution	Location (city, state/province, country)	Role or Contribution, eg, chair, principal investigator	Group (if more than 1 Group listed in the byline) and/or Subgroup (eg, Steering Committee)
S.	Harris		MBChB	Royal Devon & Exeter Hospital	Exeter, England	Consultant	
T.	Harrower		MBChB	Royal Devon & Exeter Hospital	Exeter, England	Consultant	
A.	Hemsley		MBChB	Royal Devon & Exeter Hospital	Exeter, England	Consultant	
M.	James		MBChB	Royal Devon & Exeter Hospital	Exeter, England	Consultant	
M.O.	Jeffreys		MBChB	Royal Devon & Exeter Hospital	Exeter, England	Consultant	
Vaughn	Pearce		MBChB	Royal Devon & Exeter Hospital	Exeter, England	Consultant	
Raymond	Sheridan		MBChB	Royal Devon & Exeter Hospital	Exeter, England	Consultant	
J.	Sword		MBChB	Royal Devon & Exeter Hospital	Exeter, England	Consultant	
A.	Zeman		MBChB	Royal Devon & Exeter Hospital	Exeter, England	Consultant	
W.	Honan		MBChB	Royal Devon & Exeter Hospital	Exeter, England	Consultant	
C.	Soper			Royal Devon & Exeter Hospital	Exeter, England	Parkinson's disease specialist nurse	
Joseph	Vassallo		MBChB	Royal Oldham Hospital	Oldham, England	Principal Investigator	
J.	Bennett			Royal Oldham Hospital	Oldham, England		
Veronica	Lyell		MBChB, FRCP	Royal United Hospital	Bath, England	Principal Investigator	
D.	Robertson		MBChB	Royal United Hospital	Bath, England	Consultant	
D.	Howcroft			Royal United Hospital	Bath, England	Research nurse	
K.	Mugweni			Royal United Hospital	Bath, England	Clinical trials officer	
A.	Stephens			Royal United Hospital	Bath, England		
E.	Whelan			Royal United Hospital	Bath, England	Research nurse	
A.	Wright			Royal United Hospital	Bath, England	Clinical trials officer	
J.	Chamberlain			Royal United Hospital	Bath, England	Parkinson's disease specialist nurse	
Diran	Padiachy		MBBcH, MRCP	Salisbury District Hospital	Salisbury, England	Principal Investigator	
J.	Marigold		MBChB	Salisbury District Hospital	Salisbury, England	Consultant	
J.	Chamberlain			Salisbury District Hospital	Salisbury, England	Parkinson's disease specialist nurse	

Supplemental Online Content: Nonauthor Collaborators

*Indicates required information. Only first name, last name, and suffix will appear in PubMed.

*First Name and Middle Initial(s)	*Last Name	*Suffix (eg, Jr, III)	Academic Degrees	Institution	Location (city, state/province, country)	Role or Contribution, eg, chair, principal investigator	Group (if more than 1 Group listed in the byline) and/or Subgroup (eg, Steering Committee)
J.	Lee			Salisbury District Hospital	Salisbury, England	Parkinson's disease specialist nurse	
Helen	Roberts		MBChB, FRCP, PhD	Southampton General Hospital	Southampton, England	Principal Investigator	
J.	Adams		MBChB	Southampton General Hospital	Southampton, England	Consultant	
J.	Dulay		MBChB	Southampton General Hospital	Southampton, England	Consultant	
S.	Evans		MBChB	Southampton General Hospital	Southampton, England	Consultant	
J.	Frankel		MBChB	Southampton General Hospital	Southampton, England	Consultant	
R.	Gove		MBChB	Southampton General Hospital	Southampton, England	Consultant	
G.	Turner		MBChB	Southampton General Hospital	Southampton, England	Consultant	
N.	Mallik		MBChB	Southampton General Hospital	Southampton, England	Consultant	
Tracey	McElwaine			Southampton General Hospital	Southampton, England	Parkinson's disease specialist nurse	
S.	Morgan			Southampton General Hospital	Southampton, England		
H.	Phipps			Southampton General Hospital	Southampton, England	Parkinson's disease specialist nurse	
V.	Pressly			Southampton General Hospital	Southampton, England	Research nurse	
V.	Queen			Southampton General Hospital	Southampton, England	Parkinson's disease specialist nurse	
R.	Tan			Southampton General Hospital	Southampton, England	Research nurse	
Donald	Grossett		MBChB, MD	Southern General Hospital	Glasgow, Scotland	Principal Investigator	

Supplemental Online Content: Nonauthor Collaborators

*Indicates required information. Only first name, last name, and suffix will appear in PubMed.

*First Name and Middle Initial(s)	*Last Name	*Suffix (eg, Jr, III)	Academic Degrees	Institution	Location (city, state/province, country)	Role or Contribution, eg, chair, principal investigator	Group (if more than 1 Group listed in the byline) and/or Subgroup (eg, Steering Committee)
G.	Macphee		MBChB, MD	Southern General Hospital	Glasgow, Scotland	Consultant	
Carol	Vennard			Southern General Hospital	Glasgow, Scotland	Parknson's disease specialist nurse	
Irena	Rektorova		PhD	CEITEC, Masaryk University and St Anne's Hospital	Czech Republic	Research Group Leader	
Zahid	Dhakam		MD	St. Peter's Hospital	Chertsey, England	Principal Investigator	
G.	Carey			St. Peter's Hospital	Chertsey, England	Parkinson's disease specialist nurse	
B.	Castledon		MBChB	St. Peter's Hospital	Chertsey, England	Consultant	
C.	Sunderland			St. Peter's Hospital	Chertsey, England	Research nurse	
E.	Kalcantera			St. Peter's Hospital	Chertsey, England	Research nurse	
C.	Long		MBChB	St. Peter's Hospital	Chertsey, England	Consultant	
B.	Mandal		MBChB	St. Peter's Hospital	Chertsey, England	Consultant	
V.	Martin			St. Peter's Hospital	Chertsey, England	Parkinson's disease specialist nurse	
R.	Nari		MBChB	St. Peter's Hospital	Chertsey, England	Consultant	
V.	Nicholas			St. Peter's Hospital	Chertsey, England		
Virginia	Moffitt		MB, BS, MRCP	St. Richard's Hospital	Chichester, England	Principal Investigator	
S.	Hammans		MA, MD	St. Richard's Hospital	Chichester, England	Consultant	
M.	Rice-Oxley		MB, MS, FRCP	St. Richard's Hospital	Chichester, England	Consultant	
Julie	Webb			St. Richard's Hospital	Chichester, England	Parkinson's disease specialist nurse	
S.	Franks			St. Richard's Hospital	Chichester, England	Parkinson's disease specialist nurse	
Simon	Cooper		MBChB, FRCP	Musgrove Park Hospital	Taunton, England	Principal Investigator	
M.	Hussain		MBChB, FRCP	Musgrove Park Hospital	Taunton, England	Consultant	
T.	Solanki		MBChB, FRCP	Musgrove Park Hospital	Taunton, England	Consultant	
W.	Darch			Musgrove Park Hospital	Taunton, England	Parkinson's disease specialist nurse	
J.	Homan			Musgrove Park Hospital	Taunton, England	Research nurse	
D.	Sharratt			Musgrove Park Hospital	Taunton, England	Research nurse	
Gareth	Griggs		MBChB, MRCP	Torbay Hospital	Torquay, England	Principal Investigator	

Supplemental Online Content: Nonauthor Collaborators

*Indicates required information. Only first name, last name, and suffix will appear in PubMed.

*First Name and Middle Initial(s)	*Last Name	*Suffix (eg, Jr, III)	Academic Degrees	Institution	Location (city, state/province, country)	Role or Contribution, eg, chair, principal investigator	Group (if more than 1 Group listed in the byline) and/or Subgroup (eg, Steering Committee)
G.	Kendall		MBChB	Torbay Hospital	Torquay, England	Consultant	
A.	Ford			Torbay Hospital	Torquay, England	Parkinson's disease specialist nurse	
K.	Stocker			Torbay Hospital	Torquay, England		
Lucy	Strens		MBChB	Walsgrave Hospital	Coventry, England	Principal Investigator	
A.	Grubneac		MBChB	Walsgrave Hospital	Coventry, England	Consultant	
J.	Ponsford		MBChB	Walsgrave Hospital	Coventry, England	Consultant	
L.	Teare		MBChB	Walsgrave Hospital	Coventry, England	Consultant	
Austen Peter	Moore		MBChB, MD, FRCP	The Walton Centre	Liverpool, England	Principal Investigator	
I.	O'Brien			The Walton Centre	Liverpool, England	Parkinson's disease specialist nurse	
D.	Watling			The Walton Centre	Liverpool, England	Research Unit Manager	
L.	Wyatt			The Walton Centre	Liverpool, England	Parkinson's disease specialist nurse	
Syed	Rizvi		MBBS, FRCP	Watford General Hospital	Watford, England	Principal Investigator	
Elaine	Walker			Watford General Hospital	Watford, England	Research nurse	
Paul	Worth		BMBCh Oxen, FRCP	West Suffolk Hospital	Bury St Edmunds, England	Principal Investigator	
G.	Berry			West Suffolk Hospital	Bury St Edmunds, England		
N.	Russell			West Suffolk Hospital	Bury St Edmunds, England		
Khalid	Rashed		MBChB	Yeovil Hospital	Yeovil, England	Principal Investigator	
K.	Baker		MBChB	Yeovil Hospital	Yeovil, England	Consultant	
M.R.	Qadiri		MBChB	Yeovil Hospital	Yeovil, England	Consultant	
C.	Buckley			Yeovil Hospital	Yeovil, England	Research nurse	
S.	Bulley			Yeovil Hospital	Yeovil, England	Research nurse	
D.	Gibbons			Yeovil Hospital	Yeovil, England	Research nurse	
R.	Goodland			Yeovil Hospital	Yeovil, England	Parkinson's disease specialist nurse	
P.	Heywood			Yeovil Hospital	Yeovil, England	Research nurse	
L.	Jones			Yeovil Hospital	Yeovil, England	Research nurse	

Supplemental Online Content: Nonauthor Collaborators

*Indicates required information. Only first name, last name, and suffix will appear in PubMed.

***First Name and Middle Initial(s)**	***Last Name**	***Suffix (eg, Jr, III)**	Academic Degrees	Institution	Location (city, state/province, country)	Role or Contribution, eg, chair, principal investigator	Group (if more than 1 Group listed in the byline) and/or Subgroup (eg, Steering Committee)
L.	Martin			Yeovil Hospital	Yeovil, England	Research nurse	
R.	Rowland-Axe			Yeovil Hospital	Yeovil, England	Research nurse	
A.	Stone			Yeovil Hospital	Yeovil, England	Parkinson's disease specialist nurse	
M.R.	Whittuck			Yeovil Hospital	Yeovil, England		
