# Supplementary material for: Long-term Effectiveness of Adjuvant Treatment With Catechol-O-Methyltransferase or Monoamine Oxidase B Inhibitors Compared With Dopamine Agonists Among Patients With Parkinson Disease Uncontrolled by Levodopa Therapy: The PD MED Randomized Clinical Trial
Source: JAMA Neurol. 2021 Dec 28;79(2):1–10. doi: 10.1001/jamaneurol.2021.4736 (PMC8715387; doi:10.1001/jamaneurol.2021.4736)
Supplement: Supplement 4. — Nonauthor Collaborators. The PD MED Collaborative Group [file jamaneurol-e214736-s004.pdf]

Data Sharing Statement

Gray. Long-term Effectiveness of Adjuvant Treatment With Catechol-O-Methyltransferase or Monoamine Oxidase B Inhibitors Compared With Dopamine Agonists Among Patients With Parkinson Disease Uncontrolled by Levodopa Therapy. *JAMA Neurol*. Published December 28, 2021. doi:10.1001/jamaneurol.2021.4736

Data
Data available: Yes
Data types: Deidentified participant data
How to access data: n.j.ives@bham.ac.uk
When available: With publication

Supporting Documents
Document types: None

Additional Information
Who can access the data: researchers whose proposed use of the data has been approved
Types of analyses: For scientifically sound, approved projects
Mechanisms of data availability: after approval of a proposal, with a signed data access agreement
